# First genome sequence and functional profiling of *Psychrobacter* SC65A.3 preserved in 5,000-year-old cave ice: insights into ancient resistome, antimicrobial potential, and enzymatic activities

**DOI:** 10.3389/fmicb.2025.1713017

**Published:** 2026-02-17

**Authors:** Victoria Ioana Paun, Corina Itcus, Paris Lavin, Mariana Carmen Chifiriuc, Cristina Purcarea

**Affiliations:** 1 Department of Microbiology, Institute of Biology Bucharest of the Romanian Academy, Bucharest, Romania; 2 Departamento de Biotecnologıa, Facultad de Ciencias del Mar y Recursos Biologicos, Universidad de Antofagasta, Antofagasta, Chile; 3 Centro de Investigación en Inmunología y Biotecnología Biomédica de Antofagasta (CIIBBA), Universidad de Antofagasta, Antofagasta, Chile; 4 Faculty of Biology and the Research Institute of the University of Bucharest, ICUB, University of Bucharest, Bucharest, Romania

**Keywords:** *Psychrobacter*, whole genome sequence, ancient ice resistome, functional profile, Scarisoara Ice Cave

## Abstract

**Introduction:**

Ancient cryospheric environments may preserve overlooked reservoirs of antimicrobial resistance (AMR) and bioactive potential. This study reports the first whole-genome sequencing and functional characterization of Psychrobacter sp. SC65A.3 isolated from 5,000-year-old ice from Scărișoara Ice Cave, revealing a multidrug-resistance phenotype alongside antimicrobial activity.

**Methods:**

Whole-genome sequencing combined with phenotypic characterization for extremotolerance, antibiotic susceptibility and biochemical profile were used to identify and functionally characterize the ancient Psychrobacter sp. SC65A.3.

**Results:**

SC65A.3 is a polyextremophile, growing up to 15 °C and tolerating 1.9 M NaCl and 0.9 M MgCl₂. Phylogenetic analysis classified it within P. cryohalolentis. Functional assays showed broad hydrolytic activity and resistance to 10 antibiotics across 8 classes, including third-generation cephalosporins, fluoroquinolones, aminoglycosides, and rifampicin. Whole-genome analysis identified >100 AMR-associated genes, including clinically relevant determinants (e.g., ampC, gyrA, gyrB, parC, parE, dfrA, rpoB, tetA, tetC, and mcr-1), as well as multiple heavy-metal resistance and multidrug efflux genes. SC65A.3 inhibited 14 ESKAPE-group pathogens (including MRSA, Enterococcus faecium, Enterobacter sp., Pseudomonas aeruginosa, Klebsiella pneumoniae, and Acinetobacter baumannii), consistent with genes linked to antimicrobial compounds such as glycopeptides and bacitracin. In addition, 45 stress-response genes related to cold/heat adaptation were detected, including distinctive htpX, htpG, and pka genes among cold-adapted Psychrobacter.

**Discussion:**

SC65A.3 represents an ancient, ice-adapted Psychrobacter with a dual profile of multidrug resistance and antimicrobial activity, highlighting ice caves as underexplored reservoirs of ancient resistomes and bioactive traits. To our knowledge, this is the first genome analysis of a Psychrobacter isolate from an ice cave and the first characterization of an ancient resistome from this environment, supporting future ecological, biotechnological, and medical exploration.

## 1 Introduction

With 20% of Earth’s surface comprising frozen habitats and low temperatures characterizing much of the biosphere, understanding cold-adapted microbes is increasingly critical in the context of rapid climate change (Margesin and Miteva, 2011; Yadav et al., 2017). Icy habitats serve as reservoirs for psychrophilic microorganisms representing a significant source of genomic diversity and novel microbial species, that exhibit remarkable stability and activity at low temperatures, holding promising applications in bio-nanotechnology and various industries (Priscu et al., 1998, 1999; Miteva et al., 2009; Anesio and Laybourn-Parry, 2012; Anesio et al., 2017; Mondini et al., 2022).

Recent studies have explored microbiomes in diverse cave systems (Bogdan et al., 2023; Gatinho et al., 2024; Jurado et al., 2024; Ogorek et al., 2024; Salazar-Hamm et al., 2025), including perennial ice deposits within caves (Paun et al., 2021; Mondini et al., 2022; Lange-Enyedi et al., 2024). However, investigations of cave-associated icy habitats remain comparatively sparse in contrast to the substantial literature on permafrost (Zhang et al., 2007; McCann et al., 2016; Rivkina et al., 2016; Abramov et al., 2021), polar ice sheets (Rehakova et al., 2010; Musilova et al., 2015; Mogrovejo et al., 2020; Kumar and Sharma, 2021), sea ice (Deming, 2009; Wing et al., 2012; Li et al., 2019a; Ozturk et al., 2022), glacial lakes (Murray et al., 2012; Wang et al., 2016; Sharma and Kumar, 2017; Gupta et al., 2022), and snow (Bachy et al., 2011; Lopatina et al., 2016; Chen et al., 2022). Expanding research on cave ice deposits active microbiomes is essential to uncover their biodiversity, functional adaptations to geochemical and climatic variations, and their potential as a source of novel microbial strains and biomolecules for industrial and medical applications (Purcarea, 2018).

Scarisoara Ice Cave (Romania) is among the most extensively studied ice caves globally. Its 100,000 m^3^ perennial ice block, dated to approximately 13,000 years before present (BP), is one of the oldest and largest of its kind (Holmlund et al., 2005; Persoiu and Pazdur, 2010; Hubbard, 2017; Persoiu et al., 2017; Paun et al., 2019). The molecular approaches revealed that its perennial ice deposit hosts a rich and complex microbial community (Hillebrand-Voiculescu et al., 2013, 2014; Itcus et al., 2018; Mondini et al., 2019, 2022; Paun et al., 2019). The distribution of the microbial community appears to be influenced by the climatic conditions during ice formation and the organic carbon content within the ice substrate (Itcus et al., 2016, 2018; Brad et al., 2018; Mondini et al., 2019; Paun et al., 2019). Culture-based microbiological studies performed on Scarisoara Ice Cave allowed the isolation and characterization of psychrotrophic and psychrophilic bacterial strains (Paun et al., 2021).

In addition to their ecological and biotechnological relevance, microbial communities preserved in ancient ice deposits are also valuable for understanding the evolutionary history of antimicrobial resistance (AMR). AMR is an ancient, natural phenomenon, occurring long before clinical antibiotic use, with resistance mechanisms such as target alteration, drug efflux/influx control, and enzyme-mediated inactivation evolving over millions of years (Allen et al., 2010; D’Costa et al., 2011; Bhullar et al., 2012; Perry et al., 2016; Van Goethem et al., 2018; Morozova et al., 2022). However, this natural phenomenon has been accelerated by the huge selection pressure exerted by chronic antibiotic use, promoting antibiotic resistance genes (ARGs) diversification and spreading through horizontal gene transfer (HGT) (Kaufmann and Hung, 2010; Kohanski et al., 2010; D’Costa et al., 2011; Thi et al., 2011; Meng et al., 2022). Today AMR is a global crisis undermining human and veterinary medicine, food security, and environmental health, demanding accelerated discovery of new antibiotics to contain infections and curb economic losses (Aslam et al., 2018; Lewis, 2020; Meng et al., 2022; Aggarwal et al., 2024; Naghavi et al., 2024). In a report from 2023, the United Nations Environment Programme (UNEP) it was shown that the environment has a key role in AMR development, transmission and spread, and it was stipulated that “Prevention is at the core of the action and environment is a key part of the solution” (UNEP, 2023).

Resistance determinants are part of the natural microbial gene pool, and caves and permafrost environments represent reservoirs where ancient resistance mechanisms can be preserved and studied. This phenomenon often correlates with the metabolic diversity of microbial strains from extreme environments (Perkins and Nicholson, 2008; Allen et al., 2010), therefore, studies of extremophiles from environments with limited anthropogenic influences, including polar and high-altitude habitats, could bring important insights into both AMR evolution and antibiotic discovery (Hemala et al., 2014; Nunez-Montero and Barrientos, 2018; Willms et al., 2019; Kralova et al., 2021; Song et al., 2021; Kim et al., 2022). Recent studies show that cold-environment microbes from Arctic and Antarctic regions (O’Brien et al., 2004; Mogrovejo et al., 2020; Kralova et al., 2021), polar marine ecosystems (Lo Giudice et al., 2007; Tam et al., 2015; Belov et al., 2020; Morozova et al., 2022), and non-Polar high-altitude sites (Ali et al., 2021) harbor unique resistance profiles and are able to produce biomolecules with unique structures and activities, including antimicrobial agents effective against pathogens and other microbes (Hemala et al., 2014; Mogrovejo et al., 2020). Isolated bacteria from Arctic and Antarctic soils and sediments (Nedialkova and Naidenova, 2005; Gesheva, 2010; Shekh et al., 2011; Wong et al., 2011; Mogrovejo et al., 2020; Lee et al., 2012a, 2012b), lakes and benthic mats (Biondi et al., 2008; Rojas et al., 2009; Wietz et al., 2012; Yuan et al., 2014; Lo Giudice et al., 2007a) belong to diverse taxa such as Actinomycetota and Pseudomonadota, and have demonstrated activity against multi-drug resistant (MDR) pathogens (Lo Giudice et al., 2007; Ali et al., 2021). Recent studies indicated that the ice cave environments, with their distinct conditions of low temperature and oligotrophy, host bacterial strains producing antimicrobial and anticancer compounds (Ambrozic Avgustin et al., 2019; Jaroszewicz et al., 2021; Paun et al., 2021; Kosznik-Kwasnicka et al., 2022; Zada et al., 2022).

In the case of Scarisoara Ice Cave, harboring the largest and oldest underground ice block (Holmlund et al., 2005; Persoiu and Pazdur, 2010; Persoiu et al., 2017), recent data revealed preservation of viable bacterial communities in a chronosequence of perennial ice dating back up to 13,000 years (Paun et al., 2019). The cave ice microbiome harbored culturable strains showing a distinct antibiotic resistance profile and various enzymatic and antimicrobial activities (Paun et al., 2021). Such findings underscore the untapped potential of these extreme environments for discovering new bioactive compounds with medical applications.

The genus *Psychrobacter* (family *Moraxellaceae*, class *Gammaproteobacteria*) was described in 1986 with *P. immobilis* as the type species (Juni and Heym, 1986; Juni, 2005). Genomic studies suggest evolutionary adaptation to cold environments from mesophilic ancestors (Bakermans, 2018; Welter et al., 2021). *Psychrobacter* species have a widespread distribution, found primarily in cold and saline habitats (Rodrigues et al., 2009; Lasa and Romalde, 2017; Kampfer et al., 2020), with 44 of 52 identified species validated (Parte et al., 2020; Schoch et al., 2020). These Gram-variable, non-motile coccobacilli form cream to orange colonies and thrive at low temperatures, however tolerating up to 35–37 °C and varying salinities (Bowman et al., 1997; Juni, 2005; Bowman, 2006; Welter et al., 2021). They are strictly aerobic, catalase- and oxidase-positive, and utilize amino and organic acids as carbon sources while displaying limited biochemical versatility (Denner et al., 2001; Bowman, 2006; Gheorghita et al., 2021). Some species, such as *P. sanguinis*, *P. arenosus*, *P. faecalis, P. pulmonis* and *P. phenylpyruvicus* are opportunistic human pathogens (Deschaght et al., 2012; Wirth et al., 2012; Caspar et al., 2013; Yang, 2014; Bonwitt et al., 2018; Ioannou et al., 2025), while others, such as *P. immobilis* or *P. glacincola*, were found to affect fish species (Hisar et al., 2002; Deschaght et al., 2012; El-Sayed et al., 2023). Recent data show the pathogenic potential of *Psychrobacter* species, being rarely reported in human clinical samples (Ioannou et al., 2025). The genus holds biotechnological potential due to cold-active enzymes, carbonic anhydrases for bioremediation, and pathways for terpenoid biosynthesis and benzoate degradation, alongside genes for mercury detoxification and toxin resistance (Bull et al., 2000; Rothschild and Mancinelli, 2001; Bowman, 2006; Lee et al., 2016; Li et al., 2016; Lasa and Romalde, 2017; Staloch et al., 2022). The antibiotic resistance profiles of *Psychrobacter* species remain largely underexplored (Romanenko et al., 2002; Bowman, 2006; Petrova et al., 2009; Bakermans, 2018).

In this context, the current study reports the isolation and functional characterization of a novel *Psychrobacter* strain from 5,000 year-old ice in Scarisoara Ice Cave (Romania) belonging to *P. cryohalolentis* species, with emphasis on its antibiotic resistance profile, antimicrobial potential, and enzymatic activities, integrated with putative determinants revealed by whole-genome sequence analysis. To our knowledge, this is the first genome sequence analysis of *Psychrobacter* species isolated from an ice cave, and first report on ancient microbial resistomes from this habitat.

## 2 Materials and methods

### 2.1 Bacterial strain isolation, identification and characterization

The *Psychrobacter SC65A.3* bacterial strain was isolated from the 5,000 years old ice layer, part of the 25.33-meters ice core from Scarisoara Ice Cave, Romania, at 4 °C, on R2B medium (Paun et al., 2019, 2021).

The growth temperature range was assessed on Tryptic Soy Agar (TSA) medium by incubating the strain at various temperatures in the 4 °C-37 °C interval for 14 days, as described by Paun et al. (2021). The growth salinity range was evaluated in Luria Broth (LB) medium at 15 °C under agitation, using different concentrations of NaCl (0–4 M) and MgCl₂ (0–4 M).

The genomic DNA was isolated using a DNeasy Blood&Tissue Kit (Qiagen, USA), and the strain identification was performed by 16S rRNA gene sequencing, as previously described (Hillebrand-Voiculescu et al., 2013; Paun et al., 2021). The partial 16S rRNA gene sequence of this ice bacterial isolated strain was deposited in GenBank database under accession number MN577402.

Antimicrobial susceptibility testing was carried out by the Kirby-Bauer standard method (Bauer et al., 1966; Hudzicki, 2009; Matuschek et al., 2014) against 28 antibiotics belonging to 17 classes, on Muller-Hinton agar (MH). For the *Psychrobacter SC65A.3* bacterial strain, the incubation has been performed at 15 °C for 48 h, while the *Escherichia coli* ATCC 25922 (BioMérieux, France) and *Staphylococcus aureus subsp. aureus Rosenbach* ATCC 25923 (BioMérieux, France) used as control strains, at 37 °C for 24 h. The MH-agar medium contained 2 g/L meat extract (Scarlab, Spain), 1.5 g/L starch (Merck, Germany), 17.5 g/L casamino acids (VWR, USA), and 17 g/L bacteriological agar (300–2500 ppm calcium, 50–1000 ppm magnesium) (Scarlab, Spain). The interpretation was stratified as follows: (i) because no species-specific breakpoints are available for *Psychrobacter*, zone diameters (mm) for strain SC65A.3 were classified as susceptible or resistant using EUCAST 2020 (v10.0) (The European Committee on Antimicrobial Susceptibility Testing, 2020) and CLSI 2021 (Humphries et al., 2021) breakpoints established for *Acinetobacter* spp., *Moraxella catarrhalis* and *Pseudomonas* spp., which represent the closest taxonomic relatives of the Scarisoara isolate; (ii) for antibiotics lacking breakpoints in these genera, we applied CLSI 2021 breakpoints defined for the reference *Escherichia coli* and *Staphylococcus aureus* strains; (iii) for antibiotics without CLSI/EUCAST breakpoints, interpretation thresholds were taken from published studies, namely novobiocin (R < 16 mm) (Harrington and Gaydos, 1984), lincomycin (R < 6 mm) (Abdul-Jabbar et al., 2022), metronidazole (Hazim et al., 2020), and spectinomycin (R < 14 mm) (Martins et al., 2022).

The antimicrobial activity assays were conducted using a modified diffusion method (Paun et al., 2021), where SC65A.3 extracts (5 μL) were spotted on MH agar medium immediately after pathogen plating, and antimicrobial activity was determined after 18 h at 37 °C. The assay was performed against two reference strains (*Staphylococcus aureus subsp. aureus Rosenbach*) ATCC 25923 (BioMérieux, France) and *Escherichia coli* ATCC 25922 (BioMérieux, France), and 20 clinical isolated pathogens from the Microbial Collection of the Research Institute of The University of Bucharest. The presence of antimicrobial activity was assessed based on the presence or absence of an inhibition zone formed.

The biochemical characterization of the cave isolate SC65A.3 was done using API ZYM and API 20NE strips (BioMérieux, France), at 15 °C incubation temperature.

The enzymatic evaluation of API ZYM was done based on the number of nmol of hydrolyzed substrate as (0): no activity, (1 and 2): low activity (5 nmol and 10 nmol, respectively), (3): moderate activity (20 nmol), (4 and 5): high activity (30 nmol and ≥ 40 nmol, respectively) (Nowak and Piotrowska, 2012; Paun et al., 2021).

### 2.2 Whole genome sequencing (WGS)

Whole-genome sequencing (WGS) and analysis were carried out at Macrogen (South Korea). After 3 days of incubation at 15 °C with agitation (160 rpm), bacterial cells were harvested by centrifugation (30 min, 7,500 rpm) and genomic DNA was extracted as described by Paun et al. (2021). A volume of 300 μL DNA (100 ng/μL) was used for WGS.

*De novo* genome sequencing of the complete genome involved PacBio RSII library construction (20 kb insert size), PacBio RSII SMRT cell sequencing (0.7 GB data per cell), Illumina DNA PCR-free library preparation (350 bp insert size), and Illumina NovaSeq 2 × 150 bp sequencing (6 GB data per sample).

PacBio long reads were assembled with HGAP3, and Illumina reads were subsequently used with Pilon (Walker et al., 2014) to improve sequence accuracy. Subreads were mapped to the assembled contigs to generate a consensus sequence with coverage depth. Following complete genome assembly, BLAST (Altschul et al., 1990) was employed for taxonomic assignment, and the genome was analyzed to identify protein-coding sequences, tRNA genes, and rRNA genes. Functional annotation was performed using the EggNOG database (Huerta-Cepas et al., 2016).

Comparative genomic analysis was performed using CJ Bioscience’s online Average Nucleotide Identity (ANI) calculator^1^ (Yoon et al., 2017) to compare the genome of *Psychrobacter* SC65.3 with those of 45 other *Psychrobacter* species. Genomic data for all comparisons were obtained from the NCBI Prokaryotic Genome Annotation Pipeline (PGAP) (Tatusova et al., 2016; Haft et al., 2018; Li et al., 2021).

Heatmap gene similarity matrices were obtained by importing spreadsheets from Microsoft Excel (Microsoft Corporation, Excel, 2024) into Python 3.11 (Python Software Foundation, 2023) using the pandas 2.2.2 library. Non-numeric entries (e.g., “no similarity”) were converted to zero to allow numerical analysis. Hierarchical clustering and heatmap visualization were performed with seaborn 0.13.2 (clustermap function) and matplotlib 3.9.0. Clustering was computed using the SciPy 1.13.1 hierarchical clustering module with Euclidean distance and average linkage.

The circular representation of the genome was generated using the web-based platform Proksee (Grant et al., 2023). In our workflow, the complete genome sequence was provided in FASTA format, annotations were imported in GFF3 format, and circular rings were set up to display GC content, GC skew, CDS, rRNA, tRNA, CRISPR loci, antibiotic resistance genes (CARD), and other relevant genomic features. The resulting figure was exported in SVG format for graphical editing.

The complete genome sequence of the Scarisoara isolate *Psychrobacter SC65A.3* was deposited in GenBank database under accession number CP106752.1, with NCBI RefSeq assembly number GCF_025642195.1.

### 2.3 Phylogenetic analyses

Phylogenetic analyses of 16S rRNA and *gyr*B sequences using maximum likelihood parsimony, curation, and rendering were performed using the www.phylogeny.fr platform (Dereeper et al., 2008). Sequence alignment was carried out using ClustalW (Thompson et al., 1994). Taxonomic classification of microbial genomes was performed using GTDB-Tk (v2.3.2) within the KBase platform^2^ using the standard workflow (Arkin et al., 2018; Chaumeil et al., 2020). Input genomes were analyzed against the Genome Taxonomy Database (GTDB; release R214) (Parks et al., 2018). The output included comprehensive taxonomic assignments from domain to species level, estimates of genome completeness and contamination, and average nucleotide identity (ANI) values, leading to a phylogenetically consistent and reproducible classification according to the GTDB framework. A compact phylogenetic tree was generated using the Insert Set of Genomes Into SpeciesTree (v2.2.0) tool in KBase (Arkin et al., 2018). The online platform tool iTOL (Interactive Tree of Life) was employed for the visualization, annotation, and refinement of phylogenetic trees generated through maximum-likelihood (ML) analyses (Letunic and Bork, 2024).

## 3 Results

### 3.1 Isolation and identification of *Psychrobacter* SC65A.3

Cultivation on R2A medium at 4 °C of the 5,335 ± 54 years old ice sample collected from a 1,706–1,716 cm-deep ice core of Scarisoara Ice Cave led to growth of an orange/pink-pigmented colony that was selected and further purified (Supplementary Figure S1). This bacterial strain (SC65A.3) could grow on R2A, TSA, MH and LB media. On TSA, the cave isolate grew could grow at temperatures between 4 °C and 15 °C, consistent with its classification as a psychrophilic bacterium (Morita, 1975). When cultivated at 15 °C on LB over 7 days, it tolerated salinities ranging up to 1.9 M NaCl and 0.9 M MgCl_2_, consistent with the characteristics of a moderate halophilic extremophile (Ventosa et al., 1998).

BLAST analysis of the SC65A.3 16S rRNA amplicon showed 97% identity with that of *Psychrobacter glaciei* strain BIc20019 [NR_148850.1] isolated from an Arctic ice core (Zeng et al., 2016). The assignment of the ice cave strain SC65A.3 to the *Psychrobacter* genus was confirmed through phylogenetic analysis of 16S rRNA gene sequences from 43 *Psychrobacter* species, showing that SC65A.3 clustered with 13 other psychrophilic and psychrotrophic members of the genus (Supplementary Figure S2).

### 3.2 Functional characterization of *Psychrobacter* SC65A.3

To evaluate the potential of *Psychrobacter* SC65A.3 new isolate for producing enhanced biotechnological and medical compounds, we determined various enzymatic functions, the antibiotic resistance profile and putative antimicrobial activities of this psychrophilic bacterium preserved for over 5,000 years in ice deposits of Scarisoara Ice Cave.

Screening of enzymatic activities of SC65A.3 using API ZYM system (Gruner et al., 1992) revealed notable production of lipase (C14), alkaline phosphatase, esterase (C 4), esterase lipase (C 8), and naphthol-AS-BI-phosphohydrolase (Table 1). A low enzymatic activity was recorded for valine arylamidase, cystine arylamidase, trypsin, *α*-chymotrypsin, α-glucosidase, and n-acetyl-*β*-glucosaminidase, while no activity was observed for α-galactosidase, β-galactosidase, β-glucuronidase, β-glucosidase, α-mannosidase, and α-fucosidase in this cave-derived psychrophilic strain (Table 1).

**Table 1:** Enzymatic profile of *Psychrobacter* SC65A.3 using API ZYM and API 20NE systems.

*Test API ZYM*		*Test API 20NE*		
		Test	Reaction/Enzyme	Results
**Enzymatic activity**	**Results**	***NO3***	Reduction of nitrates to nitrites	+
Alkaline phosphatase	**3**		Reduction of nitrites to nitrogen	−
Esterase (C 4)	**3**	***TRP***	Indole production	−
Esterase Lipase (C 8)	**3**	***GLU***	Fermentation D-glucose	−
Lipase (C 14)	**4**	***ADH***	Arginine dihydrolase	−
Leucine arylamidase	**3**	***URE***	Urease	+
Valine arylamidase	**2**	***ESC***	hydrolysis (β-glucosidase) (esculin)	+
Cystine arylamidase	**2**	***GEL***	hydrolysis (protease)	−
Trypsin	**1**	***PNPG***	β-Galactosidase	−
α-chymotrypsin	**1**	***GLU***	Assimilation D-glucose	−
Acid phosphatase	**0**	***ARA***	Assimilation L-arabinose	−
Naphthol-AS-BI-phosphohydrolase	**3**	***MNE***	Assimilation D-mannose	−
α-galactosidase	**0**	***MAN***	Assimilation D-mannitol	−
β-galactosidase	**0**	***NAG***	Assimilation N-acetyl-glucosamine	−
β-glucuronidase	**0**	***MAL***	Assimilation D-maltose	−
α-glucosidase	**1**	***GNT***	Assimilation potassium gluconate	−
β-glucosidase	**0**	***CAP***	Assimilation capric acid	−
N-acetyl-β-glucosaminidase	**1**	***ADI***	Assimilation adipic acid	−
α-mannosidase	**0**	***MLT***	Assimilation malic acid	−
α-fucosidase	**0**	***CIT***	Assimilation trisodium citrate	+
		***PAC***	Assimilation phenylacetic acid	−

Color intensity corresponds to the activity (see Methods). No significance of the bold values.

Moreover, the substrate utilization profile of this ice cave isolate, as assessed by the API 20NE system, indicated the presence of nitrate reduction, urea hydrolysis, citrate assimilation and esculin hydrolysis (β-glucosidase) enzymatic activities (Table 1).

Investigation of the antibiotic resistance of *Psychrobacter* SC65A.3 to 28 antibiotics of broad spectrum, narrow spectrum and anaerobe-specific covering 17 classes (Supplementary Figure S3) revealed resistance of this strain to narrow spectrum antibiotics (i.e., clindamycin, lincomycin, vancomycin), to metronidazole, and to 6 out of the 21 broad-spectrum antibiotics (Table 2).

**Table 2:** Antimicrobial susceptibility profiles of *Psychrobacter* SC65A.3 and the reference strains *Staphylococcus aureus* subsp. Rosenbach ATCC 25923 and *Escherichia coli ATCC* 25922.

Antimicrobial spectrum	Antimicrobial class	Antibiotics (code/μg)	SC65A.3	*S. aureus*	*E. coli*
			AST profile (diameter—mm)		
*Broad spectrum*	Penicillin	Ampicillin (AMP/ 25 μg)	S (43)	S (34)	**R (13)**
		Carbenicillin (CAR/100 μg)	S (47)	S (40)	**R (14)**
	Cephalosporins	Ceftazidime (CAZ/30 μg)	**R (18)**	**R (12)**	**R (19)**
		Cefixime (CFM/5 μg)	S (25)	**R (10)**	S (17)
		Cefotaxime (CTX/30 μg)	S (30)	S (26)	S (26)
		Cefpirome (CPO/30 μg)	S (25)	**R (16)**	S (23)
		Cephalothin (KF/30 μg)	**R (6)**	S (35)	**R (6)**
		Cefpodoxime (CPD/10 μg)	S (33)	S (27)	S (22)
	Carbapenems	Imipenem (IPM/10 μg)	S (65)	S (40)	S (35)
	Fluoroquinolones	Ciprofloxacin (CIP/10 μg)	**R (30)**	**R (21)**	S (36)
		Nalidixic acid (NA/30 μg)	S (56)	**R (8)**	S (30)
	Aminoglycosides	Gentamicin (CN/30 μg)	S (29)	S (19)	S (21)
		Spectinomycin (SH/25 μg)	**R (13)**	**R (8)**	**R (14)**
		Streptomycin (S/25 μg)	S (24)	**R (14)**	S (21)
	Coumarin glycosides	Novobiocin (NV/6 μg)	S (26)	**R (19)**	**R (6)**
	Chloramphenicol	Chloramphenicol (C/30 μg)	S (45)	**R (20)**	S (26)
	Nitrofurantoin	Nitrofurantoin (F/100 μg)	S (17)	S (14)	S (25)
	Rifampin	Rifampicin (RD/5 μg)	**R (37)**	S (26)	**R (20)**
	Sulfonamide compounds S3	Sulfonamide compounds (S3/300 μg)	S (47)	**R (6)**	S (26)
	Tetracyclines	Tetracycline (TE/30 μg)	S (39)	S (25)	S (24)
	Trimethoprim/Sulfonamides	Trimethoprim (W/5 μg)	**R (6)**	S (17)	S (23)
*Narrow spectrum (G+)*	Macrolides	Clarithromycin (CLR/15 μg)	S (26)	**R (20)**	**R (18)**
		Erythromycin (E/15 μg)	S (21)	S (21)	**R (12)**
	Lincosamide	Clindamycin (DA/2 μg)	**R (6)**	S (22)	**R (6)**
		Lincomycin (MY/15 μg)	**R (6)**	S (22)	**R (6)**
	Fatty Acyls	Mupirocin (MUP/5 μg)	S (35)	**R (23)**	**R (6)**
	Glycopeptides	Vancomycin (VA/30 μg)	**R (14)**	**R (13)**	**R (6)**
*Anaerobes*	Metronidazole	Metronidazole (MTZ/5 μg)	**R (6)**	**R (6)**	**R (6)**

Resistance (R) and susceptibility (S) categories were assigned from inhibition zone diameters (mm) according to EUCAST breakpoint Table v10.0 (2020), CLSI 2021, and published literature (see Methods). Resistance (bold).

Considering the observed resistance to 10 out of 28 tested antibiotics belonging to 8 classes, this ice cave strain exhibited a multidrug-resistant (MDR) phenotype (Table 2), consistent with the ECDC/CDC consensus definitions considering the MDR phenotypes as non-susceptible to at least one agent from three or more antimicrobial categories (Magiorakos et al., 2012). However, these data must be interpreted with caution. First, no species-specific clinical breakpoints are available for *Psychrobacter*, so the R/S categories were inferred using breakpoints established for related species or from literature-derived cut-offs, rather than from dedicated guidelines for this taxon. Second, given the particular origin of this strain (an ice cave), standard MH agar and conventional incubation conditions may not be optimal for accurately revealing its susceptibility profile (Gattinger et al., 2023). It is also known that elevated cation concentrations can modulate apparent antibiotic resistance by stabilizing biofilm structure, influencing efflux pump activity, or acting as an essential cofactor that alters the activity of certain antibiotics such as daptomycin (Lee et al., 2019; Nechifor et al., 2024; Li et al., 2025). Therefore, the corresponding calcium (5.1–42.5 μg/mL) and magnesium (0.85–17 μg/mL) concentrations in the MH medium used for the SC65A.3 assays may have an impact on the resistance phenotype of the cave strain. In addition, antibiotic susceptibility in cold-adapted bacteria is temperature-dependent, which could bias the quantitative measurements for psychrophilic or psychrotolerant isolates (Gattinger et al., 2023).

The antimicrobial activity of SC65A.3 tested against 20 clinical bacterial strains and 2 ATCC reference strains (Supplementary Figure S4) indicated that this cave isolate inhibited both *S. aureus* and *E. coli* ATCC strains, as well as 12 of the 20 clinical pathogens tested (Table 3). Notably, SC65A.3 showed an antimicrobial activity against several Gram-negative pathogens, including *Enterobacter* spp. (2 strains), *Pseudomonas aeruginosa* (3 strains), *Klebsiella pneumoniae* (3 strains), and *E. coli* ATCC 25922 (Table 3). Meanwhile, no inhibitory effect against *Acinetobacter baumannii* clinical isolates, *Enterobacter asburiae* 19,069 ONE1, *Klebsiella pneumoniae* 19,047 ENE4, and two methicillin-resistant *Staphylococcus aureus* (MRSA) strains was observed (Table 3).

**Table 3:** Antimicrobial activity of *Psychrobacter* SC65A.3.

Test pathogen	Antimicrobial activity of *Psychrobacter sp.* SC65A.3 (+/−)
*Gram positive pathogenic strains*	
*Staphylococcus aureus* ATCC 25923	**+**
MRSA 19081 F1	**+**
MRSA 19081 S1	−
MRSA 388	−
*Enterococcus faecium* 19,040 E1	**+**
*Enterococcus faecium* 19,040 E2	**+**
*Enterococcus faecium* 19,040 E3	**+**
*Gram negative pathogenic strains*	
*Escherichia coli* ATCC 25922	**+**
*Enterobacter asburiae* 19,069 ONE1	−
*Enterobacter cloacae* 19,069 ONE2	**+**
*Enterobacter cloacae* 19,069 ONE3	**+**
*Pseudomonas aeruginosa* CN11	**+**
*Pseudomonas aeruginosa* 19,053 CNE5	**+**
*Pseudomonas aeruginosa* 19,053 CNE6	**+**
*Klebsiella pneumoniae* 8	−
*Klebsiella pneumoniae* 19,094 CK1	**+**
*Klebsiella pneumoniae* 19,094 CK2	**+**
*Klebsiella pneumoniae* 19,094 CK3	**+**
*Acinetobacter baumannii* 19,047 ENE4	−
*Acinetobacter baumannii* 19,047 CNE5	−
*Acinetobacter baumannii* 19,047 CNE3	−
*Acinetobacter baumannii* 18,032 C3	−
Total	14

Presence (+) and absence (−) of inhibitory growth effect on Gram-positive and Gram-negative pathogenic strains of *S. aureus* and *E. coli* ATCC collection strains, and clinical strains of genera MRSA, *Enterococcus, Enterobacter, Pseudomonas, Klebsiella*, and *Acinetobacter.*

#### 3.2.1 Whole genome sequencing and analyses

Based on the functional properties of Scarisoara strain SC65A.3, the whole genome was sequenced for identifying structural elements associated with the natural antibiotic resistance of this cave strain entrapped in ice for the last 5 millennia and for future studies investigating novel cold-active enzymes and bioactive molecules, also highlighting the putative thermal-adaptation gene pool of this psychrophilic ice cave bacterium.

**Figure 1:**
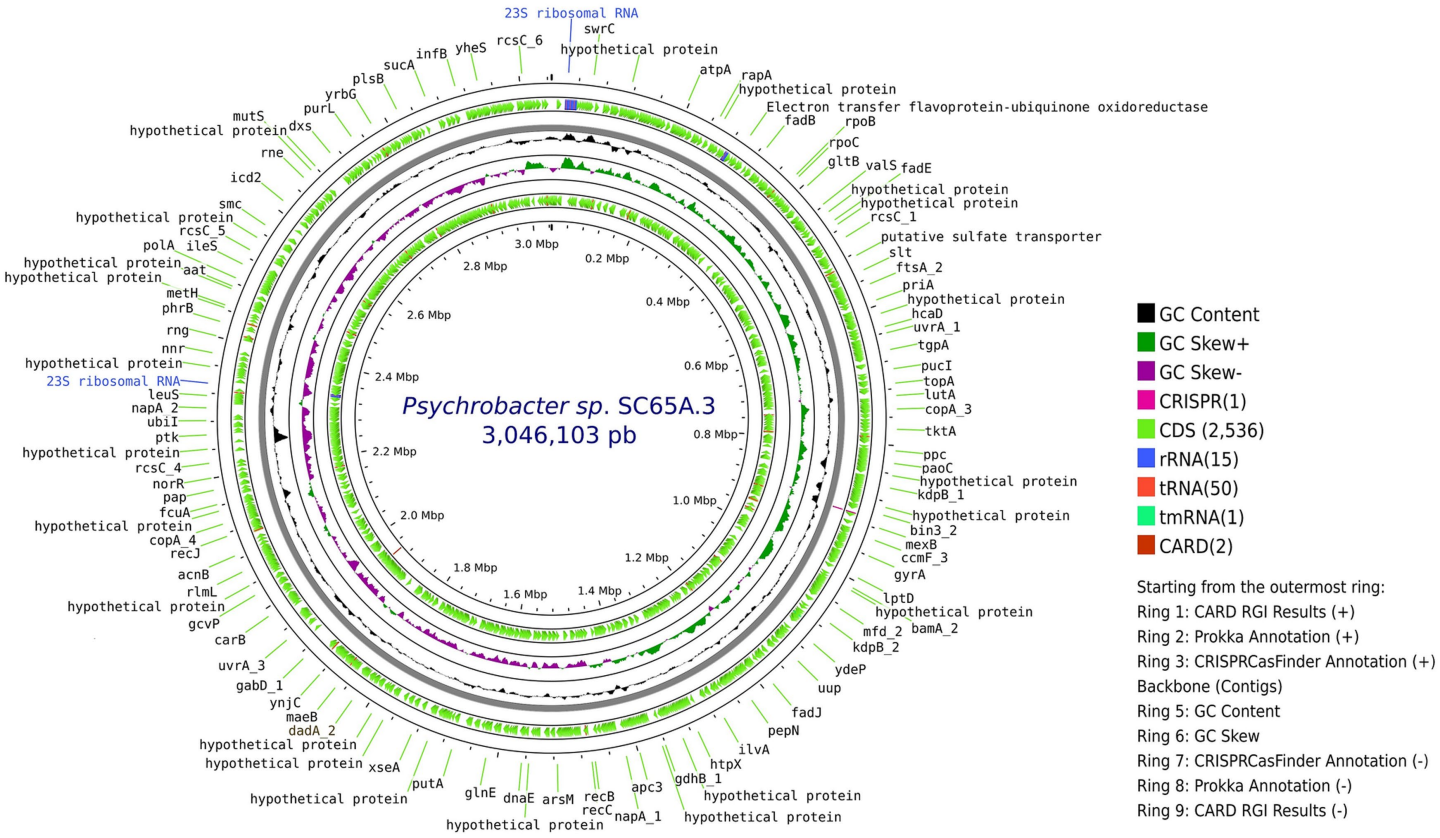
Circular representation of the complete genome of *Psychrobacter* sp. SC65A.3. From the outermost to the innermost rings: (1) CARD RGI resistance gene hits (positive strand), (2) annotated coding sequences (CDS) identified by Prokka, (3) CRISPR loci detected by CRISPRCasFinder, (4) genome backbone (contigs), (5) GC content, (6) GC skew (green = positive, purple = negative), (7) CRISPRCasFinder annotations (negative strand), (8) Prokka CDS annotations (negative strand), and (9) CARD RGI results (negative strand). Gene names indicate key metabolic, regulatory, or structural functions identified along the chromosome.

PacBio sequencing and subreads filtering of Scarisoara strain SC65A.3 genome produced 1,351,448,809 subread bases, with a mean 10,421 subread length and 129,674 subreads with an *N50* value of 14,351 base pairs. *De novo* genome assembly generated a unique circular contig of 3,046,103 bases length (Figure 1), with a GC content of 42.52%. The genome comprised 2,602 CDS, 15 rRNA genes, 50 tRNA genes, and 15 rRNA, with a single CRISPR locus and two predicted antibiotic resistance genes (Figure 1).

BLAST analysis of the complete genome revealed 97% identity with the *Psychrobacter cryohalolentis* strains FDAARGOS_308 [CP022043.2] and K5 [CP000323.1], and with *Psychrobacter* sp. strain G [CP006265.1] (Supplementary Table S1).

**Figure 2:**
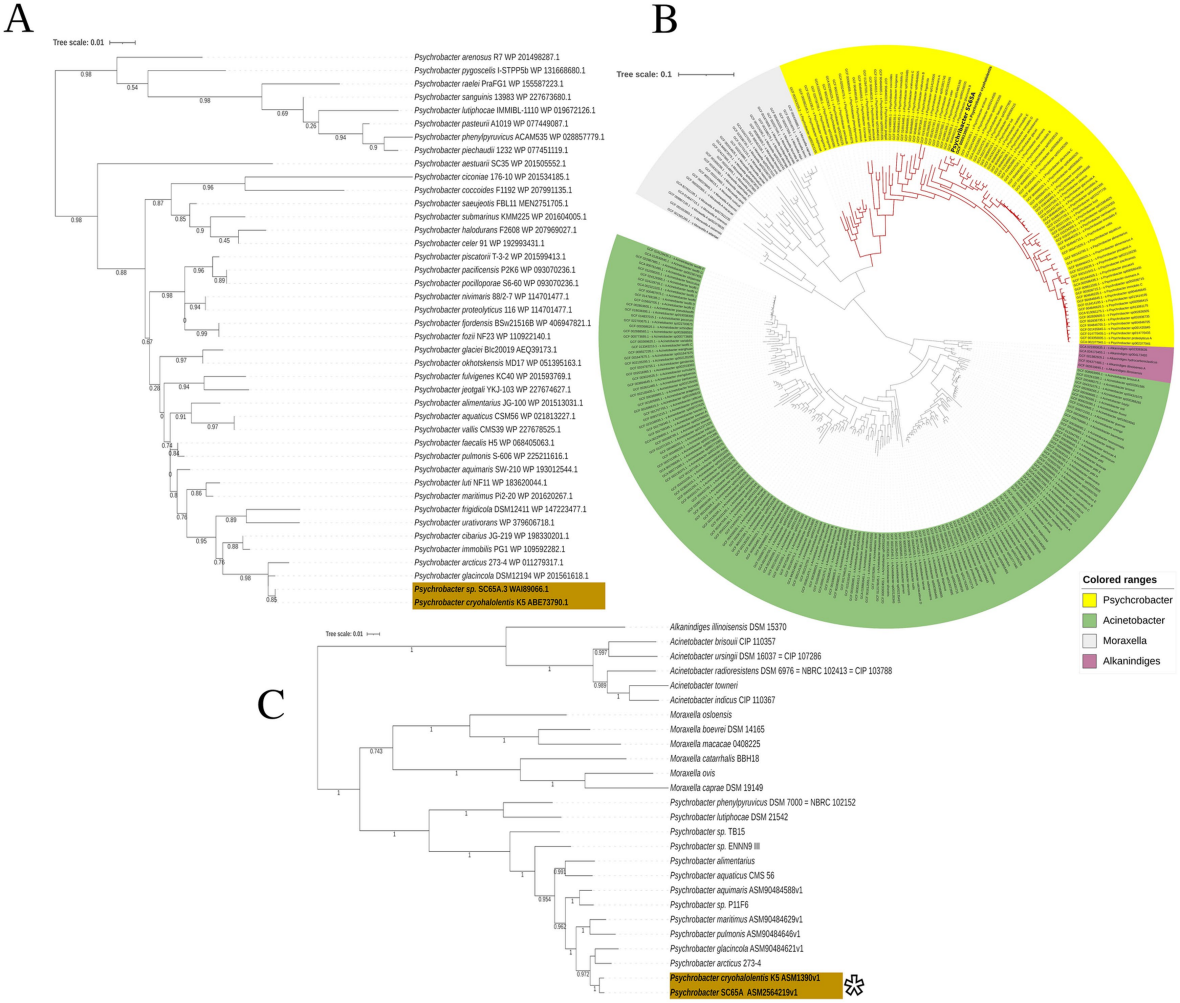
Phylogenetic position of *Psychrobacter* sp. SC65A.3 based on *gyrB* gene and whole-genome sequences. **(A)** Maximum-likelihood tree based on *gyrB* gene sequences showing the phylogenetic position of *Psychrobacter* sp. SC65A.3 among 44 *Psychrobacter* strains retrieved from the NCBI database. **(B)** Maximum-likelihood phylogenomic tree constructed from 332 genomes, including 100 *Psychrobacter* genomes, illustrating the taxonomic relationships within the *Moraxellaceae* family. Colored ranges indicate different genera: *Psychrobacter* (green), *Acinetobacter* (yellow), *Moraxella* (purple), *Alkanindiges* (gray). **(C)** Maximum-likelihood tree based exclusively on *Psychrobacter* genomes, highlighting the clustering of *Psychrobacter* sp. SC65A.3 with its closest relatives, *P. cryohalolentis* K5 and *P. glacincola* DSM 15331. Bootstrap values are indicated at the nodes. (*) ANI value = 96.63%.

Maximum likelihood phylogenetic analysis based on 41 *gyrB* gene sequences (Bakermans et al., 2006) sustained the taxonomic affiliation of SC65A.3 by positioning this ice cave strain within a well-supported clade alongside *Psychrobacter cryohalolentis* K5 [CP000323.1], suggesting a close evolutionary relationship within the genus (Figure 2A). This lineage was also grouped with *P. glacinicola* DSM12194 [WP_201561618] and *P. arcticus* 273–4 [WP_011279317] with high bootstrap support (~0.95), while showing more distant evolutionary relationships to other *Psychrobacter* clustered lineages of *P. phenylpyruvicus*, *P. pacificensis*, and *P. piechaudii* (Figure 2A). These findings indicate that SC65A.3 belongs to a cold-adapted *Psychrobacter* lineage, in agreement with the phylogenetic pattern observed in the 16S rRNA gene-based analysis (Supplementary Figure S4).

A phylogenomic analysis of whole genomes based on 322 genomes, among which 100 from *Psychrobacter* genus (Figure 2B), revealed that *Psychrobacter* sp. SC65A.3 clustered firmly within the monophyletic clade corresponding to genus *Psychrobacter*, that is clearly separated from the related genera *Acinetobacter, Moraxella,* and *Alkanindiges* (Figure 2C). Within this group, SC65A.3 was positioned in a well-supported subclade together with *Psychrobacter cryohalolentis*, as also observed by the *gyr*B phylogenetic analysis (Figure 2A), a species known for its adaptations to permafrost environments and cryogenic conditions (Bakermans et al., 2006). The close phylogenetic relationship between SC65A.3 and *P. cryohalolentis* (Figure 2C) suggests a high degree of genomic similarity and a possible common origin in cold or marine ecosystems, in line with the general characteristics of the genus *Psychrobacter* adapted to Polar and cold marine environments. In addition to the phylogenomic profile, the 96.63% average nucleotide identity (ANI) across all shared orthologous genes between *Psychrobacter* sp. SC65A.3 and the three *Psychrobacter cryohalolentis* genomes (Figure 2C) supports the assignment of the cave strain to this species.

#### 3.2.2 *Psychrobacter* SC65A.3 genome annotation

Sequence homology analysis of the assembled *Psychrobacter* SC65A.3 genome indicated a total of 2,602 genes of which 2,536 protein-coding sequences (CDSs) and tRNA and rRNA genes (Supplementary Table S1, Figure 2A). Functional annotation disclosed 2,428 genes assigned to a single EggNOG orthologous group and 56 mapped to multiple groups (Supplementary Table S1).

**Figure 3:**
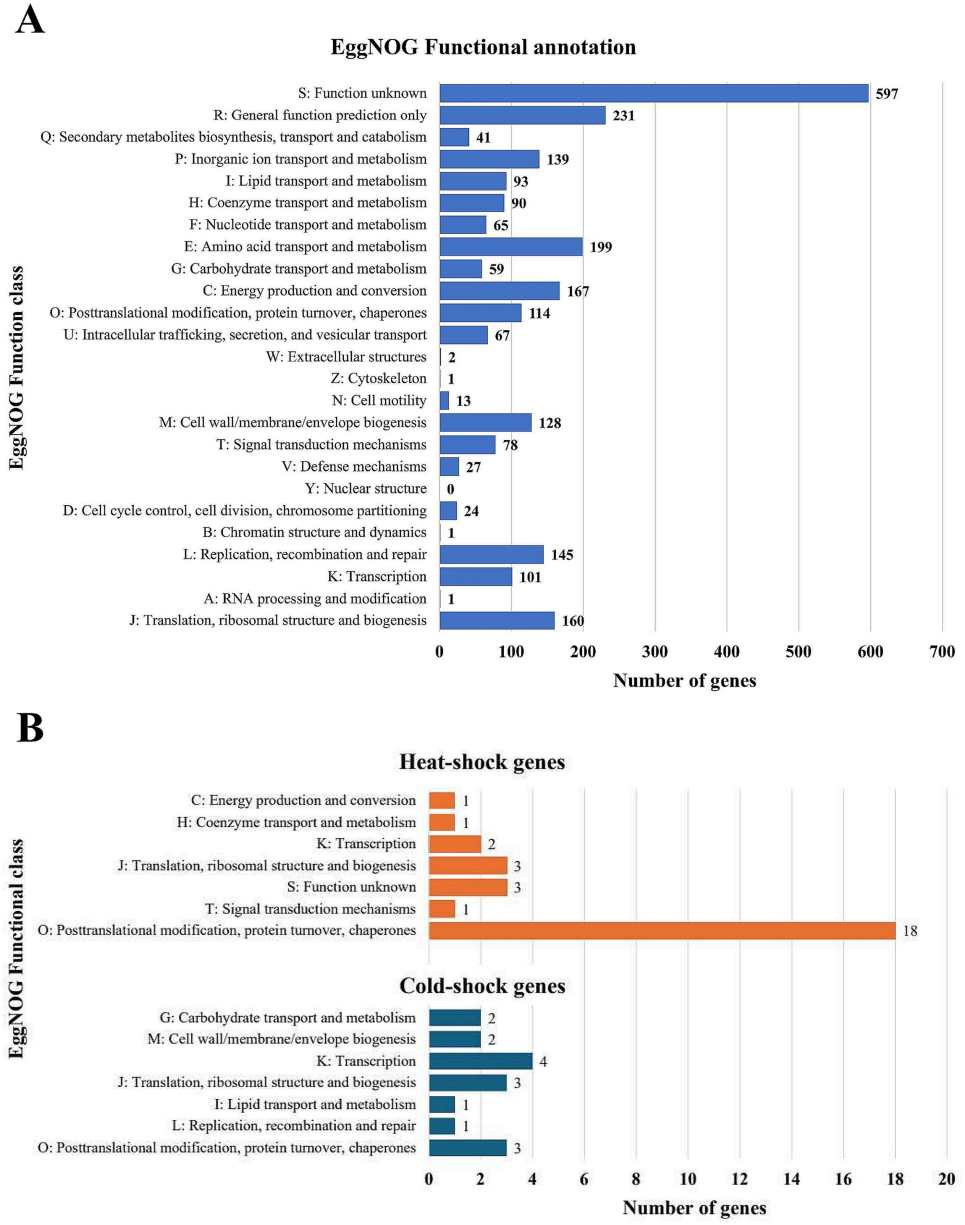
Gene annotation and temperature adaptability gene profile of *Psychrobactcer* SC65A.3. **(A)** EggNOG functional gene annotation and classification; **(B)** Heat-shock (orange) and cold-shock (blue) gene distribution based on EggNOG analysis.

Among the assigned genes, the largest group was associated with general function prediction (231), amino acid transport and metabolism (199), and energy production and conversion (167) (Figure 3A). A considerable number of genes were linked to translation, ribosomal structure, and biogenesis (160), replication, recombination, and repair (145), inorganic ion transport and metabolism (139), and cell wall/membrane/envelope biogenesis (128). Also, more than 100 genes related to posttranslational modification, protein turnover, and chaperone activity and transcription were annotated, while single genes were identified for RNA processing and modification, chromatin structure and dynamics, and cytoskeleton organization, while no genes associated with nuclear structure were detected (Figure 3A). Notably, a substantial number of genes (597) were classified with unknown functions, suggesting untapped potential for discovering novel biological mechanisms in the psychrophilic *Psychrobacter* strain from this underexplored icy habitat (Figure 3A).

#### 3.2.3 Low temperature adaptation genes

Given the psychrophilic nature of this ice-isolated bacterial strain, analysis of the SC65A.3 genome highlighted 45 genes associated with thermal adaptation, among which a large majority (29) involved in heat-shock processes and only 16 in cold-shock responses (Supplementary Table S2; Figure 3B). As expected, most of these genes (21) are chaperones involved in posttranslational modifications and protein turnover, primarily representing heat-shock adaptations (18 genes) (Figure 3B). A restrained number of genes (2–4) associated with transcription and translation processes and ribosomal structure and biogenesis appeared to be involved in both heat-shock and cold-shock processes, while distinct pathways appeared to participate to either cold-shock (6 genes from carbohydrate and lipid transport and metabolism, cell wall biogenesis, and replication, recombination, and repair), or heat-shock (3 genes involved in energy production and conversion, coenzyme transport and metabolism and signal transduction) processes (Figure 3B). Interestingly, three genes classified under EggNOG unknown functions were assigned to heat-shock response, indicating potential unique thermal adaptation mechanisms in this cave ice-retrieved bacterial strain (Figure 3B).

#### 3.2.4 Antibiotic resistance genes (ARGs) in correlation with the phenotypic profile of the *Psychrobacter* SC65A.3 strain

In line with the phenotypic profile of antibiotic resistance of SC65A.3, the genomic DNA coding sequence was analyzed with a focus on genes associated with intrinsic or acquired antibiotic resistance, and with biosynthesis of compounds with antimicrobial activity (Table 4).

**Table 4:** EggNOG *Psychrobacter* SC65A.3 functional annotation for antibiotic resistance genes (ARGs) and biosynthesis of antimicrobial compounds.

EggNOG genes	ARGs (no)	Antimicrobials biosynthesis genes (no)
**J:** Translation, ribosomal structure, biogenesis (160)	*aviRb, fusA, ileS, rng, rplD, rplF, rplV, rpmG, rpsD, rpsE, rpsL, rpsQ, rsmA_1, thrS (14)*	*-*
**K:** Transcription (101)	***rpoB**, rpoC, **merR1_1,** rpoD, **tetC,** acrR, rpoA, **merR1_2,** coaX (9)*	*-*
**L:** Replication, recombination & repair (145)	***parE, parC, gyrA**, mfd, nudC_2, polC, **gyrB** (7)*	*-*
**D:** Cell cycle control, cell division, chromosome partitioning (24)	*crcB, relE (2)*	*-*
**V:** Defense mechanisms (24)	***ampC**, **emrK, macB, mdtA, mdtK**, mepA, **mexA, msbA,** uppP, yojI (10)*	*-*
**T:** Signal transduction mechanisms (78)	*cpxA, cpxR, phoP, walR*	*-*
	*zraR, zraS_1, zraS_2 (7)*	
**M:** Cell wall/membrane/ envelope biogenesis (128)	***ampD,*** ***ftsI,** galU, lpxA, lpxC, lpxD, **macA,** mipA, murA, murG, murU, **oprM,** porB, rsmG, ywnH (15)*	*sunS (1)*
**U:** Intracellular trafficking, secretion, vesicular transport (67)	*pilQ (1)*	*-*
**O:** Posttranslational modification, protein turnover, chaperones (114)	*anmK (1)*	*-*
**C:** Energy production/ conversion (167)	***merA** (1)*	*pikAII (1)*
**G:** Carbohydrate transport/ metabolism (59)	***ampG,** nagZ (2)*	*-*
**E:** Amino acid transport/ metabolism (199)	*aroK, mhqA (2)*	*btrK, rebO_1, rebO_2 (3)*
**F:** Nucleotide transport/ metabolism (65)	*thyA, nudC_1, dut (3)*	*-*
**H:** Coenzyme transport/ metabolism (90)	***dfrA, folP,** ubiA, rsmA_2 (4)*	*-*
**I:** Lipid transport/ metabolism (93)	*clsA_1, clsA_2, cdsA, fabI, clsC, pgsA (6)*	*dpgD (1)*
**P:** Inorganic ion transport/ metabolism (139)	***arsA, arsB, arsC, bcr**, cirA, **emrE, merP**, **mexB**, swrC, yejB, yejE (11)*	*-*
**Q:** Secondary metabolites biosynthesis/ transport/ catabolism (41)	*sttH (1)*	*-*
**R:** General function prediction (231)	***tetA** (1)*	*-*
**S:** Function unknown (597)	*amgK, arsH, **mcr1,** mshB, mupP, pvdQ, rarD_1, rarD_2, rarD_3, yejF (10)*	*bacC, bpoA2, cetB, rdmC, sdhE (5)*
**Total count genes: 2543**	**107**	**11**

Genes relevant to clinical resistance (bold).

From the total of 2,543 genes, 107 were identified as genes associated with AMR (Table 4). Some of them were distributed across a broad range of functional categories within the EggNOG database, including transcription, replication, recombination and repair, as well as signal transduction mechanisms. Other genes were associated with lipid transport and metabolism, coenzyme transport and metabolism, and nucleotide transport and metabolism. A smaller subset of resistance-associated genes was related to cell cycle control, amino acid metabolism, and carbohydrate metabolism. Single genes were also identified within categories related to energy production, secondary metabolite biosynthesis, intracellular trafficking, general function prediction, and posttranslational modification (Table 4).

The genes relevant to clinical resistance (Table 4) are associated with resistance to *β*-lactams (*ampC, ftsI, ampD, ampG*), tetracyclines (*tetA, tetC*), fluoroquinolones (*gyrA, gyrB, parC, parE*), aminoglycosides / multidrug efflux (*mexA, mexB, oprM, emrK, mdtA, mdtK, macB, mepA, msbA, macA, bcr, emrE*), rifampin (*rpoB*), trimethoprim / sulfonamides (*dfrA, folP*), colistin (*mcr1*), resistance to heavy metals (*arsA, arsB, arsC, merA, merP, merR1_1, merR1_2*).

**Figure 4:**
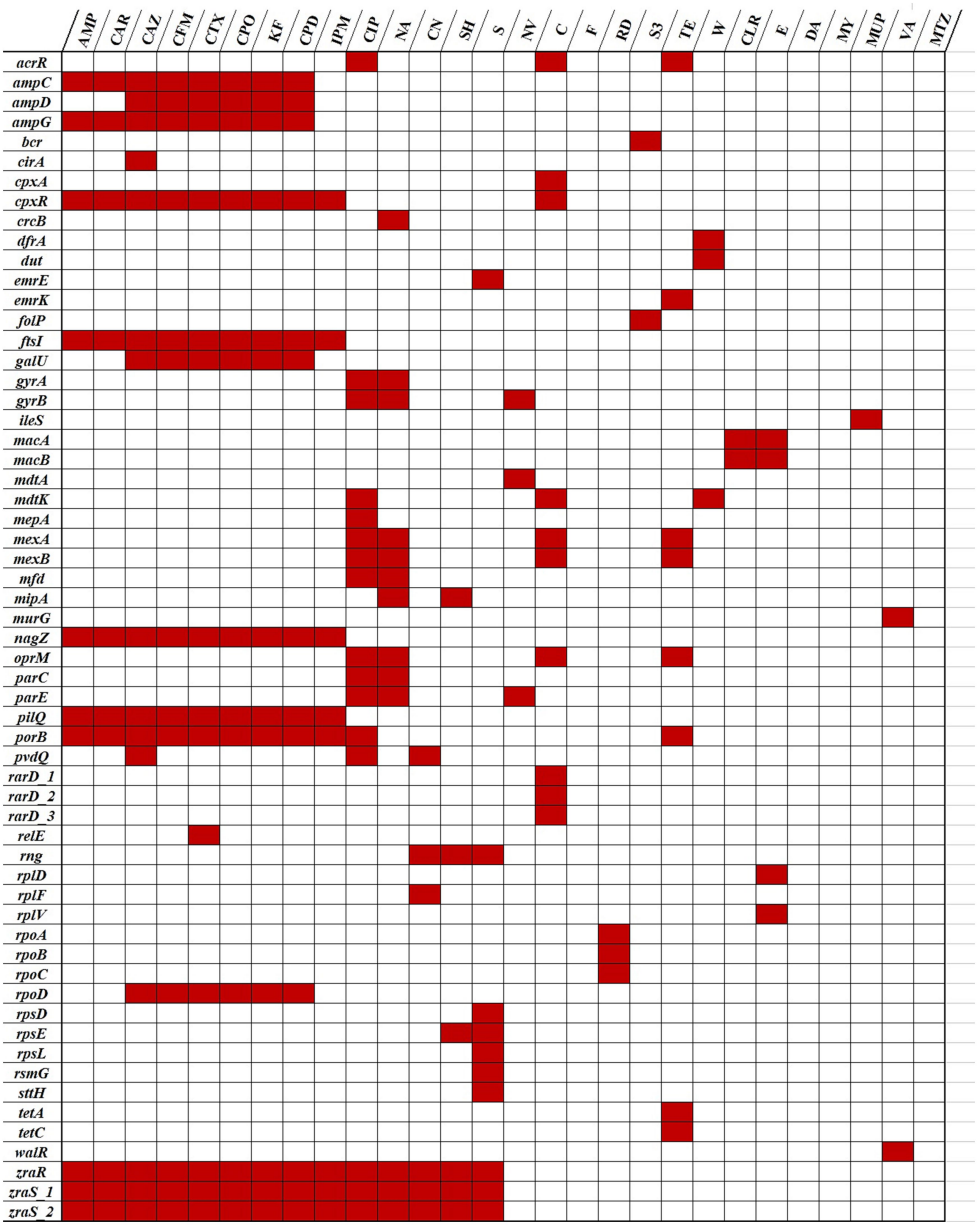
Correlation of the *Psychrobacter* SC65A.3 antibiotic resistance phenotypic and genotypic profiles. (Red blocks): Resistance genes (*y*-axis) matching the phenotypic antibiotic resistance (*x*-axis); (white blocks): no resistance genes detected or antibiotic susceptibility not tested. Data available in Tables 2, 4.

From the 107 ARGs, 44 could be linked to intrinsic or acquired resistance against the antibiotics tested in this study (Figure 4).

The correlation analysis between the phenotypic resistance profile (Table 2) of the SC65A.3 strain and its gene pool related to antibiotic resistance (Table 4) shown that resistance to certain antibiotics was not linked to any specific ARG detected in the genome (Figure 4). However, the functional resistance to these antibiotics could be due to the presence of multidrug efflux genes harbored by this strain (Jian et al., 2021; Gaurav et al., 2023; Elshobary et al., 2025).

#### 3.2.5 Antimicrobial activity-related genes in *Psychrobacter* SC65A.3

Analysis of the SC65A.3 genome for genes potentially involved in antimicrobial activity identified a total of 11 candidates (Table 4). The identified genes are involved in the biosynthesis of sublancin, an S-linked glycopeptide (*sunS*) (Oman et al., 2011), pikromicin (*pikAII*) (Xue et al., 1998) (https://www.uniprot.org/uniprotkb/Q9ZGI4/entry), butirosin (*btrK*) (Popovic et al., 2006), rebeccamycin (*rebO*) (Nishizawa et al., 2005), glycopeptide (*dpgD*) (Chen et al., 2001), bacitracin (*bacC*) (Konz et al., 1997), haloperoxidase (*bpoA2*) (Hecht et al., 1994), cetoniacytone A (*cetB*) (Wu et al., 2009), anthracycline tailoring (*rdmC*) (Jansson et al., 2003), and a succinate dehydrogenase assembly factor (*sdhE*) (McNeil et al., 2012) (Table 4).

## 4 Discussion

### 4.1 Taxonomic position and physiological characteristics of the ice cave *Psychrobacter* SC65A.3 strain

Despite the remarkable ecological and biotechnological potential of psychrophilic and psychrotrophic bacteria, the recovery and characterization of cold-adapted antibiotic producers remain limited as compared to mesophiles (O’Brien et al., 2004; Sanchez et al., 2009; Silva et al., 2018). To advance a comprehensive understanding of microbial life in cold environments, integrated research should focus on mapping their taxonomic and functional diversity, uncovering the mechanisms of cold adaptation, evaluating their roles in biogeochemical cycles and climate feedback processes, and exploring novel microbial taxa and functions with potential applications in biotechnology and medicine.

The novel bacterial strain SC65A.3 isolated from 5,000-year-old ice deposits of Scarisoara Ice Cave represents a polyextremophile classified as psychrophilic, with a growth range of 4–15 °C, and as a moderate halophile, tolerating up to 1.8 M NaCl and 0.9 M MgCl₂. Molecular identification based on 16S rRNA gene homology indicated the closest match (97% identity) with *Psychrobacter glaciei* BIc20019 [NR_148850.1] originating from an Arctic ice core in Svalbard (Zeng et al., 2016).

The genus *Psychrobacter* displays a wide ecological distribution, with species recovered from various habitats, primarily cold and saline environments (Rodrigues et al., 2009; Lasa and Romalde, 2017). According to the NCBI Taxonomy Database (Schoch et al., 2020) and the List of Prokaryotic Names with Standing in Nomenclature (Parte et al., 2020), 52 *Psychrobacter* species have been described to date, of which 44 are taxonomically validated and 43 have documented morphological or functional characterization.

*Psychrobacter* species are able to thrive at low temperatures, being predominantly psychrotrophic or psychrophilic, while some strains can grow at 35–37 °C, with a minimum temperature limit of 10 °C (Vela et al., 2003; Romanenko et al., 2004; Baik et al., 2010; Kampfer et al., 2015, 2020). Additionally, certain species tolerate a broad salinity range, being categorized as polyextremophiles (Juni, 2005; Bowman, 2006; Welter et al., 2021) (Supplementary Table S3). In this respect, SC65A.3 represents a novel psychrophilic and halotolerant *Psychrobacter* strain, consistent with the characteristics of many species inhabiting cold environments.

Most *Psychrobacter* isolates, including the cave strain SC65A.3, can grow in the absence of added salts, whereas some require elevated salinity for optimal growth. For example, *P. cibarius*, isolated from fermented food, grows only within a 2–10 M NaCl range (Jung et al., 2005), and *P. pulmonis*, from lamb lungs, requires 1.1 M NaCl (Vela et al., 2003) (Supplementary Table S3). Most *Psychrobacter* species tolerate NaCl concentrations up to approximately 2 M, comparable to SC65A.3 which grows in media containing up to 1.9 M NaCl (Supplementary Table S3). In contrast, marine species demand higher salinity, such as *P. nivimaris* from the South Atlantic (2.23 M) (Heuchert et al., 2004), *P. submarinus* from Pacific waters (Romanenko et al., 2002; Shivaji et al., 2004; Kaur et al., 2023) and *P. celer* from the South Sea, both tolerating up to 2.7 M NaCl (Yoon et al., 2005c), and *P. saeujeotis* from salted shrimp jeotgal, which grows in up to 3.5 M NaCl (Kim et al., 2025).

Given the cold-loving characteristics of most *Psychrobacter* species, the industrial and biotechnological interest in these bacteria is focused on the discovery of novel cold-active proteins and enzymes with broad applicability (Bull et al., 2000; Rothschild and Mancinelli, 2001; Bowman, 2006). In this respect, the highly active carbonic anhydrase of *Psychrobacter* sp. SHUES1 (Li et al., 2016) is a potential biocatalyst for bioremediation, while other strains harboring two metabolic pathways involved in terpenoids biosynthesis and benzoate degradation (Lee et al., 2016) could be exploited for food and pharmaceutical industries.

Based on ANI values (Supplementary Table S4) and on literature data, 12 different *Psychrobacter* species among which 7 psychrophilic and 5 psychrotrophic were phenotypically compared to the Scarisoara strain, showing a different functional profile with respect to substrate assimilation and enzymatic activities (Supplementary Table S3).

Among the metabolic activities revealed by the API 2ONE and API ZYM biochemical tests, SC65A.3 evidenced a distinct functional profile regarding esculin hydrolysis (absent in all other species), urease activity (present only in *P. muriicola* 2pS), cysteine arylamidases (present only in *P. pulmonis* S-606), and scarcely encountered valine arylamidase activity and trisodium citrate catabolism (Supplementary Table S3). Most reported *Psychrobacter* species are unable to hydrolyze complex substrates, while some are capable of protein hydrolysis, preferentially utilize organic and amino acids as carbon sources, and frequently exhibit lipolytic activity (Denner et al., 2001; Bowman, 2006). The lipase (C14) activity of SC65A.3, also observed in certain psychrophilic *Psychrobacter* species (Supplementary Table S3), was evaluated for potential biotechnological applications (Gheorghita et al., 2021; Paun et al., 2024), given the high hydrolytic capacity of this ice-cave isolate toward long-chain fatty acids.

Overall, the enzymatic profile of this novel ice cave strain indicates a wide-ranging potential for applications in biotechnology, pharmaceuticals, and environmental processes.

### 4.2 Genetic comparison of cold-adapted *Psychrobacter* strains

The current study providing the first whole-genome sequence of a *Psychrobacter* strain isolated from cave ice belonging to *P. cryohalolentis* species revealed a range of distinctive genomic features as compared to other *Psychrobacter* species from cold environments. These unique traits may reflect specific adaptations to the extreme conditions of this subterranean ice habitat.

At the genome assembly level, only 12 *Psychrobacter* species were found to have complete genomes, of which six are psychrophilic strains, similar to SC65A.3 (Supplementary Table S4). Based on Average Nucleotide Identity (ANI) values, the closest genomic identity to SC65A3 was observed with the psychrophilic strains *Psychrobacter* sp. G (96.84%) and *P. cryohalolentis* K5 (96.63%) isolated from Antarctic seawater (Che et al., 2013; Wang et al., 2017) and a Siberian cryopeg (Bakermans et al., 2003, 2006), respectively. Lower ANI values were recorded for the psychrophilic *P. arcticus* 273–4 (88.13%) isolated from Siberian permafrost (Bakermans et al., 2006), and *P. glacincola* DSM12194 (88.06%) originating from Antarctic Sea ice (Bowman et al., 1997). The lowest score (72.3%) was found with the mesophilic *P. phenylpyruvicus* ACAM535 strain (Bovre and Henriksen, 1967; Bowman et al., 1996) (Supplementary Table S4), suggesting that temperature adaptation in *Psychrobacter* is linked to distinct genetic mechanisms.

The genome sizes of analyzed *Psychrobacter* species range from 3,696,886 bp in *P. arenosus* (isolated from marine sediment in the Sea of Japan) (Romanenko et al., 2004) to 2,484,493 bp in *P. ciconiae* (isolated from white storks) (Kampfer et al., 2015), with that of SC65A.3 falling within this range at 3,046,103 bp (Supplementary Table S4). Comparison of the GC content of *Psychrobacter* strain SC65A.3 (42.5%) with 45 other *Psychrobacter* species revealed identical values with 11 strains, including the psychrophilic *Psychrobacter* sp. G (Che et al., 2013; Wang et al., 2017), *P. cryohalolentis* K5 (Bakermans et al., 2003, 2006), *P. glacincola* DSM12194 (Bowman et al., 1997), and *P. nivimaris* 88/2–7, isolated from southern Atlantic seawater (Heuchert et al., 2004), and seven psychrotolerant or mesophilic strains counting the Antarctic *P. luti* NF11 and *P. fozii* NF23 strains (Bozal et al., 2003), *P. fjordensis* BSw21516B retrieved from Arctic seawater (Zeng et al., 2015), *P. saeujeotis* FBL11 (Kim et al., 2025) and *P. jeotgali* YKJ-103 (Yoon et al., 2003) isolated from fermented seafood, alongside *P. pasteurii* A1019 and *P. piechaudii* 1,232 (Hurtado-Ortiz et al., 2017). A relatively higher GC content (49.5%) was observed in the case of the mesophilic strain *P. aestuarii* SC35 originating from tidal flat sediment (Baik et al., 2010) (Supplementary Table S4).

Comparative analysis of *Psychrobacter* genomes showed that the predicted gene count of the Scarisoara SC65A.3 strain is similar to both psychrophilic and psychrotolerant species, despite differing isolation habitats. Its total gene number closely matches psychrophiles like *P. cryohalolentis* K5 (2,615) from Siberian cryopeg (Bakermans et al., 2003, 2006) and *P. submarinus* KMM225 (2,624) from Pacific sea water (Romanenko et al., 2002; Shivaji et al., 2004; Kaur et al., 2023), as well as the mesophilic *P. celer* 91 (2,583) from the Korean South Sea (Yoon et al., 2005c), in contrast with the psychrotolerant *P. piscatorii* T-3-2 (Yumoto et al., 2010) that has a notably higher gene count of 3,049 (Supplementary Table S4).

Genome annotation indicated a relatively high number of rRNA genes (15) in the case of SC65A.3, similar to other seven *Psychrobacter* species including the psychrophilic *P. pacificensis* WSYP01 isolated from 6,000 m depth in the Japan Trench (Maruyama et al., 2000) (Supplementary Table S4), while only two other species showed a higher number (18) of rRNA genes, counting the psychrophilic *P. muriicola* 2pS originating from permafrost (Shcherbakova et al., 2009) and the psychcrotolerant *P. fjordensis* BSw21516B isolated from Arctic seawater (Zeng et al., 2015) (Supplementary Table S4). *Psychrobacter* SC65A.3 genome also contains 50 tRNA genes, matching two other species—the psychrophile *P. pacificensis* WSYP01 (Maruyama et al., 2000) and the mesophile *P. alimentarius* PAMC27889 (Yoon et al., 2005a), as compared to the mesophilic *P. raelei* PraFG1 (57) (Manzulli et al., 2024) (Supplementary Table S4).

**Figure 5:**
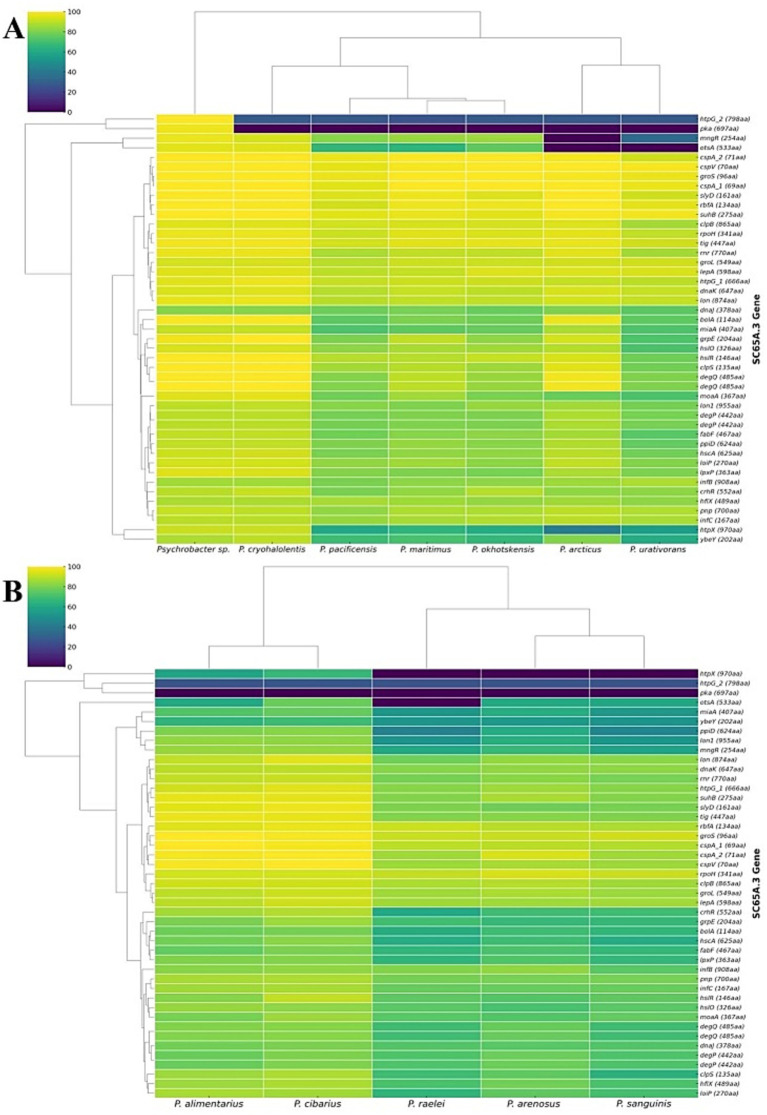
Heatmap distribution of temperature-shock genes in *Psychrobacter* genomes as compared to annotated SC65A.3 genes. **(A)** Psychrophilic *Psychrobacter* species; **(B)** Psychrotrophic *Psychrobacter* species. The annotated heat-shock and cold-shock SC65A.3 genes are indicated in Supplementary Table S2.

The genes set associated with heat-shock and cold-shock adaptation in *Psychrobacter* SC65A.3 (Supplementary Table S2) shows a distinct distribution across other *Psychrobacter* species (Figure 4). Heatmap analysis of these genes compared with those from 7 psychrophilic (Figure 5A) and 5 psychrotrophic strains (Figure 5B) revealed partial interspecies conservation. The highest conservation of thermal-adaptation genes was observed among psychrophilic species with *Psychrobacter* sp. G from Antarctic seawater (Che et al., 2013; Wang et al., 2017), *P. cryohalolentis* from Siberian cryopeg (Bakermans et al., 2003, 2006) and *P. articus* from Siberian permafrost (Bakermans et al., 2006), and to a lesser extent with the marine species *P. maritimus* (Romanenko et al., 2004), *P. okhotskensis* (Yumoto, 2003), and *P. pacificensis* (Maruyama et al., 2000) (Figure 5A). Psychrotolerant species generally showed lower conservation of heat-shock and cold-shock genes as compared to SC65A.3 strain, with a closer genome pool profile in the cases of *P. alimentarius* (Yoon et al., 2005a) and *P. cibarius* (Jung et al., 2005), both isolated from fermented seafood (Figure 5B).

Interestingly, the heat-shock genes *htpG_2* and *pka* exhibited very low conservation across all cold-adapted *Psychrobacter* strains (Figure 5). The *htpG_2* gene primarily functions as a molecular chaperone with ATPase activity (Chuang and Blattner, 1993), whereas *pka* acts mainly as a CoA-binding domain protein, with its heat-response role inferred from mutant phenotypes in *E. coli* (Ma and Wood, 2011). Heat-shock *mngR* and cold-shock *otsA* genes also showed reduced conservation along psychrophilic species, notably in the cases of *P. articus* (Bakermans et al., 2006; Ayala-del-Rio et al., 2010) and *P. urantivorans* (Bowman et al., 1996) (Figure 5A), and *htp*X heat-shock gene was absent in all psychrotrophic strains (Figure 5B). A notable absence was that of the cold-shock *otsA* gene in *P. raelei* (Manzulli et al., 2024) among psychrotolerant species, while present in all other *Psychrobacter* counterparts (Figure 5B).

These clustering patterns (Figure 5) show that SC65A.3 genes are largely conserved across cold-adapted *Psychrobacter* species, particularly in psychrophilic homologs, but with a notable divergence at the level of specific genes, reflecting functional specialization among these cold-environment bacteria. The absence or low conservation of the *htpG_2*, *pka*, and *htpX* heat-shock genes across all analyzed psychrotrophic species, in addition to cold-shock *otsA* absence in particular strains, suggests that these organisms rely on molecular mechanisms for thermal adaptation that differ from those used by psychrophilic strains.

### 4.3 Dual antimicrobial resistance and bioactive potential of *Psychrobacter* SC65A.3 strain

To date, the antimicrobial resistance of *Psychrobacter* species is poorly studied (Bowman, 2006). According to the NCBI Taxonomy Database (Schoch et al., 2020), from the 49 *Psychrobacter* strains identified and accepted as different species, only 18 strains, classified as psychrophiles, were tested for the antibiotic resistance to at least one type of antibiotic, most frequently from the penicillin class, and only five of them have undergone testing to a large set of antibiotics for establishing their antimicrobial resistance profiles (Table 5).

**Table 5:** Comparative antibiotic susceptibility/resistance profile of *Psychrobacter SC65A.3* and other *Psychrobacter* strains.

Antimicrobial spectrum	Antimicrobial class	Code ATB	1	2*	3*	4*	5*	6*	7	8	9	10	11	12	13	14	15	16	17	18	19
Broad spectrum	Penicillins	AMP	S	S	S	S	S	**R**	S	**R**	**R**	**R**	S	S	**R**		S	S	**R**	S	S
		CAR	S	S	**⁑R**	S	**R**	**R**		**R** ^**§**^	S^§^	S^§^	S^§^	S			S		**R** ^**§**^		
	Cephalosporins	CAZ	**R**																		
		CFM	**S**																		
		CTX	S											S			S				
		CPO	S																		
		KF	**R**		**⁑**S					S	S	S							S		
		CPD	S																		
	Carbapenems	IPM	S																		
	Fluoroquinolones	CIP	**R**																		S
		NA	S	S		S	S	S	**R**							S					S
	Aminoglycosides	CN	S	S	**⁑**S	S	S	S		S	S	S		**R**			S		S		S
		SH	R																		S
		S	S	S		S	S	**R**	S	S	S	S	S	**R**			S		S	S	S
	Coumarin glycosides	NV	**S**			S	**R**			**R**	**R**	**R**							**R**		
	Chloramphenicol	C	S	S	**⁑**S	S	S	S	**R**	S	S	S		S			S		S	S	S
	Nitrofurantoin	F	S	S		**R**	S	**R**													
	Rifampin	RD	**R**	S		S	S	S						S			**R**				S
	Sulfonamide compounds S3	S3	S																		
	Tetracyclines	TE	S	S		**R**	**R**	S	**R**	**R**	**R**	**R**	**R**	**R**			**R**		**R**	S	**R**
	Trimethoprim/Sulfonamides	W	**R**					S													
Narrow spectrum (G+)	Macrolides	CLR	**S**											S			S				
		E	**S**	S	**⁑**S	S	**R**	**R**	**R**	S										S	
	Lincosamide	DA	**R**																		
		MY	**R**	**R**		S	**R**	**R**					**R**	**R**		S	**R**				
	Fatty Acyls	MUP	**S**																		
	Glycopeptides	VA	**R**						**R**					**R**		S	**R**				
Anaerobes	Metronidazole	MTZ	**R**																		

1. *Psychrobacter* SC65A.3 from Scarisoara Cave. **Psychrophilic strains** (*): 2. *P. aquaticus* CMS56 (Shivaji et al., 2005); 3. *P. immobilis* PG1 (Juni and Heym, 1986; Gini, 1990); 4. *P. proteolyticus* 116 (Romanenko et al., 2002; Shivaji et al., 2004); 5. *P. submarinus* KMM225 (Romanenko et al., 2002; Shivaji et al., 2004); 6. *P. vallis* CMS39 (Shivaji et al., 2005). **Psychrotrophic strains**: 7. *P. aestuarii* SC35 (Baik et al., 2010); 8. P. alimentarius JG-100 (Yoon et al., 2005a); 9. *P. aquimaris* SW-210 (Yoon et al., 2005b); 10. *P. celer* 91 (Yoon et al., 2005c); 11. *P. cibarius* JG-219 (Jung et al., 2005); 12. *P. coccoides* F1192 (Shang et al., 2022); 13. *P. fozii* NF23 (Bozal et al., 2003); 14. *P. glaciei* BIc20019 (Zeng et al., 2016); 15. P. halodurans F2608 (Shang et al., 2022); 16. *P. luti* NF11 (Bozal et al., 2003); 17. *P. namhaensis* SW-242 (Yoon et al., 2005b); 18. *P. phenylpyruvicus* ACAM535 (Bovre and Henriksen, 1967; Bowman et al., 1996); 19. *P. pocilloporae* S6-60 (Zachariah et al., 2016). (**⁑**): clinical strains (Gini, 1990); (§): resistance/susceptibility to penicillin class found in literature; (red /R): resistance; (blue /S): susceptibility; (white): not tested. No significance of the bold values.

Our study reports the antibiotic susceptibility profiles of the *Psychrobacter* SC65A.3 strain to a broad panel of antibiotics. Compared to *Psychrobacter* species listed in the NCBI Taxonomy Database (Schoch et al., 2020), *Psychrobacter* SC65A.3 displays the most extensive antibiotic resistance profile, carrying genes associated with resistance to both antibiotics and toxic compounds (Table 4). This finding aligns with a previous genomic study of three *Psychrobacter* sp. strains, which also identified genes involved in antibiotic and toxic compound resistance, as well as mercury detoxification and metabolism, highlighting the promising potential of this genus for bioremediation applications (Lasa and Romalde, 2017). Moreover, this is the first report providing phenotypic and genotypic data regarding the susceptibility or resistance of a *Psychrobacter* strain (SC65A.3) to the following antibiotics: ceftazidime, trimethoprim, clindamycin (lincosamide class) and metronidazole (Table 5).

The currently investigated ice cave *Psychrobacter* strain showed resistance to 10 antibiotics from eight classes, which was mostly dissimilar to other *Psychrobacter* strains (Table 3, Table 5). Remarkably, SC65A.3 exhibited resistance to clinically relevant antibiotics for Gram-negative infections, such as *β*-lactams (incl. Third generation cephalosporins), fluoroquinolones and aminoglycosides. This particular resistance profile suggests that cold-adapted strains can act as reservoirs of resistance genes, which could be transferred to pathogens under selective pressure, mostly in aquatic or food-related environments.

The presence of *ampC* gene in SC65A.3 suggests a mechanism for β-lactam resistance through the hydrolysis of β-lactam antibiotics (Mora-Ochomogo and Lohans, 2021). AmpC β-lactamases are enzymes produced by many Gram-negative bacteria and confer resistance to a broad range of β-lactam antibiotics, particularly penicillins, cephalosporins (including cephalotin and ceftazidime) and monobactams. They are typically not inhibited by classical β-lactamase inhibitors such as clavulanic acid. Although in many bacteria, *ampC* is normally expressed at low (basal) levels, certain β-lactams (e.g., cefoxitin, imipenem) can induce *ampC* expression via regulatory genes such as *ampR* (Jacoby, 2009). Also, mutations in regulatory regions (e.g., promoter or repressor genes) can lead to constitutive overexpression, causing high-level resistance even without antibiotic exposure (Zaunbrecher et al., 2009). Some AmpC variants are carried on plasmids, allowing horizontal transfer between different bacterial species (Jacoby, 2009). Moreover, AmpC-producing strains often show multidrug resistance, such is the case of our strain, particularly when combined with efflux pumps (harbored by our strain), which reduce intracellular antibiotic concentrations. They can complicate treatment because *β*-lactam/β-lactamase inhibitor combinations are often ineffective, and third-generation cephalosporins may fail (Pérez-Pérez and Hanson, 2002; Jacoby, 2009; Bush, 2018).

Moreover, SC65A.3 genome harbored genes associated with phenotypic resistance to quinolones, likely resulting from mutations in the *gyrA, gyrB, parC,* and *parE* genes, leading to alterations in DNA gyrase and topoisomerase IV, the primary targets of fluoroquinolones (Redgrave et al., 2014; Manik et al., 2025). These mutations can be transferred via mobile genetic elements (MGEs), contributing to the spread of fluoroquinolone resistance (Sakalauskienė et al., 2025). Rifampin resistance in SC65A.3 strain may be linked to mutations in the *rpoB* gene encoding the *β*-subunit of RNA polymerase representing the primary target of this antibiotic (Fluit et al., 2001; Goldstein, 2014; Sudzinová et al., 2023). Resistance to aminoglycosides as well as to other antibiotics could be attributed to the presence of multidrug efflux pump genes (*mexA, mexB, oprM, emrK, mdtA, mdtK, macB, mepA, msbA,* and *macA* genes) that could actively expel different antimicrobial agents from bacterial cells (Huang et al., 2022). These genes are frequently located on MGEs (Serio et al., 2017; Naderi et al., 2023). Despite the lack of phenotypic resistance to tetracycline, the SC65A.3 strain harbored the *tetA* and *tetC* genes conferring resistance to tetracycline via efflux mechanisms. These genes are frequently located on plasmids and transposons, allowing for their dissemination across diverse bacterial species through horizontal gene transfer (HGT) (Roberts, 2005; Grossman, 2016). Trimethoprim resistance could be linked to the *dfrA* gene, through the production of altered target enzymes (Ambrose and Hall, 2021). These genes are commonly associated with integrons, which can capture and mobilize resistance gene cassettes, facilitating their spread (Muziasari et al., 2014; Jiang et al., 2023). Although the colistin susceptibility testing was not performed, SC65A.3 harbors the *mcr1* gene, responsible for the last resort antibiotic colistin resistance, which is located on plasmids and can be transferred between bacteria, including those of environmental origin, posing a risk for the emergence of colistin-resistant pathogens (Forde et al., 2018; Mondal et al., 2024).

Additional genes such as *arsA, arsB, arsC, bcr, emrE, merA, merP, merR1_1,* and *merR1_2* confer resistance to heavy metals and other compounds (Anedda et al., 2023; Sanchez-Corona et al., 2025). Moreover, arsenic and mercury genes could co-select for resistance to different antibiotics. Also, these genes are often located on plasmids and transposons, contributing to the adaptability and persistence of bacteria in various environments (Pal et al., 2017; Gillieatt and Coleman, 2024).

The presence of these clinically important resistance genes in SC65A.3, a psychrophilic strain, emphasizes the role of environmental bacteria as reservoirs of resistance determinants (Larsson and Flach, 2022; Martak et al., 2024). The mobility of these genes via MGEs facilitates their transfer to pathogenic bacteria, potentially leading to the emergence of MDR pathogens. This underscores the importance of monitoring environmental sources for AMR genes and implementing strategies to mitigate their spread (Bengtsson-Palme et al., 2023).

A study from 2002 on nonclinical *Psychrobacter* strains revealed their resistance to most penicillins (*pilQ, porB, ftsI, nagZ, cpxR, zraS_1, zraS_2, zraR, ampC, ampG*) (Romanenko et al., 2002), while a *Psychrobacter* strain isolated from 35,000-years old permafrost was resistant to tetracycline (*tetA, tetC, emrK*) and streptomycin (*emrE, rpsE, rpsD,rsmG, rpsL, mipA*) (Petrova et al., 2009). Also, the sensitivity of various *Psychrobacter* strains, including three psychrotrophic marine *Psychrobacter* isolates from the Mediterranean Sea, Egypt, to tetracycline, ampicillin and chloramphenicol among other antibiotics was reported (Dziewit et al., 2013; Jeong et al., 2013; Abd-Elnaby et al., 2016).

The responses of five psychrophilic *Psychrobacter* strains to the same set of antibiotics differed from that observed in SC65A.3 (Table 5). *P. aquaticus* CMS56 (Shivaji et al., 2005), *P. proteolyticus* 116 (Denner et al., 2001; Romanenko et al., 2002), *P. submarinus* KMM225 (Romanenko et al., 2002) and *P. immobilis* PG1 associated with fish, processed meat, poultry products (Juni and Heym, 1986) showed sensitivity to ampicillin, oppositely to the Scarisoara strain, while the clinically isolated *P. immobilis* was resistant to penicillin (Gini, 1990), and *P. vallis* CMS39 (Shivaji et al., 2005) was resistant to ampicillin and carbenicillin (Table 5). Cephalosporin testing was performed only on the clinically isolated *P. immobilis* strain, which was sensitive to cephalothin, in contrast to the resistance observed in strain SC65A.3 (Table 5). The *Psychrobacter vallis* CMS39 strains was sensitive to trimethoprim, unlike the Scarisoara strain which showed resistance to this antibiotic (*dfrA, dut, mdtK*). Furthermore, SC65A.3 was resistant to (*porB, pvdQ, acrR, oprM, mexB, mexA, mdtK, mepA*) and spectinomycin (*rpsE*), while *Psychrobacter pocilloporae* S6-60, a psychrotrophic bacteria isolated from the mucus of the coral *Pocillopora eydouxi*, was susceptible to both antibiotics (Zachariah et al., 2016) and also to rifampicin (*rpoB, rpoC, rpoA*), similar to the psychrothrophic strains *P. aestuarii* SC35 (Baik et al., 2010), *P. coccoides* F1192 (Shang et al., 2022) and *P. halodurans* F2608 (Shang et al., 2022), respectively (Table 5). Meanwhile, the other five analyzed *Psychrobacter* strains were either sensitive to these antibiotics, or no testing was done (Table 5).

A comparable pattern was observed for trimethoprim resistance (genes *dfrA*, *dut*, *mdtK*), where the only other tested *Psychrobacter* strain was the psychrophilic *P. vallis* CMS39, isolated from a cyanobacterial mat in Antarctica (Shivaji et al., 2005) (Table 5). Moreover, SC65A.3 showed resistance to cephalothin, whereas all other tested *Psychrobacter* strains, including *P. immobilis* PG1 (Gini, 1990), and the psychrotolerant *P. alimentarius* PAMC 27889 (Yoon et al., 2005a), *P. aquimaris* SW-210 (Yoon et al., 2005b), *P. celer* 91 (Yoon et al., 2005c), *P. coccoides* F1192 (Shang et al., 2022), *P. halodurans* F2608 (Shang et al., 2022) and *P. namhaensis* SW-242 (Yoon et al., 2005b) were susceptible (Table 5). In the case of lincomycin and vancomycin (*walR, murG*) SC65A.3 showed resistance similar to the majority of other *Psychrobacter* strains except for *P. proteolyticus* 116 (Romanenko et al., 2002; Shivaji et al., 2004) and *P. aquaticus* CMS56 (Shivaji et al., 2005) that were susceptible to lincomycin. Moreover, the psychrotolerant strain *P. glaciei* BIc20019 isolated from an Arctic glacier ice core (Zeng et al., 2016) showed susceptibility to both lincomycin and vancomycin (Table 5).

Scarisoara SC65A.3 strain has been susceptible to tetracycline, despite the presence of tetracycline resistance genes *tetA* and *tetC* (Figure 4, Table 5), while other *Psychrobacter* strains showed resistance for the most part, with the exception of two psychrophilic and one psychrotolerant strains showing susceptibility. Scarisoara strain was also susceptible to ampicillin, cefotaxime, streptomycin and chloramphenicol, similar to the other various *Psychrobacter* strains (Table 5). Six *Psychrobacter* strains isolated from bird guano from the Arctic Spirtsbergen Island were also found to be susceptible to ampicillin, chloramphenicol and tetracycline (Dziewit et al., 2013).

**Figure 6:**
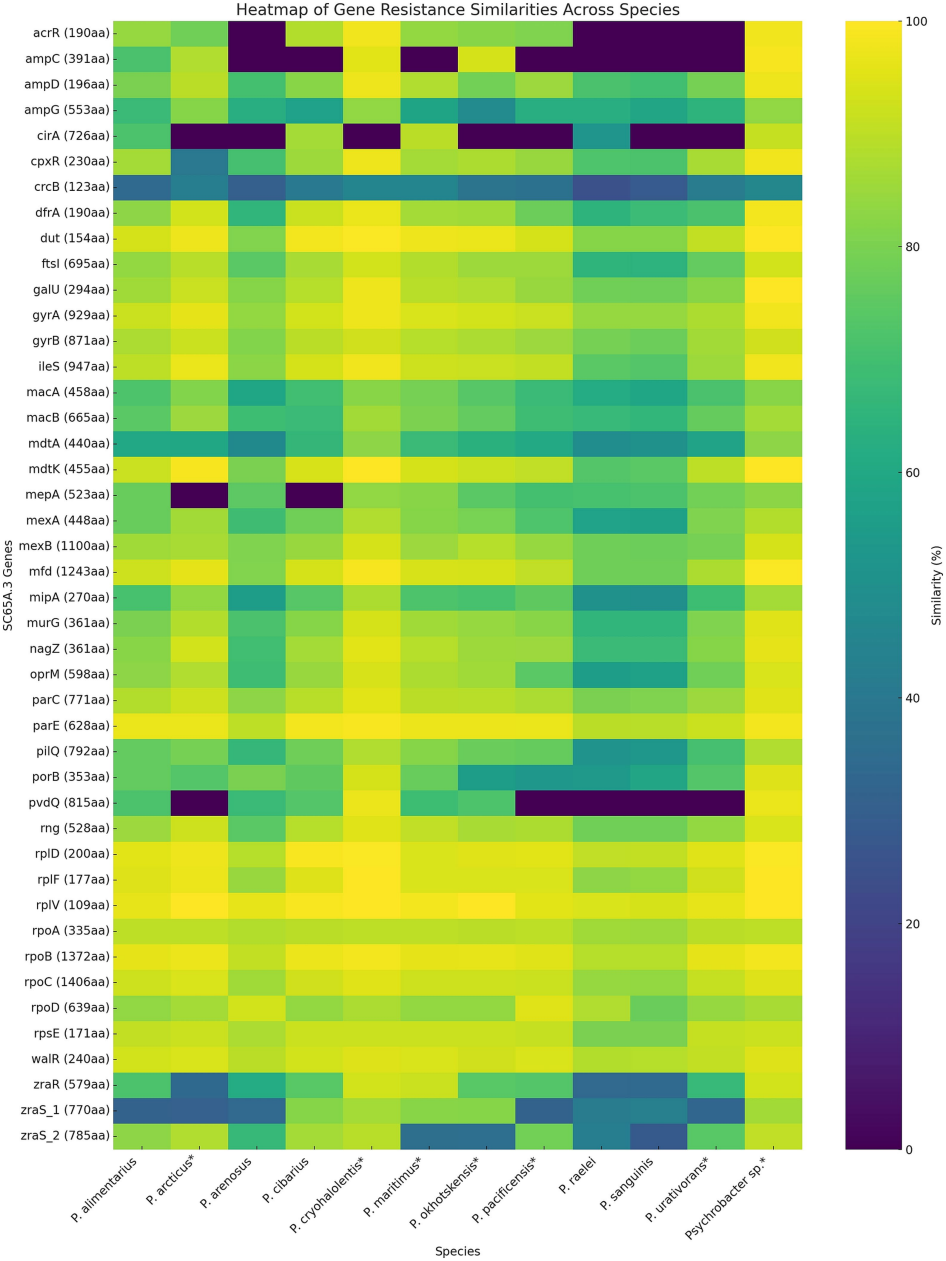
Heatmap distribution of the antibiotic resistance genes in SC65A.3 and 12 other *Psychrobacter* species genomes. The ARGs correspond to the phenotypic resistance (Table 4); (*): psychrophilic strains.

Heatmap comparative analysis of resistance-associated genes across *Psychrobacter* species (Figure 6) revealed both highly conserved determinants and more variable species-specific elements. The conservation patterns highlight the evolutionary balance between the essential functions of resistance genes and the adaptive pressures shaping their diversification. A subset of genes, including *gyrA* and *gyrB* (DNA gyrase), *parC* and *parE* (topoisomerase IV) core drug target genes, as well as *rpoA*, *rpoB*, *rpoC* (RNA polymerase subunits), and *rplD*, *rplF*, *rplV*, *rpsE* (ribosomal proteins), demonstrated strong conservation across all examined species (Figure 6). These findings support the view that such loci function as essential housekeeping genes subject to strong purifying selection, consistent with prior observations that core genes show limited polymorphism in prokaryotes under functional constraint (Bohlin et al., 2017; Joshi et al., 2022). Although mutations in housekeeping genes such as *gyrA*, *rpoB*, or *rpsL* can confer resistance to fluoroquinolones, rifampicins, and macrolides, respectively, the high conservation observed across species reflects the indispensable cellular roles of these genes, which strongly limit tolerated sequence variation (Vester and Douthwaite, 2001; Chiribau et al., 2024).

Efflux systems represented a second category of resistance determinants across *Psychrobacter* species’ resistome (Figure 6). Genes such as *mexA*, *mexB*, and *oprM* were broadly conserved, suggesting that multidrug efflux is a widespread strategy among *Psychrobacter* species and explaining its MDR phenotype (Zgurskaya, 2002; Ayala-del-Rio et al., 2010; Huang et al., 2022). In contrast, some efflux-related genes, such as *mdtA*, *mdtK*, and *mexA*, exhibited moderate variability, likely representing species-specific adaptations in substrate specificity or regulatory mechanisms that mirror the ecological diversity of the analyzed taxa (Kim et al., 2010; Zhang et al., 2017; Glavier et al., 2020). Similarly, the *ampC β*-lactamase and its regulator *ampD* showed conservation in most species, but with notable divergence in some, suggesting differing potentials for β-lactam resistance (Mallik et al., 2022; Laranjeiro et al., 2025). In contrast, several genes, including *cirA* (outer membrane receptor), *pvdQ* (quorum-quenching enzyme), and regulatory elements such as *zraR* and *zraS*, exhibited limited similarity or were absent from subsets of species (Figure 6). The patchy distribution and low conservation of these genes point toward niche-specific functions, horizontal acquisition, or lineage-specific losses (Alm et al., 2006; Ito et al., 2018; Rome et al., 2018). For instance, the divergence of *Psychrobacter* species in these loci likely may reflect adaptation to cold environments, where selective pressures on resistance mechanisms may differ from those acting on mesophilic *Psychrobacter*.

Several enzymes detected in the SC65.3 genome (Supplementary Table S5) could also play a direct role in the processes underlying antibiotic resistance. Among these, β-lactamases—such as those associated with the Lactamase_B domain (COG2440/KEGG: K01413)—stand out for their ability to hydrolyze the β-lactam ring characteristic of penicillins and cephalosporins (Bush and Jacoby, 2010). Moreover, UppP phosphatase (COG1396/KEGG: K06116), which hydrolyzes undecaprenyl diphosphate (C₅₅-PP) to regenerate undecaprenyl phosphate (C₅₅-P) involved in recycling the lipid carrier essential for cell wall biosynthesis could confer resistance to bacitracin and ensure continuous peptidoglycan synthesis under antibiotic stress (Bernard et al., 2005). Also, MATE family transporters (COG2006/KEGG: K06188) acting as efflux pumps expel antibiotic molecules—such as fluoroquinolones—out of the cytoplasm, using H^+^ or Na^+^ gradients as an energy source (Omote et al., 2006). Meanwhile, penicillin amidase (COG2366/KEGG: K07116; EC 3.5.1.97) contributes to the degradation of penicillin-type antibiotics by cleaving the amide bond between 6-aminopenicillanic acid and its acyl side chain (Pan et al., 2022). This enzyme implicated in antibiotic inactivation in clinical Gram-negative bacteria (Cole and Sutherland, 1966) has been isolated from various environmental gene pools (Gabor et al., 2005), although, although to date no documented case in *Psychrobacter* linking both environmental and clinical origins has been reported. Consequently, mechanisms such as hydrolysis, enzymatic modification, active efflux, and target protection appear to be recurring strategies of antimicrobial resistance (Blair et al., 2015; Munita and Arias, 2016; Reygaert, 2018). The presence of these pathways even in non-pathogenic species like *Psychrobacter cryohalolentis* highlights a profound ecological adaptation, with a diverse genetic profile shaped by the selective pressures of its natural environment (Blair et al., 2015).

Furthermore, the genome of this cave-derived *Psychrobacter* highlights its broad biotechnological potential through diverse hydrolytic enzymes also evidenced in the functional profile (Table 1). Notably, the previously reported recombinant lipase Lip2, cloned from this strain by our group (Paun et al., 2024), represents a new catalysts exhibiting both lipolytic and esterase activities, underscoring its applicability in various biocatalytic processes.

Overall, these findings suggest a two-tiered organization of resistance determinants in *Psychrobacter* SC65A.3 ice cave strain (Figure 6), comprising a conserved core of housekeeping resistance genes for ensuring baseline defense against clinically relevant antibiotics across the genus (yellow), and a variable accessory set contributing to ecological specialization and adaptive flexibility (blue-green). Such patterns underscore the potential of environmental *Psychrobacter* and related taxa to serve as reservoirs of clinically relevant resistance genes, while also emphasizing the evolutionary fluidity of non-core determinants.

## 5 Conclusion

The current data presenting a combined functional and genomic analysis of the *Psychrobacter* SC65A.3 strain isolated from 5,000-year-old ice of Scarisoara ice cave provided the first resistome characterization of an ice-derived bacterium from a subterranean environment. This psychrophilic isolate belonging to *P. cryohalolentis* species displays a distinctive phenotypic profile and gene repertoire associated with antibiotic resistance, antimicrobial activity, enzymatic potential, and thermal adaptation, highlighting opportunities for biotechnological and medical exploitation. The presence of a diverse set of AMR genes, correlated with a MDR phenotype, underscores its role as a potential reservoir of resistance determinants, a finding of particular concern given its environmental origin and highlights the importance of monitoring natural habitats as sources of AMR. Compared to other strains of this genus inhabiting cold environments, the psychrophilic SC65A.3 stands out for its broader phenotypic and genotypic resistance profiles and distinctive thermal adaptation genes. As the first whole-genome sequence from millennia-old cave ice, *Psychrobacter* SC65A.3 offers a valuable platform for advancing our understanding of ancient resistomes and represents a promising source of cold-active enzymes with potential applications across biotechnology, medicine, and industry.

## Data Availability

All data supporting the findings of this study are included in the manuscript. The 16S rRNA gene sequence of the *Psychrobacter* SC65A.3 bacterial strain isolated from Scarisoara Ice Cave has been deposited in GenBank under accession number MN577402 (https://www.ncbi.nlm.nih.gov/nuccore/MN577402.1). The complete genome sequence of *Psychrobacter* SC65A.3 is available in the GenBank database under accession number CP106752.1 (https://www.ncbi.nlm.nih.gov/nuccore/CP106752.1), with the NCBI RefSeq assembly number GCF_025642195.1 (https://www.ncbi.nlm.nih.gov/datasets/genome/GCF_025642195.1/).

## Footnotes

1
https://www.ezbiocloud.net/tools/ani

2
https://kbase.us

## References

[ref1] Abd-Elnaby H. M. Abou-Elela G. M. Ghozlan H. A. Hussein H. Sabry S. A. (2016). Characterization and bioremediation potential of marine *Psychrobacter* species. Egypt. J. Aquat. Res. 42, 193–203. doi: 10.1016/j.ejar.2016.04.003

[ref2] Abdul-Jabbar A. M. Hussian N. N. Mohammed H. A. Aljarbou A. Akhtar N. Khan R. A. (2022). Combined anti-bacterial actions of Lincomycin and freshly prepared silver nanoparticles: overcoming the resistance to antibiotics and enhancement of the bioactivity. Antibiotics 11:1791. doi: 10.3390/antibiotics11121791, 36551448 PMC9774316

[ref3] Abramov A. Vishnivetskaya T. Rivkina E. (2021). Are permafrost microorganisms as old as permafrost? FEMS Microbiol. Ecol. 97:fiaa260. doi: 10.1093/femsec/fiaa260, 33601419

[ref4] Aggarwal R. Mahajan P. Pandiya S. Bajaj A. Verma S. K. Yadav P. . (2024). Antibiotic resistance: a global crisis, problems and solutions. Crit. Rev. Microbiol. 50, 896–921. doi: 10.1080/1040841X.2024.2313024, 38381581

[ref5] Ali P. Chen F. Hassan F. Sosa A. Khan S. Badshah M. . (2021). Bacterial community characterization of Batura glacier in the Karakoram range of Pakistan. Int. Microbiol. 24, 183–196. doi: 10.1007/s10123-020-00153-x, 33404934

[ref6] Allen H. K. Donato J. Wang H. H. Cloud-Hansen K. A. Davies J. Handelsman J. (2010). Call of the wild: antibiotic resistance genes in natural environments. Nat. Rev. Microbiol. 8, 251–259. doi: 10.1038/nrmicro2312, 20190823

[ref7] Alm E. Huang K. Arkin A. (2006). The evolution of two-component Systems in Bacteria Reveals Different Strategies for niche adaptation. PLoS Comput. Biol. 2:e143. doi: 10.1371/journal.pcbi.0020143, 17083272 PMC1630713

[ref8] Altschul S. F. Gish W. Miller W. Myers E. W. Lipman D. J. (1990). Basic local alignment search tool. J. Mol. Biol. 215, 403–410. doi: 10.1016/S0022-2836(05)80360-2, 2231712

[ref9] Ambrose S. J. Hall R. M. (2021). *dfrA* trimethoprim resistance genes found in Gram-negative bacteria: compilation and unambiguous numbering. J. Antimicrob. Chemother. 76, 2748–2756. doi: 10.1093/jac/dkab212, 34180526

[ref10] Ambrozic Avgustin J. Petrič P. Pašić L. (2019). Screening the cultivable cave microbial mats for the production of antimicrobial compounds and antibiotic resistance. IJS 48, 295–303. doi: 10.5038/1827-806X.48.3.2272

[ref11] Anedda E. Farrell M. L. Morris D. Burgess C. M. (2023). Evaluating the impact of heavy metals on antimicrobial resistance in the primary food production environment: A scoping review. Environ. Pollut. 320:121035. doi: 10.1016/j.envpol.2023.121035, 36623784

[ref12] Anesio A. M. Laybourn-Parry J. (2012). Glaciers and ice sheets as a biome. Trends Ecol. Evol. 27, 219–225. doi: 10.1016/j.tree.2011.09.012, 22000675

[ref13] Anesio A. M. Lutz S. Chrismas N. A. M. Benning L. G. (2017). The microbiome of glaciers and ice sheets. NPJ Biofilms Microb. 3:10. doi: 10.1038/s41522-017-0019-0, 28649411 PMC5460203

[ref14] Arkin A. P. Cottingham R. W. Henry C. S. Harris N. L. Stevens R. L. Maslov S. . (2018). KBase: the United States Department of Energy Systems Biology Knowledgebase. Nat. Biotechnol. 36, 566–569. doi: 10.1038/nbt.4163, 29979655 PMC6870991

[ref15] Aslam B. Wang W. Arshad M. I. Khurshid M. Muzammil S. Rasool M. H. . (2018). Antibiotic resistance: a rundown of a global crisis. IDR Volume 11, 1645–1658. doi: 10.2147/IDR.S173867 PMC6188119 30349322

[ref16] Ayala-del-Rio H. L. Chain P. S. Grzymski J. J. Ponder M. A. Ivanova N. Bergholz P. W. . (2010). The genome sequence of *Psychrobacter arcticus* 273-4, a psychroactive Siberian permafrost bacterium, reveals mechanisms for adaptation to low-temperature growth. Appl. Environ. Microbiol. 76, 2304–2312. doi: 10.1128/AEM.02101-09, 20154119 PMC2849256

[ref17] Bachy C. López-García P. Vereshchaka A. Moreira D. (2011). Diversity and vertical distribution of microbial eukaryotes in the snow, sea ice and seawater near the north pole at the end of the polar night. Front. Microbiol. 2:102. doi: 10.3389/fmicb.2011.00106, 21833337 PMC3153057

[ref18] Baik K. S. Park S. C. Lim C. H. Lee K. H. Jeon D. Y. Kim C. M. . (2010). *Psychrobacter aestuarii* sp. nov., isolated from a tidal flat sediment. Int. J. Syst. Evol. Microbiol. 60, 1631–1636. doi: 10.1099/ijs.0.016782-0, 19717579

[ref19] Bakermans C. (2018). Adaptations to marine versus terrestrial low temperature environments as revealed by comparative genomic analyses of the genus Psychrobacter. FEMS Microbiol. Ecol. 94:1–16. doi: 10.1093/femsec/fiy102, 29868789

[ref20] Bakermans C. Ayala-del-Río H. L. Ponder M. A. Vishnivetskaya T. Gilichinsky D. Thomashow M. F. . (2006). *Psychrobacter cryohalolentis* sp. nov. and *Psychrobacter arcticus* sp. nov., isolated from Siberian permafrost. Int. J. Syst. Evol. Microbiol. 56, 1285–1291. doi: 10.1099/ijs.0.64043-0, 16738105

[ref21] Bakermans C. Tsapin A. I. Souza-Egipsy V. Gilichinsky D. A. Nealson K. H. (2003). Reproduction and metabolism at − 10°C of bacteria isolated from Siberian permafrost. Environ. Microbiol. 5, 321–326. doi: 10.1046/j.1462-2920.2003.00419.x, 12662179

[ref22] Bauer A. W. Kirby W. M. M. Sherris J. C. Turck M. (1966). Antibiotic susceptibility testing by a standardized single disk method. Am. J. Clin. Pathol. 45, 493–496. doi: 10.1093/ajcp/45.4_ts.493, 5325707

[ref23] Belov A. A. Cheptsov V. S. Manucharova N. A. Ezhelev Z. S. (2020). Bacterial communities of Novaya Zemlya archipelago ice and permafrost. Geosciences 10:67. doi: 10.3390/geosciences10020067

[ref24] Bengtsson-Palme J. Abramova A. Berendonk T. U. Coelho L. P. Forslund S. K. Gschwind R. . (2023). Towards monitoring of antimicrobial resistance in the environment: for what reasons, how to implement it, and what are the data needs? Environ. Int. 178:108089. doi: 10.1016/j.envint.2023.108089, 37441817

[ref25] Bernard R. El Ghachi M. Mengin-Lecreulx D. Chippaux M. Denizot F. (2005). BcrC from *Bacillus subtilis* acts as an Undecaprenyl pyrophosphate phosphatase in bacitracin resistance. J. Biol. Chem. 280, 28852–28857. doi: 10.1074/jbc.M413750200, 15946938

[ref26] Bhullar K. Waglechner N. Pawlowski A. Koteva K. Banks E. D. Johnston M. D. . (2012). Antibiotic resistance is prevalent in an isolated cave microbiome. PLoS One 7:e34953. doi: 10.1371/journal.pone.0034953, 22509370 PMC3324550

[ref27] Biondi N. Tredici M. R. Taton A. Wilmotte A. Hodgson D. A. Losi D. . (2008). Cyanobacteria from benthic mats of Antarctic lakes as a source of new bioactivities. J. Appl. Microbiol. 105, 105–115. doi: 10.1111/j.1365-2672.2007.03716.x 18217933

[ref28] Blair J. M. A. Webber M. A. Baylay A. J. Ogbolu D. O. Piddock L. J. V. (2015). Molecular mechanisms of antibiotic resistance. Nat. Rev. Microbiol. 13, 42–51. doi: 10.1038/nrmicro3380 25435309

[ref29] Bogdan D. F. Baricz A. I. Chiciudean I. Bulzu P.-A. Cristea A. Năstase-Bucur R. . (2023). Diversity, distribution and organic substrates preferences of microbial communities of a low anthropic activity cave in North-Western Romania. Front. Microbiol. 14:962452. doi: 10.3389/fmicb.2023.962452, 36825091 PMC9941645

[ref30] Bohlin J. Eldholm V. Pettersson J. H. O. Brynildsrud O. Snipen L. (2017). The nucleotide composition of microbial genomes indicates differential patterns of selection on core and accessory genomes. BMC Genomics 18:151. doi: 10.1186/s12864-017-3543-7, 28187704 PMC5303225

[ref31] Bonwitt J. Tran M. Droz A. Gonzalez A. Glover W. A. (2018). Psychrobacter sanguinis</i> wound infection associated with marine environment exposure, Washington, USA. Emerg. Infect. Dis. 24, 1942–1944. doi: 10.3201/eid2410.171821, 30226173 PMC6154140

[ref32] Bovre K. Henriksen S. D. (1967). A revised description of *Moraxella polymorpha* Flamm 1957, with a proposal of a new name, *Moraxella phenylpyrouvica* for this species. Int. J. Syst. Bacteriol. 17, 343–360. doi: 10.1099/00207713-17-4-343

[ref33] Bowman J. P. (2006). “The genus Psychrobacter” in The Prokaryotes. eds. Dworkin M. Falkow S. Rosenberg E. Schleifer K.-H. Stackebrandt E. (New York, NY: Springer New York).

[ref34] Bowman J. P. Cavanagh J. Austin J. J. Sanderson K. (1996). Novel *Psychrobacter* species from Antarctic ornithogenic soils. Int. J. Syst. Bacteriol. 46, 841–848. doi: 10.1099/00207713-46-4-841, 8863407

[ref35] Bowman J. P. Nichols D. S. McMeekin T. A. (1997). *Psychrobacter glacincola* sp. nov., a halotolerant, psychrophilic bacterium isolated from Antarctic Sea ice. Syst. Appl. Microbiol. 20, 209–215. doi: 10.1016/S0723-2020(97)80067-7

[ref36] Bozal N. Montes M. J. Tudela E. Guinea J. (2003). Characterization of several Psychrobacter strains isolated from Antarctic environments and description of *Psychrobacter luti* sp. nov. and *Psychrobacter fozii* sp. nov. Int. J. Syst. Evol. Microbiol. 53, 1093–1100. doi: 10.1099/ijs.0.02457-0, 12892132

[ref37] Brad T. Itcus C. Pascu M.-D. Persoiu A. Hillebrand-Voiculescu A. Iancu L. . (2018). Fungi in perennial ice from Scărișoara Ice Cave (Romania). Sci. Rep. 8:10096. doi: 10.1038/s41598-018-28401-1, 29973683 PMC6031636

[ref38] Bull A. T. Ward A. C. Goodfellow M. (2000). Search and discovery strategies for biotechnology: the paradigm shift. Microbiol. Mol. Biol. Rev. 64, 573–606. doi: 10.1128/MMBR.64.3.573-606.2000, 10974127 PMC99005

[ref39] Bush K. (2018). Past and present perspectives on β-lactamases. Antimicrob. Agents Chemother. 62, e01076–e01018. doi: 10.1128/AAC.01076-18, 30061284 PMC6153792

[ref40] Bush K. Jacoby G. A. (2010). Updated functional classification of β-lactamases. Antimicrob. Agents Chemother. 54, 969–976. doi: 10.1128/AAC.01009-09, 19995920 PMC2825993

[ref41] Caspar Y. Recule C. Pouzol P. Lafeuillade B. Mallaret M.-R. Maurin M. . (2013). *Psychrobacter arenosus* bacteremia after blood transfusion, France. Emerg. Infect. Dis. 19, 1118–1120. doi: 10.3201/eid1907.121599, 23764120 PMC3713977

[ref42] Chaumeil P.-A. Mussig A. J. Hugenholtz P. Parks D. H. (2020). GTDB-Tk: a toolkit to classify genomes with the genome taxonomy database. Bioinformatics 36, 1925–1927. doi: 10.1093/bioinformatics/btz848, 31730192 PMC7703759

[ref43] Che S. Song L. Song W. Yang M. Liu G. Lin X. (2013). Complete genome sequence of Antarctic bacterium <i>Psychrobacter</i> sp. strain G. Genome Announc. 1, e00725–e00713. doi: 10.1128/genomeA.00725-13, 24051316 PMC3778199

[ref44] Chen Y. Liu K. Liu Y. Vick-Majors T. J. Wang F. Ji M. (2022). Temporal variation of bacterial community and nutrients in Tibetan glacier snowpack. Cryosphere 16, 1265–1280. doi: 10.5194/tc-16-1265-2022

[ref45] Chen H. Tseng C. C. Hubbard B. K. Walsh C. T. (2001). Glycopeptide antibiotic biosynthesis: enzymatic assembly of the dedicated amino acid monomer (*S*)-3,5-dihydroxyphenylglycine. Proc. Natl. Acad. Sci. USA 98, 14901–14906. doi: 10.1073/pnas.221582098, 11752437 PMC64956

[ref46] Chiribau C. B. Schmedes S. Dong Y. Tarigopula N. Tekin O. Cannons A. . (2024). Detection of resistance to macrolides and fluoroquinolones in *Mycoplasma genitalium* by targeted next-generation sequencing. Microbiol. Spectr. 12, e03845–e03823. doi: 10.1128/spectrum.03845-23, 38349187 PMC10913745

[ref47] Chuang S. E. Blattner F. R. (1993). Characterization of twenty-six new heat shock genes of *Escherichia coli*. J. Bacteriol. 175, 5242–5252. doi: 10.1128/jb.175.16.5242-5252.1993, 8349564 PMC204992

[ref48] Cole M. Sutherland R. (1966). The role of penicillin Acylase in the resistance of Gram-negative Bacteria to Penicillins. J. Gen. Microbiol. 42, 345–356. doi: 10.1099/00221287-42-3-345, 5330787

[ref49] D’Costa V. M. King C. E. Kalan L. Morar M. Sung W. W. L. Schwarz C. . (2011). Antibiotic resistance is ancient. Nature 477, 457–461. doi: 10.1038/nature10388, 21881561

[ref50] Deming J. W. (2009). “Sea ice Bacteria and viruses” in Sea Ice. eds. Thomas D. N. Dieckmann G. S. (Oxford, UK: Wiley-Blackwell), 247–282. doi: 10.1002/9781444317145.ch7

[ref51] Denner E. B. M. Mark B. Mark B. Busse H.-J. Busse H.-J. Turkiewicz M. . (2001). *Psychrobacter proteolyticus* sp. nov., a Psychrotrophic, halotolerant bacterium isolated from the Antarctic krill *Euphausia superba* Dana, excreting a cold-adapted metalloprotease. Syst. Appl. Microbiol. 24, 44–53. doi: 10.1078/0723-2020-00006, 11403398

[ref52] Dereeper A. Guignon V. Blanc G. Audic S. Buffet S. Chevenet F. . (2008). Phylogeny.fr: robust phylogenetic analysis for the non-specialist. Nucleic Acids Res. 36, W465–W469. doi: 10.1093/nar/gkn180, 18424797 PMC2447785

[ref53] Deschaght P. Janssens M. Vaneechoutte M. Wauters G. (2012). Psychrobacter isolates of human origin, other than *Psychrobacter phenylpyruvicus*, are predominantly Psychrobacter faecalis and *Psychrobacter pulmonis*, with emended description of *P. faecalis*. Int. J. Syst. Evol. Microbiol. 62, 671–674. doi: 10.1099/ijs.0.032631-0, 21551328

[ref54] Dziewit L. Cegielski A. Romaniuk K. Uhrynowski W. Szych A. Niesiobedzki P. . (2013). Plasmid diversity in arctic strains of Psychrobacter spp. Extremophiles 17, 433–444. doi: 10.1007/s00792-013-0521-0, 23479249 PMC3632715

[ref55] El-Sayed M. R. Emam A. M. Osman A. E. Abd El-Galil M. A. E.-A. A. Sayed H. H. (2023). Detection and description of a novel *Psychrobacter glacincola* infection in some Red Sea marine fishes in Hurghada, Egypt. BMC Vet. Res. 19:23. doi: 10.1186/s12917-022-03542-8, 36717850 PMC9885648

[ref56] Elshobary M. E. Badawy N. K. Ashraf Y. Zatioun A. A. Masriya H. H. Ammar M. M. . (2025). Combating antibiotic resistance: mechanisms, multidrug-resistant pathogens, and novel therapeutic approaches: An updated review. Pharmaceuticals 18:402. doi: 10.3390/ph18030402, 40143178 PMC11944582

[ref57] Fluit A. C. Visser M. R. Schmitz F.-J. (2001). Molecular detection of antimicrobial resistance. Clin. Microbiol. Rev. 14, 836–871. doi: 10.1128/CMR.14.4.836-871.2001, 11585788 PMC89006

[ref58] Forde B. M. Zowawi H. M. Harris P. N. A. Roberts L. Ibrahim E. Shaikh N. . (2018). Discovery of *mcr-1* -mediated colistin resistance in a highly virulent *Escherichia coli* lineage. mSphere 3, e00486–e00418. doi: 10.1128/mSphere.00486-18, 30305321 PMC6180223

[ref59] Gabor E. M. De Vries E. J. Janssen D. B. (2005). A novel penicillin acylase from the environmental gene pool with improved synthetic properties. Enzym. Microb. Technol. 36, 182–190. doi: 10.1016/j.enzmictec.2004.04.021

[ref60] Gatinho P. Salvador C. Gutierrez-Patricio S. Macedo-Arantes S. Martins M. R. Silva A. M. . (2024). From cultural and natural heritage to reservoir of biomedicine: prospection of bioactive compounds produced by bacterial isolates from caves. Int. Biodeterior. Biodegrad. 190:105773. doi: 10.1016/j.ibiod.2024.105773

[ref61] Gattinger D. Pichler K. Weil T. Sattler B. (2023). A comparative approach to confirm antibiotic-resistant microbes in the cryosphere. Front. Microbiol. 14:1212378. doi: 10.3389/fmicb.2023.1212378, 37601352 PMC10435281

[ref62] Gaurav A. Bakht P. Saini M. Pandey S. Pathania R. (2023). Role of bacterial efflux pumps in antibiotic resistance, virulence, and strategies to discover novel efflux pump inhibitors. Microbiology 169:001333. doi: 10.1099/mic.0.001333, 37224055 PMC10268834

[ref63] Gesheva V. (2010). Production of antibiotics and enzymes by soil microorganisms from the windmill islands region, Wilkes Land, East Antarctica. Polar Biol. 33, 1351–1357. doi: 10.1007/s00300-010-0824-x

[ref64] Gheorghita G. R. Paun V. I. Neagu S. Maria G.-M. Enache M. Purcarea C. . (2021). Cold-active lipase-based biocatalysts for silymarin valorization through biocatalytic acylation of silybin. Catalysts 11:1390. doi: 10.3390/catal11111390

[ref65] Gillieatt B. F. Coleman N. V. (2024). Unravelling the mechanisms of antibiotic and heavy metal resistance co-selection in environmental bacteria. FEMS Microbiol. Rev. 48:fuae017. doi: 10.1093/femsre/fuae017, 38897736 PMC11253441

[ref66] Gini G. A. (1990). Ocular infection caused by *Psychrobacter immobilis* acquired in the hospital. J. Clin. Microbiol. 28, 400–401. doi: 10.1128/jcm.28.2.400-401.1990, 2312690 PMC269623

[ref67] Glavier M. Puvanendran D. Salvador D. Decossas M. Phan G. Garnier C. . (2020). Antibiotic export by MexB multidrug efflux transporter is allosterically controlled by a MexA-OprM chaperone-like complex. Nat. Commun. 11:4948. doi: 10.1038/s41467-020-18770-5, 33009415 PMC7532149

[ref68] Goldstein B. P. (2014). Resistance to rifampicin: a review. J. Antibiot. 67, 625–630. doi: 10.1038/ja.2014.107, 25118103

[ref69] Grant J. R. Enns E. Marinier E. Mandal A. Herman E. K. Chen C. . (2023). Proksee: in-depth characterization and visualization of bacterial genomes. Nucleic Acids Res. 51, W484–W492. doi: 10.1093/nar/gkad326, 37140037 PMC10320063

[ref70] Grossman T. H. (2016). Tetracycline antibiotics and resistance. Cold Spring Harb. Perspect. Med. 6:a025387. doi: 10.1101/cshperspect.a025387, 26989065 PMC4817740

[ref71] Gruner E. von Graevenitz A. Altwegg M. (1992). The API ZYM system: a tabulated review from 1977 to date. J. Microbiol. Methods 16, 101–118. doi: 10.1016/0167-7012(92)90030-8

[ref72] Gupta V. Chandran S. Deep A. Kumar R. Bisht L. (2022). Environmental factors affecting the diversity of psychrophilic microbial community in the high altitude snow-fed lake Hemkund, India. Curr. Res. Microb. Sci. 3:100126. doi: 10.1016/j.crmicr.2022.100126, 35909632 PMC9325733

[ref73] Haft D. H. DiCuccio M. Badretdin A. Brover V. Chetvernin V. O’Neill K. . (2018). RefSeq: an update on prokaryotic genome annotation and curation. Nucleic Acids Res. 46, D851–D860. doi: 10.1093/nar/gkx1068, 29112715 PMC5753331

[ref74] Harrington B. J. Gaydos J. M. (1984). Five-hour novobiocin test for differentiation of coagulase-negative staphylococci. J. Clin. Microbiol. 19, 279–280. doi: 10.1128/jcm.19.2.279-280.1984, 6699151 PMC271037

[ref75] Hazim F. A. Fasih F. Asif M. Iqbal S. N. Shaukat L. Sadia H. . (2020). Metronidazole susceptibility and resistance pattern among anaerobes causing periodontitis in tertiary care unit. PJMHS 14, 983–986.

[ref76] Hecht H. J. Sobek H. Haag T. Pfeifer O. Van Pée K.-H. (1994). The metal-ion-free oxidoreductase from *Streptomyces aureofaciens* has an α/β hydrolase fold. Nat. Struct. Mol. Biol. 1, 532–537. doi: 10.1038/nsb0894-532, 7664081

[ref77] Hemala L. Zhang D. Margesin R. (2014). Cold-active antibacterial and antifungal activities and antibiotic resistance of bacteria isolated from an alpine hydrocarbon-contaminated industrial site. Res. Microbiol. 165, 447–456. doi: 10.1016/j.resmic.2014.05.035, 24880083

[ref78] Heuchert A. Glöckner F. O. Amann R. Fischer U. (2004). *Psychrobacter nivimaris* sp. nov., a heterotrophic bacterium attached to organic particles isolated from the South Atlantic (Antarctica). Syst. Appl. Microbiol. 27, 399–406. doi: 10.1078/0723202041438455, 15368844

[ref79] Hillebrand-Voiculescu A. Itcus C. Ardelean I. Pascu D. Persoiu A. Rusu A. . (2014). Searching for cold-adapted microorganisms in the underground glacier of Scarisoara ice cave, Romania. Acta Carsologica 43:319–329. doi: 10.3986/ac.v43i2-3.604

[ref80] Hillebrand-Voiculescu A. Rusu A. Itcus C. Persoiu A. Brad T. Pascu M. D. . (2013). Bacterial 16S-rRNA gene clone library from recent ice stalagmites of Scărişoara cave. Rom. J. Biochem., 50:109–118.

[ref81] Hisar O. Yanik T. Hisar S. A. (2002). Clinical and pathological investigation of *Psychrobacter immobilis* infection in rainbow trour (*Oncorhynchus mykiss*, Walbaum). Isr. J. Aquacult. Bamidgeh 54:189–196. doi: 10.46989/001c.20324

[ref82] Holmlund P. Onac B. P. Hansson M. Holmgren K. Mörth M. Nyman M. . (2005). Assessing the palaeoclimate potential of cave glaciers: the example of the scǎrişoara ice cave (Romania). Geogr. Ann. Ser. A Phys. Geogr. 87, 193–201. doi: 10.1111/j.0435-3676.2005.00252.x

[ref83] Huang L. Wu C. Gao H. Xu C. Dai M. Huang L. . (2022). Bacterial multidrug efflux pumps at the frontline of antimicrobial resistance: An overview. Antibiotics 11:520. doi: 10.3390/antibiotics11040520, 35453271 PMC9032748

[ref84] Hubbard J. D. (2017). 3D Cave and Ice Block Morphology from Integrated Geophysical Methods: A Case Study at Scărişoara Ice Cave. Romania. USF Tampa Graduate Theses and Dissertations. Available at: https://digitalcommons.usf.edu/etd/6712

[ref85] Hudzicki J. (2009). Kirby–Bauer disk diffusion susceptibility test protocol. Am. Soc. Microbiol.

[ref86] Huerta-Cepas J. Szklarczyk D. Forslund K. Cook H. Heller D. Walter M. C. . (2016). eggNOG 4.5: a hierarchical orthology framework with improved functional annotations for eukaryotic, prokaryotic and viral sequences. Nucleic Acids Res. 44, D286–D293. doi: 10.1093/nar/gkv1248, 26582926 PMC4702882

[ref87] Humphries R. Bobenchik A. M. Hindler J. A. Schuetz A. N. (2021). Overview of changes to the clinical and laboratory standards institute performance standards for antimicrobial susceptibility testing, M100. J. Clin. Microbiol. 59, e00213–e00221. doi: 10.1128/JCM.00213-21, 34550809 PMC8601225

[ref88] Hurtado-Ortiz R. Nazimoudine A. Criscuolo A. Hugon P. Mornico D. Brisse S. . (2017). Psychrobacter pasteurii and Psychrobacter piechaudii sp. nov., two novel species within the genus Psychrobacter. Int. J. Syst. Evol. Microbiol. 67, 3192–3197. doi: 10.1099/ijsem.0.002065, 28840795

[ref89] Ioannou P. Ziogou A. Giannakodimos A. Giannakodimos I. Tsantes A. G. Samonis G. (2025). Psychrobacter infections in humans—A narrative review of reported cases. Antibiotics 14:140. doi: 10.3390/antibiotics14020140, 40001384 PMC11851457

[ref90] Itcus C. Pascu M.-D. Brad T. Persoiu A. Purcarea C. (2016). Diversity of cultured bacteria from the perennial ice block of Scarisoara ice cave, Romania. IJS 45, 89–100. doi: 10.5038/1827-806X.45.1.1948

[ref91] Itcus C. Pascu M. D. Lavin P. Persoiu A. Iancu L. Purcarea C. (2018). Bacterial and archaeal community structures in perennial cave ice. Sci. Rep. 8:15671. doi: 10.1038/s41598-018-34106-2, 30353134 PMC6199274

[ref92] Ito A. Sato T. Ota M. Takemura M. Nishikawa T. Toba S. . (2018). *In vitro* antibacterial properties of Cefiderocol, a novel Siderophore cephalosporin, against Gram-negative Bacteria. Antimicrob. Agents Chemother. 62, e01454–e01417. doi: 10.1128/AAC.01454-17, 29061741 PMC5740388

[ref93] Jacoby G. A. (2009). AmpC β-Lactamases. Clin. Microbiol. Rev. 22, 161–182. doi: 10.1128/CMR.00036-08, 19136439 PMC2620637

[ref94] Jansson A. Niemi J. Mäntsälä P. Schneider G. (2003). Crystal structure of aclacinomycin methylesterase with bound product analogues. J. Biol. Chem. 278, 39006–39013. doi: 10.1074/jbc.M304008200, 12878604

[ref95] Jaroszewicz W. Bielańska P. Lubomska D. Kosznik-Kwaśnicka K. Golec P. Grabowski Ł. . (2021). Antibacterial, antifungal and anticancer activities of compounds produced by newly isolated Streptomyces strains from the Szczelina Chochołowska cave (Tatra Mountains, Poland). Antibiotics 10:1212. doi: 10.3390/antibiotics10101212, 34680793 PMC8532742

[ref96] Jeong H.-J. Hyoungseok L. Soon G. H. Jang-Cheon C. Hong K. L. Yoo K. L. (2013). Transposon mutagenesis of *Psychrobacter cryohalolentis* PAMC 21807 by tri-parental conjugation. Adv. Polar Sci. 24:223. doi: 10.3724/SP.J.1085.2013.00223

[ref97] Jian Z. Zeng L. Xu T. Sun S. Yan S. Yang L. . (2021). Antibiotic resistance genes in bacteria: occurrence, spread, and control. J. Basic Microbiol. 61, 1049–1070. doi: 10.1002/jobm.202100201, 34651331

[ref98] Jiang Y. Peng K. Wang Q. Wang M. Li R. Wang Z. (2023). Novel trimethoprim resistance gene *dfrA49* identified in *Riemerella anatipestifer* from China. Microbiol. Spectr. 11, e04747–e04722. doi: 10.1128/spectrum.04747-22, 36916996 PMC10100655

[ref99] Joshi C. J. Ke W. Drangowska-Way A. O’Rourke E. J. Lewis N. E. (2022). What are housekeeping genes? PLoS Comput. Biol. 18:e1010295. doi: 10.1371/journal.pcbi.1010295, 35830477 PMC9312424

[ref100] Jung S.-Y. Lee M.-H. Oh T.-K. Park Y.-H. Yoon J.-H. (2005). *Psychrobacter cibarius* sp. nov., isolated from jeotgal, a traditional Korean fermented seafood. Int. J. Syst. Evol. Microbiol. 55, 577–582. doi: 10.1099/ijs.0.63398-0, 15774627

[ref101] Juni E. (2005) Genus III. Psychrobacter,”in Bergey’s Manual® of Systematic Bacteriology, eds. D. J. Brenner, N. R. Krieg, J. T. Staley, G. M. Garrity, D. R. Boone, P. De Vos, et al. (Boston, MA: Springer US). doi: 10.1007/0-387-28022-7

[ref102] Juni E. Heym G. A. (1986). *Psychrobacter immobilis* gen. Nov., sp. nov.: genospecies composed of Gram-negative, aerobic, oxidase-positive coccobacilli. Int. J. Syst. Bacteriol. 36, 388–391. doi: 10.1099/00207713-36-3-388

[ref103] Jurado V. Northup D. E. Saiz-Jimenez C. (2024). Microbial Roles in Caves. Lausanne: Frontiers Media SA. doi: 10.3389/978-2-8325-5188-2 PMC11167080 38868095

[ref104] Kampfer P. Glaeser S. P. Irgang R. Fernández-Negrete G. Poblete-Morales M. Fuentes-Messina D. . (2020). *Psychrobacter pygoscelis* sp. nov. isolated from the penguin *Pygoscelis papua*. Int. J. Syst. Evol. Microbiol. 70, 211–219. doi: 10.1099/ijsem.0.003739, 31617840

[ref105] Kampfer P. Jerzak L. Wilharm G. Golke J. Busse H.-J. Glaeser S. P. (2015). Psychrobacter ciconiae sp. nov., isolated from white storks (*Ciconia ciconia*). Int. J. Syst. Evol. Microbiol. 65, 772–777. doi: 10.1099/ijs.0.000013, 25479953

[ref106] Kaufmann B. B. Hung D. T. (2010). The fast track to multidrug resistance. Mol. Cell 37, 297–298. doi: 10.1016/j.molcel.2010.01.027, 20159549

[ref107] Kaur J. Sood U. Talwar C. Whitman W. B. Lal R. (2023). Phylogenomics-based reclassifications in the genus Psychrobacter including emended descriptions of *Psychrobacter pacificensis*, Psychrobacter proteolyticus and *Psychrobacter submarinus*. Antonie Van Leeuwenhoek 116, 1113–1121. doi: 10.1007/s10482-023-01871-8, 37640969

[ref108] Kim E. Cai Y. Yang S.-M. Lee W. Kim H.-Y. (2025). Psychrobacter saeujeotis sp. nov., a novel halophilic bacterium isolated from salted shrimp jeotgal. Int. J. Syst. Evol. Microbiol. 75:006734. doi: 10.1099/ijsem.0.006734, 40131329 PMC12281858

[ref109] Kim H. Kim M. Kim S. Lee Y. M. Shin S. C. (2022). Characterization of antimicrobial resistance genes and virulence factor genes in an Arctic permafrost region revealed by metagenomics. Environ. Pollut. 294:118634. doi: 10.1016/j.envpol.2021.118634, 34875269

[ref110] Kim H.-S. Nagore D. Nikaido H. (2010). Multidrug efflux pump MdtBC of *Escherichia coli* is active only as a B_2_ C Heterotrimer. J. Bacteriol. 192, 1377–1386. doi: 10.1128/JB.01448-09, 20038594 PMC2820868

[ref111] Kohanski M. A. DePristo M. A. Collins J. J. (2010). Sublethal antibiotic treatment leads to multidrug resistance via radical-induced mutagenesis. Mol. Cell 37, 311–320. doi: 10.1016/j.molcel.2010.01.003, 20159551 PMC2840266

[ref112] Konz D. Klens A. Schörgendorfer K. Marahiel M. A. (1997). The bacitracin biosynthesis operon of *Bacillus licheniformis* ATCC 10716: molecular characterization of three multi-modular peptide synthetases. Chem. Biol. 4, 927–937. doi: 10.1016/S1074-5521(97)90301-X, 9427658

[ref113] Kosznik-Kwasnicka K. Golec P. Jaroszewicz W. Lubomska D. Piechowicz L. (2022). Into the unknown: microbial communities in caves, their role, and potential use. Microorganisms 10:222. doi: 10.3390/microorganisms10020222, 35208677 PMC8877592

[ref114] Kralova S. Busse H.-J. Bezdíček M. Sandoval-Powers M. Nykrýnová M. Staňková E. . (2021). *Flavobacterium flabelliforme* sp. nov. and *Flavobacterium geliluteum* sp. nov., two multidrug-resistant psychrotrophic species isolated from Antarctica. Front. Microbiol. 12:729977. doi: 10.3389/fmicb.2021.729977, 34745033 PMC8570120

[ref115] Kumar R. Sharma R. C. (2021). Psychrophilic microbial diversity and physicochemical characteristics of glaciers in the Garhwal Himalaya, India. J. Microb. Biotech. Food Sci. 10:e2096. doi: 10.15414/jmbfs.2096

[ref116] Lange-Enyedi N. T. Németh P. Borsodi A. K. Spötl C. Makk J. (2024). Calcium carbonate precipitating extremophilic bacteria in an Alpine ice cave. Sci. Rep. 14:2710. doi: 10.1038/s41598-024-53131-y, 38302670 PMC10834452

[ref117] Laranjeiro L. G. Neme C. E. M. Previato-Mello M. Batista B. B. Henriques I. Da Silva Neto J. F. (2025). Mutations in *ampD* cause hyperproduction of AmpC and CmcB β-lactamases and high resistance to β-lactam antibiotics in *Chromobacterium violaceum*. Microbiol Spectr 13, e00916–e00925. doi: 10.1128/spectrum.00916-25, 40503828 PMC12323641

[ref118] Larsson D. G. J. Flach C.-F. (2022). Antibiotic resistance in the environment. Nat. Rev. Microbiol. 20, 257–269. doi: 10.1038/s41579-021-00649-x, 34737424 PMC8567979

[ref119] Lasa A. Romalde J. L. (2017). Genome sequence of three Psychrobacter sp. strains with potential applications in bioremediation. Genomics Data 12, 7–10. doi: 10.1016/j.gdata.2017.01.005, 28229046 PMC5312645

[ref120] Lee L.-H. Cheah Y.-K. Mohd Sidik S. Ab Mutalib N.-S. Tang Y.-L. Lin H.-P. . (2012a). Molecular characterization of Antarctic actinobacteria and screening for antimicrobial metabolite production. World J. Microbiol. Biotechnol. 28, 2125–2137. doi: 10.1007/s11274-012-1018-1, 22806035

[ref121] Lee L.-H. Cheah Y.-K. Nurul Syakima A. M. Shiran M. S. Tang Y.-L. Lin H.-P. . (2012b). Analysis of Antarctic proteobacteria by PCR fingerprinting and screening for antimicrobial secondary metabolites. Genet. Mol. Res. 11, 1627–1641. doi: 10.4238/2012.June.15.12, 22782582

[ref122] Lee D. D. Galera-Laporta L. Bialecka-Fornal M. Moon E. C. Shen Z. Briggs S. P. . (2019). Magnesium flux modulates ribosomes to increase bacterial survival. Cell 177:e13, 352–360. doi: 10.1016/j.cell.2019.01.042, 30853217 PMC6814349

[ref123] Lee J. Kwon M. Yang J. Y. Woo J. Lee H. K. Hong S. G. . (2016). Complete genome sequence of *Psychrobacter alimentarius* PAMC 27889, a Psychrophile isolated from an Antarctic rock sample. Genome Announc. 4, e00704–e00716. doi: 10.1128/genomeA.00704-16, 27445386 PMC4956459

[ref124] Letunic I. Bork P. (2024). Interactive tree of life (iTOL) v6: recent updates to the phylogenetic tree display and annotation tool. Nucleic Acids Res. 52, W78–W82. doi: 10.1093/nar/gkae268, 38613393 PMC11223838

[ref125] Lewis K. (2020). The science of antibiotic discovery. Cell 181, 29–45. doi: 10.1016/j.cell.2020.02.056, 32197064

[ref126] Li A.-Z. Han X.-B. Zhang M.-X. Zhou Y. Chen M. Yao Q. . (2019a). Culture-dependent and -independent analyses reveal the diversity, structure, and assembly mechanism of benthic bacterial community in the Ross Sea, Antarctica. Front. Microbiol. 10:2523. doi: 10.3389/fmicb.2019.02523, 31787942 PMC6856632

[ref127] Li W. O’Neill K. R. Haft D. H. DiCuccio M. Chetvernin V. Badretdin A. . (2021). RefSeq: expanding the prokaryotic genome annotation pipeline reach with protein family model curation. Nucleic Acids Res. 49, D1020–D1028. doi: 10.1093/nar/gkaa1105, 33270901 PMC7779008

[ref128] Li H. Yang J. Kuang S. Fu H. Lin H. Peng B. (2025). Magnesium modulates phospholipid metabolism to promote bacterial phenotypic resistance to antibiotics. eLife 13:RP100427. doi: 10.7554/eLife.100427.3 39745871 PMC11695056

[ref129] Li M. Zhu X. Wilkinson S. Huang M. Achal V. (2016). Complete genome sequence of carbonic anhydrase producing *Psychrobacter* sp. SHUES1. Front. Microbiol. 7:1442. doi: 10.3389/fmicb.2016.01442, 27679619 PMC5021049

[ref130] Lo Giudice A. Brilli M. Bruni V. De Domenico M. Fani R. Michaud L. (2007). Bacterium–bacterium inhibitory interactions among psychrotrophic bacteria isolated from Antarctic seawater (Terra Nova Bay, Ross Sea): antagonism among psychrotrophic Antarctic marine bacteria. FEMS Microbiol. Ecol. 60, 383–396. doi: 10.1111/j.1574-6941.2007.00300.x, 17386035

[ref131] Lo Giudice A. Bruni V. Michaud L. (2007a). Characterization of Antarctic psychrotrophic bacteria with antibacterial activities against terrestrial microorganisms. J. Basic Microbiol. 47, 496–505. doi: 10.1002/jobm.200700227, 18072250

[ref132] Lopatina A. Medvedeva S. Shmakov S. Logacheva M. D. Krylenkov V. Severinov K. (2016). Metagenomic analysis of bacterial communities of Antarctic surface snow. Front. Microbiol. 7:398. doi: 10.3389/fmicb.2016.00398, 27064693 PMC4814470

[ref133] Ma Q. Wood T. K. (2011). Protein acetylation in prokaryotes increases stress resistance. Biochem. Biophys. Res. Commun. 410, 846–851. doi: 10.1016/j.bbrc.2011.06.076, 21703240 PMC3138907

[ref134] Magiorakos A.-P. Srinivasan A. Carey R. B. Carmeli Y. Falagas M. E. Giske C. G. . (2012). Multidrug-resistant, extensively drug-resistant and pandrug-resistant bacteria: an international expert proposal for interim standard definitions for acquired resistance. Clin. Microbiol. Infect. 18, 268–281. doi: 10.1111/j.1469-0691.2011.03570.x, 21793988

[ref135] Mallik D. Jain D. Bhakta S. Ghosh A. S. (2022). Role of AmpC-inducing genes in modulating other serine Beta-lactamases in *Escherichia coli*. Antibiotics 11:67. doi: 10.3390/antibiotics11010067, 35052944 PMC8772759

[ref136] Manik M. R. K. Mishu I. D. Mahmud Z. Muskan M. N. Emon S. Z. (2025). Association of fluoroquinolone resistance with rare quinolone resistance-determining region (QRDR) mutations and protein-quinolone binding affinity (PQBA) in multidrug-resistant *Escherichia coli* isolated from patients with urinary tract infection. J. Infect. Public Health 18:102766. doi: 10.1016/j.jiph.2025.102766, 40153979

[ref137] Manzulli V. Schiavone A. Castellana S. Albenzio M. Cafiero M. A. Camarda A. . (2024). *Psychrobacter raelei* sp. nov., isolated from a dog with peritonitis. Int. J. Syst. Evol. Microbiol. 74:10. doi: 10.1099/ijsem.0.006353 38683659

[ref138] Margesin R. Miteva V. (2011). Diversity and ecology of psychrophilic microorganisms. Res. Microbiol. 162, 346–361. doi: 10.1016/j.resmic.2010.12.004, 21187146

[ref139] Martak D. Henriot C. P. Hocquet D. (2024). Environment, animals, and food as reservoirs of antibiotic-resistant bacteria for humans: one health or more? Infect. Dis. 54:104895. doi: 10.1016/j.idnow.2024.104895, 38548016

[ref140] Martins J. M. Scheffer M. C. De Melo Machado H. Schörner M. A. Golfetto L. Santos T. M. D. . (2022). Spectinomycin, gentamicin, and routine disc diffusion testing: an alternative for the treatment and monitoring of multidrug-resistant *Neisseria gonorrhoeae*? J. Microbiol. Methods 197:106480. doi: 10.1016/j.mimet.2022.106480 35526670

[ref141] Maruyama A. Honda D. Yamamoto H. Kitamura K. Higashihara T. (2000). Phylogenetic analysis of psychrophilic bacteria isolated from the Japan trench, including a description of the deep-sea species *Psychrobacter pacificensis* sp. nov. Int. J. Syst. Evol. Microbiol. 50, 835–846. doi: 10.1099/00207713-50-2-835, 10758895

[ref142] Matuschek E. Brown D. F. J. Kahlmeter G. (2014). Development of the EUCAST disk diffusion antimicrobial susceptibility testing method and its implementation in routine microbiology laboratories. Clin. Microbiol. Infect. 20, O255–O266. doi: 10.1111/1469-0691.12373, 24131428

[ref143] McCann C. M. Wade M. J. Gray N. D. Roberts J. A. Hubert C. R. J. Graham D. W. (2016). Microbial communities in a high Arctic Polar Desert landscape. Front. Microbiol. 7:419. doi: 10.3389/fmicb.2016.00419, 27065980 PMC4814466

[ref144] McNeil M. B. Clulow J. S. Wilf N. M. Salmond G. P. C. Fineran P. C. (2012). SdhE is a conserved protein required for Flavinylation of succinate dehydrogenase in Bacteria. J. Biol. Chem. 287, 18418–18428. doi: 10.1074/jbc.M111.293803, 22474332 PMC3365757

[ref145] Meng M. Li Y. Yao H. (2022). Plasmid-mediated transfer of antibiotic resistance genes in soil. Antibiotics 11:525. doi: 10.3390/antibiotics11040525, 35453275 PMC9024699

[ref146] Microsoft Corporation, Excel (2024). Available online at: https://www.microsoft.com/en-us/microsoft-365/excel#:~:text=Microsoft%20Excel%20with%20a%20Microsoft,Excel%2020072C%20and%20Excel%202003 (Accessed August 10, 2025).

[ref147] Miteva V. Teacher C. Sowers T. Brenchley J. (2009). Comparison of the microbial diversity at different depths of the GISP2 Greenland ice core in relationship to deposition climates. Environ. Microbiol. 11, 640–656. doi: 10.1111/j.1462-2920.2008.01835.x, 19278450

[ref148] Mogrovejo D. C. Perini L. Gostinčar C. Sepčić K. Turk M. Ambrožič-Avguštin J. . (2020). Prevalence of antimicrobial resistance and hemolytic phenotypes in Culturable Arctic Bacteria. Front. Microbiol. 11:570. doi: 10.3389/fmicb.2020.00570, 32318045 PMC7147505

[ref149] Mondal A. H. Khare K. Saxena P. Debnath P. Mukhopadhyay K. Yadav D. (2024). A review on Colistin resistance: An antibiotic of last resort. Microorganisms 12:772. doi: 10.3390/microorganisms12040772, 38674716 PMC11051878

[ref150] Mondini A. Anwar M. Z. Ellegaard-Jensen L. Lavin P. Jacobsen C. S. Purcarea C. (2022). Heat shock response of the active microbiome from perennial cave ice. Front. Microbiol. 12:809076. doi: 10.3389/fmicb.2021.809076, 35360653 PMC8960993

[ref151] Mondini A. Donhauser J. Itcus C. Marin C. Perșoiu A. Lavin P. . (2019). High-throughput sequencing of fungal communities across the perennial ice block of Scărișoara ice cave. Ann. Glaciol. 59, 134–146. doi: 10.1017/aog.2019.6

[ref152] Mora-Ochomogo M. Lohans C. T. (2021). β-Lactam antibiotic targets and resistance mechanisms: from covalent inhibitors to substrates. RSC Med. Chem. 12, 1623–1639. doi: 10.1039/D1MD00200G, 34778765 PMC8528271

[ref153] Morita R. Y. (1975). Psychrophilic bacteria. Bacteriol. Rev. 39, 144–167. doi: 10.1128/br.39.2.144-167.1975, 1095004 PMC413900

[ref154] Morozova O. V. Andreeva I. S. Zhirakovskiy V. Y. Pechurkina N. I. Puchkova L. I. Saranina I. V. . (2022). Antibiotic resistance and cold-adaptive enzymes of antarctic culturable bacteria from King George Island. Pol. Sci. 31:100756. doi: 10.1016/j.polar.2021.100756

[ref155] Munita J. M. Arias C. A. (2016). Mechanisms of antibiotic resistance. Microbiol Spectr, 4: VMBF-0016-2015, 4. doi: 10.1128/microbiolspec.VMBF-0016-2015, 27227291 PMC4888801

[ref156] Murray A. E. Kenig F. Fritsen C. H. McKay C. P. Cawley K. M. Edwards R. . (2012). Microbial life at −13 °C in the brine of an ice-sealed Antarctic lake. Proc. Natl. Acad. Sci. USA 109, 20626–20631. doi: 10.1073/pnas.1208607109, 23185006 PMC3528574

[ref157] Musilova M. Tranter M. Bennett S. A. Wadham J. Anesio A. M. (2015). Stable microbial community composition on the Greenland ice sheet. Front. Microbiol. 6:193. doi: 10.3389/fmicb.2015.00193, 25852658 PMC4367435

[ref158] Muziasari W. I. Managaki S. Pärnänen K. Karkman A. Lyra C. Tamminen M. . (2014). Sulphonamide and trimethoprim resistance genes persist in sediments at Baltic Sea aquaculture farms but are not detected in the surrounding environment. PLoS One 9:e92702. doi: 10.1371/journal.pone.0092702, 24651770 PMC3961581

[ref159] Naderi G. Talebi M. Gheybizadeh R. Seifi A. Ghourchian S. Rahbar M. . (2023). Mobile genetic elements carrying aminoglycoside resistance genes in *Acinetobacter baumannii* isolates belonging to global clone 2. Front. Microbiol. 14:1172861. doi: 10.3389/fmicb.2023.1172861, 37213517 PMC10196456

[ref160] Naghavi M. Vollset S. E. Ikuta K. S. Swetschinski L. R. Gray A. P. Wool E. E. . (2024). Global burden of bacterial antimicrobial resistance 1990–2021: a systematic analysis with forecasts to 2050. Lancet 404, 1199–1226. doi: 10.1016/S0140-6736(24)01867-1, 39299261 PMC11718157

[ref161] Nechifor M. Luca C. M. Gales C. (2024). Interactions of antibacterial antibiotics with magnesium and zinc. ijirms 9, 50–58. doi: 10.23958/ijirms/vol09-i01/1798

[ref162] Nedialkova D. Naidenova M. (2005). Screening the antimicrobial activity of Actinomycetes strains isolated from Antarctica. J. Cult. Collect. 4, 29–35.

[ref163] Nishizawa T. Aldrich C. C. Sherman D. H. (2005). Molecular analysis of the Rebeccamycin l -amino acid oxidase from *Lechevalieria aerocolonigenes* ATCC 39243. J. Bacteriol. 187, 2084–2092. doi: 10.1128/JB.187.6.2084-2092.2005, 15743957 PMC1064027

[ref164] Nowak A. Piotrowska M. (2012). Biochemical activities of *Brochothrix thermosphacta*. Meat Sci. 90, 410–413. doi: 10.1016/j.meatsci.2011.08.008, 21914560

[ref165] Nunez-Montero K. Barrientos L. (2018). Advances in Antarctic research for antimicrobial discovery: A comprehensive narrative review of Bacteria from Antarctic environments as potential sources of novel antibiotic compounds against human pathogens and microorganisms of industrial importance. Antibiotics 7:90. doi: 10.3390/antibiotics7040090, 30347637 PMC6316688

[ref166] O’Brien A. Sharp R. Russell N. J. Roller S. (2004). Antarctic bacteria inhibit growth of food-borne microorganisms at low temperatures. FEMS Microbiol. Ecol. 48, 157–167. doi: 10.1016/j.femsec.2004.01.001, 19712399

[ref167] Ogorek R. Borzęcka J. Spychała K. Piecuch A. Suchodolski J. (2024). Soil and sediments in natural underground ecosystems as a source of culturable micromycetes: a case study of the Brestovská cave (Western Tatras, Slovakia). Appl. Sci. 14:3517. doi: 10.3390/app14083517

[ref168] Oman T. J. Boettcher J. M. Wang H. Okalibe X. N. Van Der Donk W. A. (2011). Sublancin is not a lantibiotic but an S-linked glycopeptide. Nat. Chem. Biol. 7, 78–80. doi: 10.1038/nchembio.509, 21196935 PMC3060661

[ref169] Omote H. Hiasa M. Matsumoto T. Otsuka M. Moriyama Y. (2006). The MATE proteins as fundamental transporters of metabolic and xenobiotic organic cations. Trends Pharmacol. Sci. 27, 587–593. doi: 10.1016/j.tips.2006.09.001, 16996621

[ref170] Ozturk R. C. Feyzioglu A. M. Altinok I. (2022). Prokaryotic community and diversity in coastal surface waters along the Western Antarctic peninsula. Pol. Sci. 31:100764. doi: 10.1016/j.polar.2021.100764

[ref171] Pal C. Asiani K. Arya S. Rensing C. Stekel D. J. Larsson D. G. J. al. (2017). “Metal resistance and its association with antibiotic resistance,” in Advances in Microbial Physiology. (Oxford, United Kingdom: Elsevier), 261–313. 10.1016/bs.ampbs.2017.02.001 28528649

[ref172] Pan X. Xu L. Li Y. Wu S. Wu Y. Wei W. (2022). Strategies to improve the biosynthesis of β-lactam antibiotics by penicillin G Acylase: Progress and prospects. Front. Bioeng. Biotechnol. 10:936487. doi: 10.3389/fbioe.2022.936487, 35923572 PMC9340067

[ref173] Parks D. H. Chuvochina M. Waite D. W. Rinke C. Skarshewski A. Chaumeil P.-A. . (2018). A standardized bacterial taxonomy based on genome phylogeny substantially revises the tree of life. Nat. Biotechnol. 36, 996–1004. doi: 10.1038/nbt.4229, 30148503

[ref174] Parte A. C. Sardà Carbasse J. Meier-Kolthoff J. P. Reimer L. C. Göker M. (2020). List of prokaryotic names with standing in nomenclature (LPSN) moves to the DSMZ. Int. J. Syst. Evol. Microbiol. 70, 5607–5612. doi: 10.1099/ijsem.0.004332, 32701423 PMC7723251

[ref175] Paun V. I. Icaza G. Lavin P. Marin C. Tudorache A. Perşoiu A. . (2019). Total and potentially active bacterial communities entrapped in a late glacial through holocene ice core from Scarisoara ice cave, Romania. Front. Microbiol. 10:1193. doi: 10.3389/fmicb.2019.01193, 31244788 PMC6563852

[ref176] Paun V. I. Ion S. G. Gheorghita G. R. Podolean I. Tudorache M. Purcarea C. (2024). Cold-active lipase from the ice cave Psychrobacter SC65A.3 strain, a promising biocatalyst for Silybin acylation. Molecules 29:5125. doi: 10.3390/molecules29215125, 39519766 PMC11547725

[ref177] Paun V. I. Lavin P. Chifiriuc M. C. Purcarea C. (2021). First report on antibiotic resistance and antimicrobial activity of bacterial isolates from 13,000-year old cave ice core. Sci. Rep. 11:514. doi: 10.1038/s41598-020-79754-5, 33436712 PMC7804186

[ref178] Pérez-Pérez F. J. Hanson N. D. (2002). Detection of plasmid-mediated AmpC β-lactamase genes in clinical isolates by using multiplex PCR. J. Clin. Microbiol. 40, 2153–2162. doi: 10.1128/JCM.40.6.2153-2162.2002, 12037080 PMC130804

[ref179] Perkins A. E. Nicholson W. L. (2008). Uncovering new metabolic capabilities of *Bacillus subtilis* using phenotype profiling of rifampin-resistant rpoB mutants. J. Bacteriol. 190, 807–814. doi: 10.1128/JB.00901-07, 17644585 PMC2223569

[ref180] Perry J. Waglechner N. Wright G. (2016). The prehistory of antibiotic resistance. Cold Spring Harb. Perspect. Med. 6:a025197. doi: 10.1101/cshperspect.a025197, 27252395 PMC4888810

[ref181] Persoiu A. Onac B. P. Wynn J. G. Blaauw M. Ionita M. Hansson M. (2017). Holocene winter climate variability in central and Eastern Europe. Sci. Rep. 7:1196. doi: 10.1038/s41598-017-01397-w, 28446780 PMC5430645

[ref182] Persoiu A. Pazdur A. (2010). Ice genesis and its long-term mass balance and dynamics in Scărişoara ice cave, Romania. Cryosphere 5, 45–53. doi: 10.5194/tc-5-45-2011

[ref183] Petrova M. Gorlenko Z. Mindlin S. (2009). Molecular structure and translocation of a multiple antibiotic resistance region of a *Psychrobacter psychrophilus* permafrost strain. FEMS Microbiol. Lett. 296, 190–197. doi: 10.1111/j.1574-6968.2009.01635.x, 19459955

[ref184] Popovic B. Li Y. Chirgadze D. Y. Blundell T. L. Spencer J. B. (2006). Structural characterisation of BtrK decarboxylase from butirosin biosynthesis. PDB - Protein Data Bank. doi: 10.2210/pdb2j66/pdb

[ref185] Priscu J. C. Adams E. E. Lyons W. B. Voytek M. A. Mogk D. W. Brown R. L. . (1999). Geomicrobiology of subglacial ice above Lake Vostok, Antarctica. Science 286, 2141–2144. doi: 10.1126/science.286.5447.2141, 10591642

[ref186] Priscu J. C. Fritsen C. H. Adams E. E. Giovannoni S. J. Paerl H. W. McKay C. P. . (1998). Perennial Antarctic Lake ice: An oasis for life in a Polar Desert. Science 280, 2095–2098. doi: 10.1126/science.280.5372.2095, 9641910

[ref187] Purcarea C. (2018). “Microbial life in ice caves” in Ice Caves. (Amsterdam, Netherlands: Elsevier), 173–187.

[ref188] Python Software Foundation (2023). Available online at: https://www.python.org/psf-landing/ (Accessed August 10, 2025).

[ref189] Redgrave L. S. Sutton S. B. Webber M. A. Piddock L. J. V. (2014). Fluoroquinolone resistance: mechanisms, impact on bacteria, and role in evolutionary success. Trends Microbiol. 22, 438–445. doi: 10.1016/j.tim.2014.04.007, 24842194

[ref190] Rehakova K. Stibal M. Šabacká M. Řehák J. (2010). Survival and colonisation potential of photoautotrophic microorganisms within a glacierised catchment on Svalbard, high Arctic. Polar Biol. 33, 737–745. doi: 10.1007/s00300-009-0751-x

[ref191] Reygaert W. C. (2018). An overview of the antimicrobial resistance mechanisms of bacteria. AIMS Microbiology 4, 482–501. doi: 10.3934/microbiol.2018.3.482, 31294229 PMC6604941

[ref192] Rivkina E. Petrovskaya L. Vishnivetskaya T. Krivushin K. Shmakova L. Tutukina M. . (2016). Metagenomic analyses of the late Pleistocene permafrost – additional tools for reconstruction of environmental conditions. Biogeosciences 13, 2207–2219. doi: 10.5194/bg-13-2207-2016

[ref193] Roberts M. C. (2005). Update on acquired tetracycline resistance genes. FEMS Microbiol. Lett. 245, 195–203. doi: 10.1016/j.femsle.2005.02.034, 15837373

[ref194] Rodrigues D. F. da C Jesus E. Ayala-del-Río H. L. Pellizari V. H. Gilichinsky D. Sepulveda-Torres L. . (2009). Biogeography of two cold-adapted genera: *Psychrobacter* and *Exiguobacterium*. ISME J. 3, 658–665. doi: 10.1038/ismej.2009.25 19322243

[ref195] Rojas J. L. Martín J. Tormo J. R. Vicente F. Brunati M. Ciciliato I. . (2009). Bacterial diversity from benthic mats of Antarctic lakes as a source of new bioactive metabolites. Mar. Genomics 2, 33–41. doi: 10.1016/j.margen.2009.03.005, 21798170

[ref196] Romanenko L. A. Lysenko A. M. Rohde M. Mikhailov V. V. Stackebrandt E. (2004). *Psychrobacter maritimus* sp. nov. and *Psychrobacter arenosus* sp. nov., isolated from coastal sea ice and sediments of the sea of Japan. Int. J. Syst. Evol. Microbiol. 54, 1741–1745. doi: 10.1099/ijs.0.63096-0, 15388738

[ref197] Romanenko L. A. Schumann P. Rohde M. Lysenko A. M. Mikhailov V. V. Stackebrandt E. (2002). *Psychrobacter submarinus* sp. nov. and *Psychrobacter marincola* sp. nov., psychrophilic halophiles from marine environments. Int. J. Syst. Evol. Microbiol. 52, 1291–1297. doi: 10.1099/00207713-52-4-1291, 12148642

[ref198] Rome K. Borde C. Taher R. Cayron J. Lesterlin C. Gueguen E. . (2018). The two-component system ZraPSR is a novel ESR that contributes to intrinsic antibiotic tolerance in *Escherichia coli*. J. Mol. Biol. 430, 4971–4985. doi: 10.1016/j.jmb.2018.10.021, 30389436

[ref199] Rothschild L. J. Mancinelli R. L. (2001). Life in extreme environments. Nature 409, 1092–1101. doi: 10.1038/35059215 11234023

[ref200] Sakalauskienė G. V. Malcienė L. Stankevičius E. Radzevičienė A. (2025). Unseen enemy: mechanisms of multidrug antimicrobial resistance in Gram-negative ESKAPE pathogens. Antibiotics 14:63. doi: 10.3390/antibiotics14010063, 39858349 PMC11762671

[ref201] Salazar-Hamm P. S. Homan F. E. Good S. A. Hathaway J. J. M. Clements A. E. Haugh E. G. . (2025). Subterranean marvels: microbial communities in caves and underground mines and their promise for natural product discovery. Nat. Prod. Rep. 42, 592–622. doi: 10.1039/D4NP00055B, 39950737

[ref202] Sanchez L. A. Gómez F. F. Delgado O. D. (2009). Cold-adapted microorganisms as a source of new antimicrobials. Extremophiles 13, 111–120. doi: 10.1007/s00792-008-0203-5, 19015813

[ref203] Sanchez-Corona C. G. Gonzalez-Avila L. U. Hernández-Cortez C. Rojas-Vargas J. Castro-Escarpulli G. Castelán-Sánchez H. G. (2025). Impact of heavy metal and resistance genes on antimicrobial resistance: ecological and public health implications. Genes 16:625. doi: 10.3390/genes16060625, 40565518 PMC12191737

[ref204] Schoch C. L. Ciufo S. Domrachev M. Hotton C. L. Kannan S. Khovanskaya R. . (2020). NCBI taxonomy: a comprehensive update on curation, resources and tools. Database 2020:baaa062. doi: 10.1093/database/baaa062, 32761142 PMC7408187

[ref205] Serio A. W. Magalhães M. L. Blanchard J. S. Connolly L. E. (2017). “Aminoglycosides: mechanisms of action and resistance” in Antimicrobial Drug Resistance. eds. Mayers D. L. Sobel J. D. Ouellette M. Kaye K. S. Marchaim D. (Cham: Springer International Publishing), 213–229.

[ref206] Shang D.-D. Yang G.-J. Chen G.-J. Du Z.-J. (2022). Psychrobacter halodurans sp. nov. and Psychrobacter coccoides sp. nov., two new slightly halophilic bacteria isolated from marine sediment. Int. J. Syst. Evol. Microbiol. 72:10. doi: 10.1099/ijsem.0.005173, 35037845

[ref207] Sharma R. C. Kumar R. (2017). Water quality assessment of sacred glacial Lake Satopanth of Garhwal Himalaya, India. Appl Water Sci 7, 4757–4764. doi: 10.1007/s13201-017-0638-x

[ref208] Shcherbakova V. A. Chuvil’skaia N. A. Rivkina E. M. Pecheritsyna S. A. Suetin S. V. Laurinavichius K. S. . (2009). Novel halotolerant bacterium from cryopeg in permafrost: description of *Psychrobacter muriicola* sp. nov. Mikrobiologiia 78, 98–105. doi: 10.1134/S0026261709010111 19334602

[ref209] Shekh R. M. Singh P. Singh S. M. Roy U. (2011). Antifungal activity of Arctic and Antarctic bacteria isolates. Polar Biol. 34, 139–143. doi: 10.1007/s00300-010-0854-4

[ref210] Shivaji S. Reddy G. S. N. Raghavan P. U. M. Sarita N. B. Delille D. (2004). *Psychrobacter salsus* sp. nov. and *Psychrobacter adeliensis* sp. nov. isolated from fast ice from Adelie Land. Antarctica. Systematic and Applied Microbiology 27, 628–635. doi: 10.1078/0723202042369956, 15612619

[ref211] Shivaji S. Reddy G. S. N. Suresh K. Gupta P. Chintalapati S. Schumann P. . (2005). *Psychrobacter vallis* sp. nov. and *Psychrobacter aquaticus* sp. nov., from Antarctica. Int. J. Syst. Evol. Microbiol. 55, 757–762. doi: 10.1099/ijs.0.03030-0, 15774658

[ref212] Silva T. R. Duarte A. W. F. Passarini M. R. Z. Ruiz A. L. T. G. Franco C. H. Moraes C. B. . (2018). Bacteria from Antarctic environments: diversity and detection of antimicrobial, antiproliferative, and antiparasitic activities. Polar Biol. 41, 1505–1519. doi: 10.1007/s00300-018-2300-y

[ref213] Song M. Song D. Jiang L. Zhang D. Sun Y. Chen G. . (2021). Large-scale biogeographical patterns of antibiotic resistome in the forest soils across China. J. Hazard. Mater. 403:123990. doi: 10.1016/j.jhazmat.2020.123990, 33265028

[ref214] Staloch B. E. K. Niero H. de Freitas R. C. Ballone P. Rodrigues-Costa F. Trivella D. B. B. . (2022). Draft genome sequence of *Psychrobacter nivimaris* LAMA 639 and its biotechnological potential. Data Brief 41:107927. doi: 10.1016/j.dib.2022.107927, 35242911 PMC8857425

[ref215] Sudzinová P. Šanderová H. Koval’ T. Skálová T. Borah N. Hnilicová J. . (2023). What the Hel: recent advances in understanding rifampicin resistance in bacteria. FEMS Microbiol. Rev. 47:fuac051. doi: 10.1093/femsre/fuac051 36549665 PMC10719064

[ref216] Tam H. K. Wong C. M. V. L. Yong S. T. Blamey J. González M. (2015). Multiple-antibiotic-resistant bacteria from the maritime Antarctic. Polar Biol. 38, 1129–1141. doi: 10.1007/s00300-015-1671-6

[ref217] Tatusova T. DiCuccio M. Badretdin A. Chetvernin V. Nawrocki E. P. Zaslavsky L. . (2016). NCBI prokaryotic genome annotation pipeline. Nucleic Acids Res. 44, 6614–6624. doi: 10.1093/nar/gkw569, 27342282 PMC5001611

[ref218] The European Committee on Antimicrobial Susceptibility Testing 2020 Breakpoint Tables for Interpretation of MICs and Zone Diameters. Version 10.0 Available online at: http://www.eucast.org (Accessed September 12, 2020).

[ref219] Thi T. D. Lopez E. Rodriguez-Rojas A. Rodriguez-Beltran J. Couce A. Guelfo J. R. . (2011). Effect of recA inactivation on mutagenesis of *Escherichia coli* exposed to sublethal concentrations of antimicrobials. J. Antimicrob. Chemother. 66, 531–538. doi: 10.1093/jac/dkq496, 21212055

[ref220] Thompson J. D. Higgins D. G. Gibson T. J. (1994). CLUSTAL W: improving the sensitivity of progressive multiple sequence alignment through sequence weighting, position-specific gap penalties and weight matrix choice. Nucl Acids Res 22, 4673–4680. doi: 10.1093/nar/22.22.4673, 7984417 PMC308517

[ref221] UNEP (2023). United Nations Environment Programme - Bracing for Superbugs: Strengthening Environmental Action in the One Health Response to Antimicrobial Resistance. New York: United Nations.

[ref222] Van Goethem M. W. Pierneef R. Bezuidt O. K. I. Van De Peer Y. Cowan D. A. Makhalanyane T. P. (2018). A reservoir of ‘historical’ antibiotic resistance genes in remote pristine Antarctic soils. Microbiome 6:40. doi: 10.1186/s40168-018-0424-5, 29471872 PMC5824556

[ref223] Vela A. I. Collins M. D. Latre M. V. Mateos A. Moreno M. A. Hutson R. . (2003). *Psychrobacter pulmonis* sp. nov., isolated from the lungs of lambs. Int. J. Syst. Evol. Microbiol. 53, 415–419. doi: 10.1099/ijs.0.02413-0, 12710606

[ref224] Ventosa A. Nieto J. J. Oren A. (1998). Biology of moderately halophilic aerobic Bacteria. Microbiol. Mol. Biol. Rev. 62, 504–544. doi: 10.1128/MMBR.62.2.504-544.1998, 9618450 PMC98923

[ref225] Vester B. Douthwaite S. (2001). Macrolide resistance conferred by base substitutions in 23S rRNA. Antimicrob. Agents Chemother. 45, 1–12. doi: 10.1128/AAC.45.1.1-12.2001, 11120937 PMC90232

[ref226] Walker B. J. Abeel T. Shea T. Priest M. Abouelliel A. Sakthikumar S. . (2014). Pilon: An integrated tool for comprehensive microbial variant detection and genome assembly improvement. PLoS One 9:e112963. doi: 10.1371/journal.pone.0112963, 25409509 PMC4237348

[ref227] Wang Z. Li Y. Lin X. (2017). Transcriptome analysis of the Antarctic psychrotrophic bacterium *Psychrobacter* sp. G in response to temperature stress. Acta Oceanol. Sin. 36, 78–87. doi: 10.1007/s13131-016-0956-0

[ref228] Wang N. F. Zhang T. Yang X. Wang S. Yu Y. Dong L. L. . (2016). Diversity and composition of bacterial Community in Soils and Lake Sediments from an Arctic Lake area. Front. Microbiol. 7:1170. doi: 10.3389/fmicb.2016.01170, 27516761 PMC4963411

[ref229] Welter D. K. Ruaud A. Henseler Z. M. De Jong H. N. Van Coeverden De Groot P. Michaux J. . (2021). Free-living, psychrotrophic bacteria of the genus *Psychrobacter* are descendants of pathobionts. mSystems 6:10. doi: 10.1128/msystems.00258-21 PMC8546975 33850039

[ref230] Wietz M. Månsson M. Bowman J. S. Blom N. Ng Y. Gram L. (2012). Wide distribution of closely related, antibiotic-producing Arthrobacter strains throughout the Arctic Ocean. Appl. Environ. Microbiol. 78, 2039–2042. doi: 10.1128/AEM.07096-11, 22247128 PMC3298137

[ref231] Willms I. M. Kamran A. Aßmann N. F. Krone D. Bolz S. H. Fiedler F. . (2019). Discovery of novel antibiotic resistance determinants in Forest and grassland soil metagenomes. Front. Microbiol. 10:460. doi: 10.3389/fmicb.2019.00460, 30899254 PMC6416219

[ref232] Wing S. R. McLeod R. J. Leichter J. J. Frew R. D. Lamare M. D. (2012). Sea ice microbial production supports Ross Sea benthic communities: influence of a small but stable subsidy. Ecology 93, 314–323. doi: 10.1890/11-0996.1, 22624313

[ref233] Wirth S. E. Ayala-del-Río H. L. Cole J. A. Kohlerschmidt D. J. Musser K. A. Sepúlveda-Torres L. d. C. . (2012). *Psychrobacter sanguinis* sp. nov., recovered from four clinical specimens over a 4-year period. Int. J. Syst. Evol. Microbiol. 62, 49–54. doi: 10.1099/ijs.0.029058-0, 21317274

[ref234] Wong C. Tam H. Alias S. Gonzalez M. Gonzalez-Rocha G. Dominguez-Yevenes M. (2011). *Pseudomonas* and *Pedobacter* isolates from King George Island inhibited the growth of foodborne pathogens. Pol Polar Res 32, 3–14. doi: 10.2478/v10183-011-0003-y

[ref235] Wu X. Flatt P. M. Xu H. Mahmud T. (2009). Biosynthetic gene cluster of Cetoniacytone A, an unusual aminocyclitol from the endosymbiotic bacterium *Actinomyces* sp. Lu 9419. Chembiochem 10, 304–314. doi: 10.1002/cbic.200800527, 19101977 PMC3136446

[ref236] Xue Y. Zhao L. Liu H. Sherman D. H. (1998). A gene cluster for macrolide antibiotic biosynthesis in *Streptomyces venezuelae*: architecture of metabolic diversity. Proc. Natl. Acad. Sci. USA 95, 12111–12116. doi: 10.1073/pnas.95.21.12111, 9770448 PMC22793

[ref237] Yadav A. N. Verma P. Kumar V. Sachan S. Saxena A. (2017). Extreme cold environments: a suitable niche for selection of novel psychrotrophic microbes for biotechnological applications. AIBM, 2:555584. doi: 10.19080/AIBM.2017.02.555584

[ref238] Yang X. (2014). “Moraxellaceae” in Encyclopedia of Food Microbiology (Amsterdam: Elsevier), 826–833.

[ref239] Yoon S.-H. Ha S. Lim J. Kwon S. Chun J. (2017). A large-scale evaluation of algorithms to calculate average nucleotide identity. Antonie Van Leeuwenhoek 110, 1281–1286. doi: 10.1007/s10482-017-0844-4, 28204908

[ref240] Yoon J.-H. Kang K. H. Park Y.-H. (2003). *Psychrobacter jeotgali* sp. nov., isolated from jeotgal, a traditional Korean fermented seafood. Int. J. Syst. Evol. Microbiol. 53, 449–454. doi: 10.1099/ijs.0.02242-0, 12710611

[ref241] Yoon J.-H. Lee C.-H. Kang S.-J. Oh T.-K. (2005c). *Psychrobacter celer* sp. nov., isolated from sea water of the South Sea in Korea. Int. J. Syst. Evol. Microbiol. 55, 1885–1890. doi: 10.1099/ijs.0.63682-0, 16166683

[ref242] Yoon J.-H. Lee C.-H. Yeo S.-H. Oh T.-K. (2005b). *Psychrobacter aquimaris* sp. nov. and *Psychrobacter namhaensis* sp. nov., isolated from sea water of the South Sea in Korea. Int. J. Syst. Evol. Microbiol. 55, 1007–1013. doi: 10.1099/ijs.0.63464-0, 15879226

[ref243] Yoon J.-H. Yeo S.-H. Oh T.-K. Park Y.-H. (2005a). *Psychrobacter alimentarius* sp. nov., isolated from squid jeotgal, a traditional Korean fermented seafood. Int. J. Syst. Evol. Microbiol. 55, 171–176. doi: 10.1099/ijs.0.63140-0, 15653872

[ref244] Yuan M. Yu Y. Li H.-R. Dong N. Zhang X.-H. (2014). Phylogenetic diversity and biological activity of Actinobacteria isolated from the Chukchi shelf marine sediments in the Arctic Ocean. Mar. Drugs 12, 1281–1297. doi: 10.3390/md12031281, 24663116 PMC3967210

[ref245] Yumoto I. (2003). *Psychrobacter okhotskensis* sp. nov., a lipase-producing facultative psychrophile isolated from the coast of the Okhotsk Sea. Int. J. Syst. Evol. Microbiol. 53, 1985–1989. doi: 10.1099/ijs.0.02686-0, 14657134

[ref246] Yumoto I. Hirota K. Kimoto H. Nodasaka Y. Matsuyama H. Yoshimune K. (2010). *Psychrobacter piscatorii* sp. nov., a psychrotolerant bacterium exhibiting high catalase activity isolated from an oxidative environment. Int. J. Syst. Evol. Microbiol. 60, 205–208. doi: 10.1099/ijs.0.010959-0, 19648327

[ref247] Zachariah S. Kumari P. Das S. K. (2016). Psychrobacter pocilloporae sp. nov., isolated from a coral, *Pocillopora eydouxi*. Int. J. Syst. Evol. Microbiol. 66, 5091–5098. doi: 10.1099/ijsem.0.001476, 27609593

[ref248] Zada S. Sajjad W. Rafiq M. Ali S. Hu Z. Wang H. . (2022). Cave microbes as a potential source of drugs development in the modern era. Microb. Ecol. 84, 676–687. doi: 10.1007/s00248-021-01889-3, 34693460 PMC8542507

[ref249] Zaunbrecher M. A. Sikes R. D. Metchock B. Shinnick T. M. Posey J. E. (2009). Overexpression of the chromosomally encoded aminoglycoside acetyltransferase *eis* confers kanamycin resistance in *Mycobacterium tuberculosis*. Proc. Natl. Acad. Sci. USA 106, 20004–20009. doi: 10.1073/pnas.0907925106, 19906990 PMC2785282

[ref250] Zeng Y.-X. Yu Y. Li H.-R. Luo W. (2015). Psychrobacter fjordensis sp. nov., a psychrotolerant bacterium isolated from an Arctic fjord in Svalbard. Antonie Van Leeuwenhoek 108, 1283–1292. doi: 10.1007/s10482-015-0580-6, 26362329

[ref251] Zeng Y.-X. Yu Y. Liu Y. Li H.-R. (2016). *Psychrobacter glaciei* sp. nov., isolated from the ice core of an Arctic glacier. Int. J. Syst. Evol. Microbiol. 66, 1792–1798. doi: 10.1099/ijsem.0.000939 26827927

[ref252] Zgurskaya H. I. (2002). Molecular analysis of efflux pump-based antibiotic resistance. Int. J. Med. Microbiol. 292, 95–105. doi: 10.1078/1438-4221-00195, 12195740

[ref253] Zhang G. Niu F. Ma X. Liu W. Dong M. Feng H. . (2007). Phylogenetic diversity of bacteria isolates from the Qinghai-Tibet plateau permafrost region. Can. J. Microbiol. 53, 1000–1010. doi: 10.1139/W07-031, 17898857

[ref254] Zhang S. Song W. Yu M. Lin X. (2017). Comparative genomics analysis of five Psychrobacter strains isolated from world-wide habitats reveal high intra-genus variations. Extremophiles 21, 581–589. doi: 10.1007/s00792-017-0927-1, 28314921

