# Supplementary material for: First genome sequence and functional profiling of Psychrobacter SC65A.3 preserved in 5,000-year-old cave ice: insights into ancient resistome, antimicrobial potential, and enzymatic activities
Source: Front Microbiol. 2026 Feb 17;16:1713017. doi: 10.3389/fmicb.2025.1713017 (PMC12953519; doi:10.3389/fmicb.2025.1713017)
Supplement: Supplementary file 1 [file Data_Sheet_1.PDF]

Supplementary Material

First genome sequence and functional profiling of *Psychrobacter* SC65A.3 preserved in 5,000-year-old cave ice: insights into ancient resistome, antimicrobial potential and enzymatic activities

Victoria Ioana Paun[1], Corina Itcus[1], Paris Lavin[2,3], Mariana C. Chifiriuc[4], Cristina Purcarea[1*]

[1] Institute of Biology Bucharest of the Romanian Academy, Department of Microbiology, Romanian Academy, Bucharest, Romania

[2] Facultad de Ciencias del Mar y Recursos Biologicos, Departamento de Biotecnologıa, Universidad de Antofagasta, Antofagasta, Chile

[3] Centro de Investigación en Inmunología y Biotecnología Biomédica de Antofagasta (CIIBBA), Universidad de Antofagasta, Chile

[4] Faculty of Biology and the Research Institute of the University of Bucharest, ICUB, University of Bucharest, Bucharest, Romania

*** Correspondence:** Cristina Purcarea, cristina.purcarea@ibiol.ro

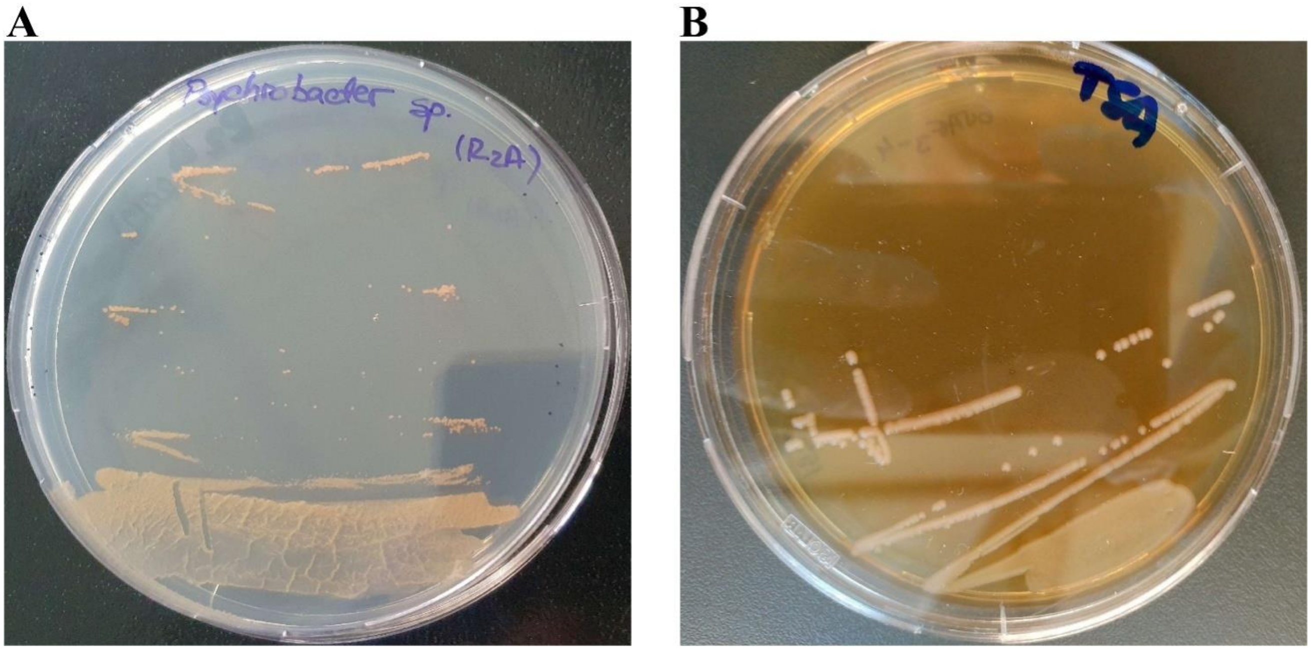

Supplementary Figure S1. Isolation of bacterial strain *Psychrobacter* SC65A.3 on R2A and TSA medium. **(A)** isolated colonies on R2A medium at 4°C; **(B)** isolated colonies on TSA medium at 15°C

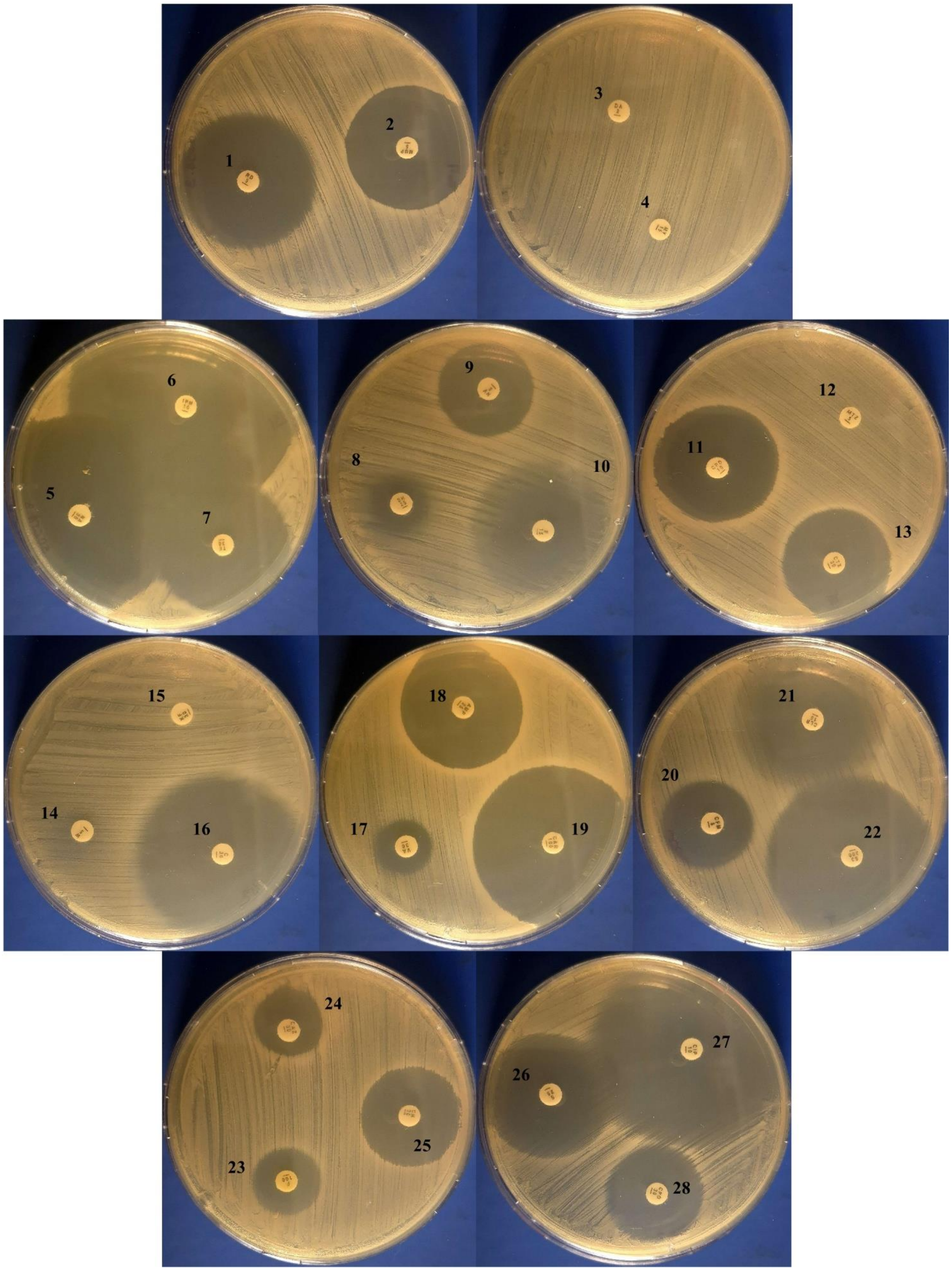

Supplementary Figure S2. Antimicrobial resistance evaluation of Scarisoara strain SC65A.3 against 28 antibiotics (Table 2). (1) RD/5 µg Rifampicin; (2) MUP/5 µg Mupirocin; (3) DA/2 µg Clindamycin; (4) MY/15 µg Lincomycin; (5) NA/30 µg Nalidixic acid; (6) IPM/10 µg Imipenem; (7) TE/30 µg Tetracycline; (8) SH/25 µg Spectinomycin; (9) NV/5 µg Novobiocin; (10) E/15 µg Erythromycin; (11) CPD/10 µg Cefpodoxime; (12) MTZ/5 µg Metronidazole; (13) CTX/30 µg Cefotaxime; (14) W/5 µg Trimethoprim; (15) KF/30 µg Cephalothin; (16) C/30 µg Chloramphenicol; (17) VA/30 µg Vancomycin; (18) AMP/25 µg Ampicillin; (19) CAR/100 µg Carbenicillin; (20) CFM/5 µg Cefixime; (21) CLR/15 µg Clarithromycin; (22) S3/300 µg Sulfonamide compounds; (23) F/100 µg Nitrofurantoin; (24) CAZ/30 µg Ceftazidime; (25) CN/30 µg Gentamicin; (26) S/25 µg Streptomycin; (27) CIP/10 µg Ciprofloxacin; (28) CPO/30 µg Cefpirome.

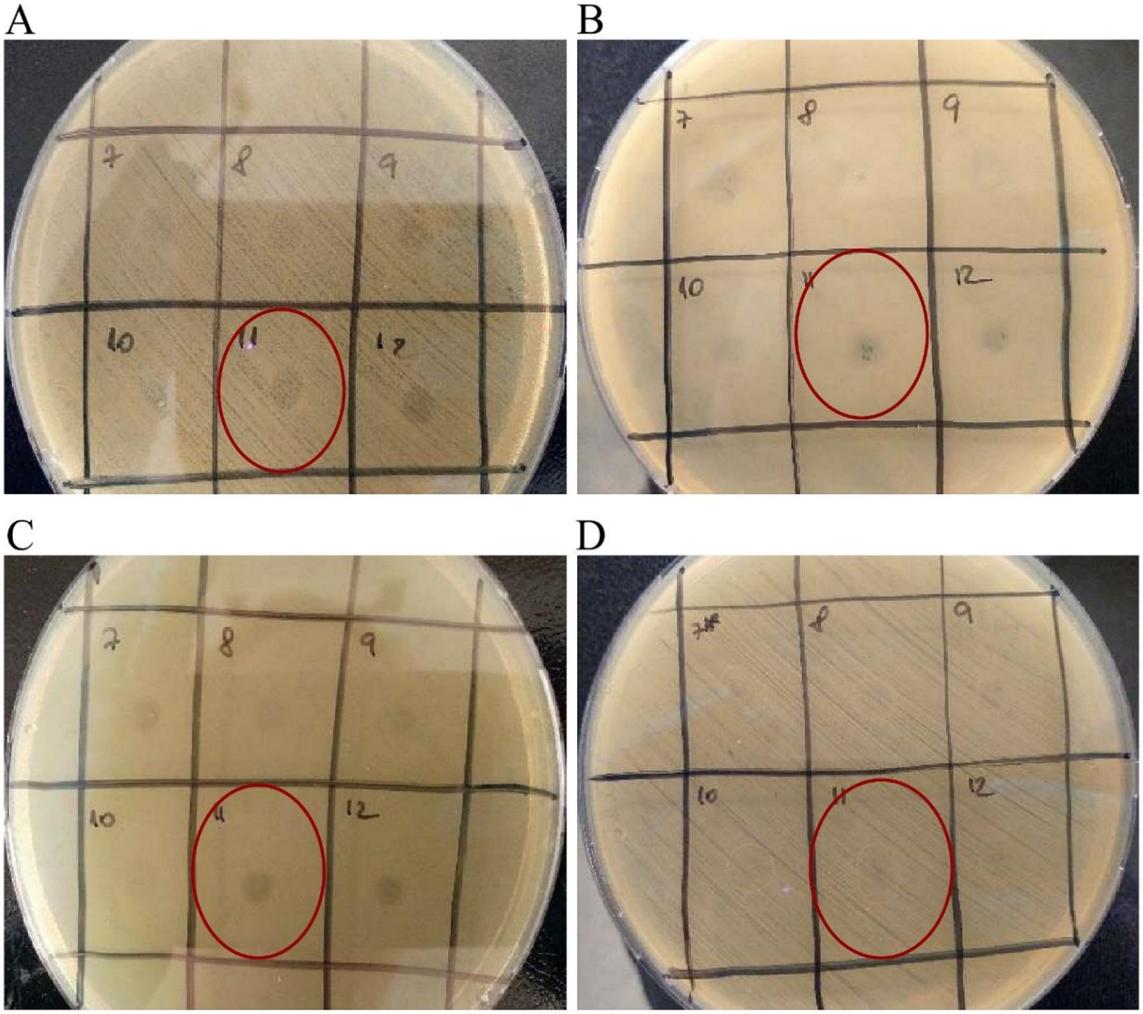

Supplementary Figure S3. Antimicrobial activity evaluation of different Scarisoara strains. *Psychrobacter* SC65A.3 strain (spot 11, red circle). Pathogens: **(A)** *Escherichia coli* ATCC 25922, **(B)** *Enterobacter cloacae* 19069 ONE3, **(C)** *Pseudomonas aeruginosa* 19053 CNE5, **(D)** *Staphylococcus aureus* subsp. aureus Rosenbach ATCC 25923.

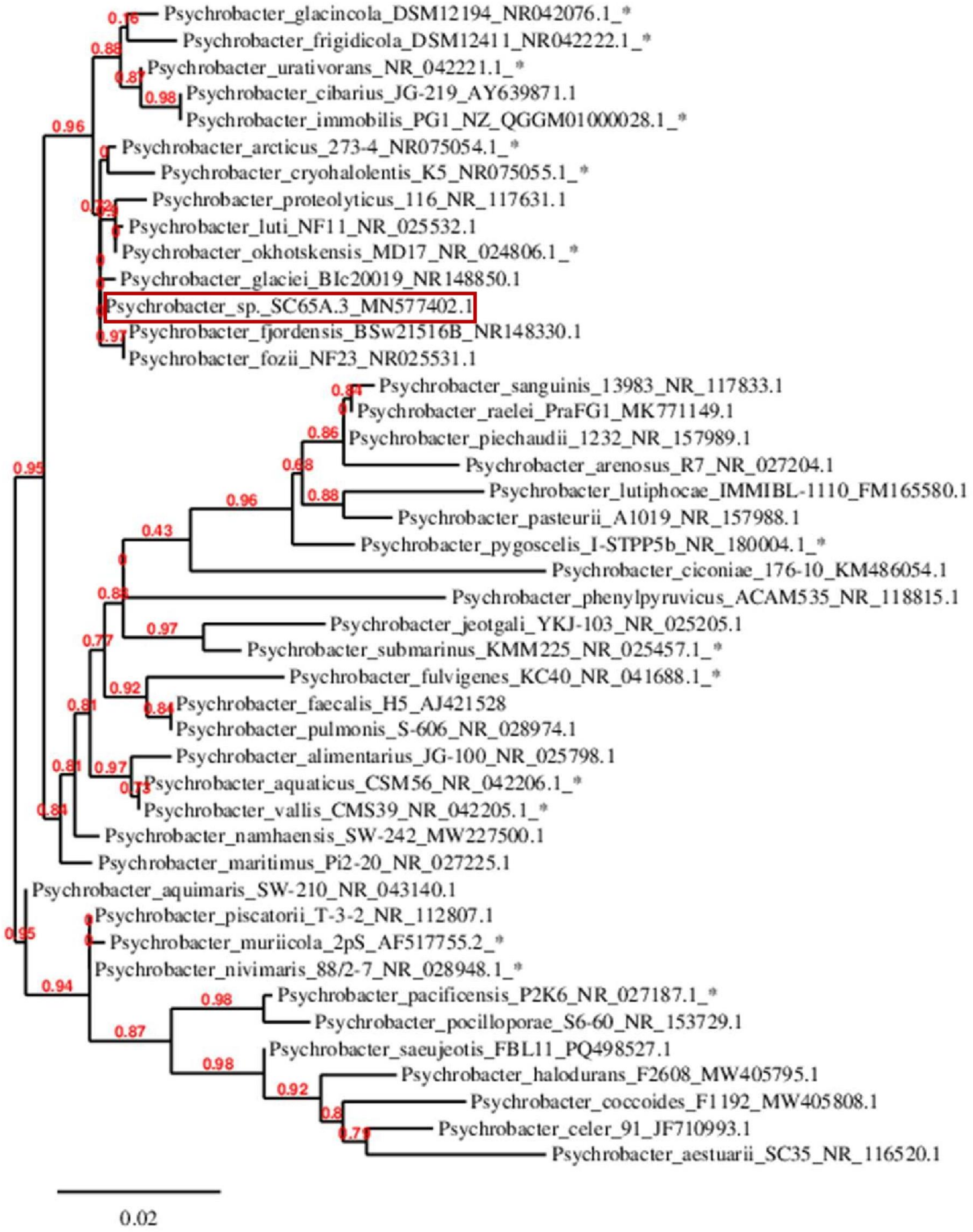

Supplementary Figure S4. Maximum-likelihood tree based on 16S rRNA gene sequences indicating the phylogenetic position of *Psychrobacter* SC65A.3 among 15 psychrophilic (*) and 28 psychrotrophic *Psychrobacter* strains retrieved from NCBI Database. The scale bar represents the number of changes per nucleotide position.

Supplementary Table S1. Whole genome sequencing data for *Psychrobacter* SC65A.3 genome

Illumina sequencing data			
	**Total read bases**	**Total reads**	**GC (%)**
**Raw dataset**	7,538,171,566	49,921,666	42.4
**Filtered dataset**	5,372,583,624	35,580,024	42.49

Homology scores of top 3 Psychcrobacter species						
**Query**		**Subject**			**Score**	
**Name**	**Coverage**	**Description**	**Length**	**Coverage**	**Identity (%)**	**Gap (%)**
**SC65A.3**	3.99	*Psychrobacter cryohalolentis* FDAARGOS_308 9 [CP022043.2]	3,067,424	3.96	97	0
	3.99	*Psychrobacter cryohalolentis* K5 [CP000323.1]	3,059,876	3.97	97	0
	3.64	*Psychrobacter sp*. G [CP006265.1]	3,079,438	3.6	97	0

Genome annotation					
**Contig name**	**Length**	**CDS**	**No. of genes**	**tRNA**	**rRNA**
**SC65A.3**	3,046,103	2,602	2,536	50	15

EggNOG count for Psychrobacter sp. SC65A.3	
**Total Protein**	**2536**
**Eggnog DB Matched Protein**	**2484**
**Single Eggnog**	**2428**
**Multi Eggnog**	**56**
**No hit**	**52**

Supplementary Table S2. Heat-shock and cold-shock associated genes in SC65A3 genome

EggNOG genes (no)	Heat-shock genes (no)	Cold-shock genes (no)
**J:** Translation, ribosomal structure, biogenesis (160)	*hslR, miaA, pnp (3)*	*infB, infC, rbfA (3)*
**K:** Transcription (101)	*mngR, rpoH (2)*	*cspA1, cspA2, cspV, rnr (4)*
**L:** Replication, recombination & repair (145)	-	*crhR (1)*
**T:** Signal transduction mechanisms (78)	*bolA (1)*	-
**M:** Cell wall/membrane/ envelope biogenesis (128)	-	*lepA, lpxP (2)*
**O:** Posttranslational modification, protein turnover, chaperones (114)	*clpB, clpS, degP, degQ, dnaJ, dnaK, groL, groS, grpE, hslO, htpG_1, htpX, loiP, lon, lon1, ppiD, slyD, tig (18)*	*degP, degQ, hscA (3)*
**C:** Energy production/ conversion (167)	*pka (1)*	-
**G:** Carbohydrate transport/ metabolism (59)	-	*otsA, suhB (2)*
**H:** Coenzyme transport/ metabolism (90)	*moaA (1)*	-
**I:** Lipid transport/ metabolism (93)	-	*fabF (1)*
**S:** Function unknown (597)	*hflX, htpG_2, ybeY (3)*	-
**Total genes**	**29**	**16**

Supplementary Table S3. Phenotypic characteristics of *Psychrobacter* sp. SC65A.3 from Scarisoara Ice Cave and other *Psychrobacter* species.

**Characteristics**	***Psychrobacter species***												
	SC65A.3	***1****	***2****	***3****	***4***	***5***	***6***	***7****	***8***	***9****	***10****	***11***	***12****
Habitat	*Cave ice, Romania*	*Cryopeg, Siberia*	*Permafrost, Siberia*	*Sea ice, Antarctica*	*Sea water, South Korea*	*Fermented seafood*	*Antarctic mud*	*Coastal sea-ice, Japan*	*Lamb lungs*	*Sea water, Japan*	*Cryopeg, Arctic*	*Ice core, Arctic*	*Pork sausage*
Growth temperature (min°C-max°C)	4 - 15	-10 - 30	-10 - 28	-18 - 22	4 - 34	4 - 32	4 - 30	4 - 37	30 - 37	0 – 35	-2 – 37	4 - 34	-10 – 27
Optimum temp. (°C)	na	22	22	13 - 15	na	25 - 30	na	25 - 28	na	25	16 – 18	25 - 29	18 – 20
NaCl tolerance (M)	0 – 1.9	0 – 1.7	0.01 - 1.25	0 - 2	0 - 2	2 - 10	0 – 1.6	0 – 1.7	1.1	0 – 1.7	0 – 1.7	0 – 1.4	0 – 1.5
API 20NE													
Nitrates reduction	+	-	+	+	-	+	+	+	+	+	+	-	+
Glucose fermentation	-	-	-	na	+	-	-	-	-	-	na	-	-
Urease	+	-	-	-	-	-	-	+	-	-	+	-	+
Hydrolysis (esculin)	+	-	-	-	-	-	-	-	-	-	na	-	-
Assimilation													
D-glucose	-	-	-	-	+	-	-	-	-	-	-	-	-
Capric acid	-	-	-	+	na	na	-	-	-	-	+	-	+
Malate	-	-	-	-	+	na	+	-	-	+	+	-	+
Trisodium citrate	+	+	-	-	-	na	+	-	-	-	+	-	-
API ZYM													
Alkaline phosphatase	+	+	+	-	+	+	+	na	-	na	+	+	-
Esterase (C4)	+	+	+	+	+	-	+	na	+	na	+	+	-
Lipase (C14)	+	+	+	+	-	-	+	na	-	na	-	-	-
Valine arylamidase	+	-	+	-	-	+	-	na	+	na	-	-	-
Cystine arylamidase	+	-	-	-	-	-	-	na	+	na	-	-	-
Acid phosphatase	-	-	+	-	-	-	-	na	-	na	+	+	-
Naphthol-AS-BI-phosphohydrolase	+	+	+	+	-	+	-	na	-	na	+	+	na
α-galactosidase	-	-	-	-	-	-	-	na	-	na	+	-	-
N-acetyl-β-glucosaminidase	-	-	-	-	-	-	-	na	-	na	-	-	-
α-mannosidase	-	-	-	-	-	-	-	na	-	na	+	-	-
α-fucosidase	-	-	-	-	-	-	-	na	-	na	+	-	-

Reference strains: (1) *P. cryohalolentis* K5 (Bakermans et al., 2006); (2) *P. arcticus* 273-4 (Bakermans et al., 2006); (3). *P. glacincola* DSM12194 (Bowman JP et al., 1997); (4) *P. aquimaris* SW-210 (Yoon et al., 2005b); (5) *P. cibarius* JG-219 (Jung et al., 2005); (6) *P. luti* NF11 (Bozal et al., 2003); (7) *P. maritimus* Pi2-20 (Romanenko et al., 2004); (8) *P. pulmonis* S-606 (Vela et al., 2003); (9) *P. okhotskensis* MD17 (Yumoto et al., 2003); (10) *P. muriicola* 2pS (Shcherbakova et al., 2009); (11) *P. glaciei* BIc20019 (Zeng et al., 2016); (12) *P. urativorans* DSM14009 (Bowman et al., 1996). (*) psychrophilic species; na = not available.

Supplementary Table S4. Genomic characteristics and comparative analyses of *Psychrobacter* species genes

Bacterial strains	Genome characteristics				Comparative analyses		
	**Length (bp)**	**G+C (%)**	**ANI (%)**	**ID Genbank**	**CDS**	**rRNA**	**tRNA**
Psychrobacter sp. SC65A.3	3,046,103	42.5	-	CP106752	2,536	15	50
P. aestuarii SC35	2,796,792	49.5	73.46	GCA_904846035	2,324	15	47
P. alimentarius PAMC 27889	3,349,444	43	78.15	CP014945	2,703	15	50
*P. aquaticus CMS56**	3,216,409	43	78.34	GCA_000471625	2,667	3	42
P. aquimaris SW-210	3,441,953	43	80.14	GCA_904845885	2,905	8	47
*P. arcticus 273-4**	2,650,701	43	88.13	CP000082	2,148	12	48
P. arenosus R7	3,696,886	45	73.18	LR884459	2,864	12	48
P. celer 91	2,991,191	47	77.00	GCA_014897715	2,480	4	39
P. cibarius DSM 16327	3,242,921	43	80.32	CP131612	2,670	3	43
P. ciconiae 176-10	2,484,493	45.5	73.03	GCA_904846055	2,136	12	48
P. coccoides F1192	3,054,724	45	76.07	GCA_017498085	2,551	6	41
P. communis Sa4CVA2	2,928,498	43.5	79.92	GCA_014836505	2,398	3	42
*P. cryohalolentis K5**	3,101,097	42.5	96.63	CP000323	2,524	12	48
P. faecalis H5	3,267,216	43.5	79.60	GCA_014897785	2,726	6	42
P. fjordensis BSw21516B	3,443,229	42.5	78.34	GCA_904845995	2,746	18	53
P. fozii NF23	3,499,339	42.5	78.54	GCA_904846015	2,818	7	43
*P. frigidicola ACAM304**	2,846,672	42	76.31	GCA_007997305	2,342	7	42
*P. fulvigenes KC40**	3,465,773	44	77.07	GCA_904846155	2,831	12	48
P. glaciei BIc20019	3,350,593	43.5	79.42	GCA_904846105	2,743	5	43
*P. glacincola DSM12194**	3,247,549	42.5	88.06	GCA_904846215	2,625	8	44
P. halodurans F2608	2,943,030	47.5	77.12	GCA_017498075	2,333	3	42
*P. immobilis DSM7229**	3,243,066	43	80.32	GCA_003148585	2,640	6	43
P. jeotgali YKJ-103	3,138,794	42.5	75.82	GCA_904846315	2,565	9	46
P. luti NF11	2,969,874	42.5	80.66	GCA_904846335	2,413	5	42
P. lutiphocae IMMIBL-1110	3,211,876	41.5	72.44	GCA_904846305	2,544	12	54
P. maritimus Pi2-20	3,161,836	43	80.87	CP124526	2,563	3	42
*P. muriicola 2pS**	3,350,432	43	79.58	GCA_904846535	2,820	18	53
P. namhaensis SW-242	2,824,513	45	78.21	GCA_904846355	2,323	6	44
*P. nivimaris 88/2-7**	3,394,877	43	77.96	GCA_904846365	2,798	9	44
*P. okhotskensis MD17**	3,449,090	43.5	79.39	OZ281822	2,803	3	42
*P. pacificensis WSYP01**	2,995,423	44	77.49	CP048752	2,458	15	50
P. pasteurii A1019	2,941,644	42.5	72.63	GCA_900162815	2,514	7	43
P. phenylpyruvicus ACAM535	3,275,513	42	72.30	GCA_904846425	2,629	15	54
P. piechaudii 1232	2,820,896	42.5	72.31	GCA_900162825	2,387	5	40
P. piscatorii T-3-2	3,514,701	43.5	77.71	GCA_904846415	2,936	12	46
P. pocilloporae S6-60	3,123,703	44	77.66	GCA_029872915	2,567	15	51
P. proteolyticus 116	3,037,410	43	77.76	GCA_904846455	2,485	9	42
P. pulmonis S606	2,976,851	43.5	80.08	GCA_904846465	2,410	5	42
*P. pygoscelis ISTPP5b**	3,407,407	44.5	72.65	GCA_004335015	2,720	6	45
P. raelei PraFG1	3,165,292	44.5	72.80	CP093310	2,545	15	57
P. saeujeotis FBL11	3,293,859	42.5	76.97	GCA_039615135	2,688	4	42
P. sanguinis FDAARGOS1598	3,395,670	41.5	72.58	CP085990	2,738	15	54
*P. submarinus KMM225**	3,013,331	45	75.09	GCA_904846685	2,513	4	42
*P. urativorans DSM14009**	3,458,123	41.5	76.83	CP012678	2,787	6	40
*P. vallis CMS39**	3,222,629	43.5	78.36	GCA_904846715	2,646	3	41
*Psychrobacter sp. G**	3,113,999	42.5	96.84	CP006265	2,564	12	48

ANI% (average nucleotide identity) indicating similarity measure of nucleotides between two genomes; (bp) base pairs; (*) psychrophilic species

Supplementary Table S5. Summary of proteolytic and hydrolytic enzymes in *Psychrobacter* SC65A.3 genome annotated by functional Group and EC Class. A total of 36 genes correspond to hydrolases (EC class 3), and 3 additional genes encode non-hydrolase proteins that are either misannotated or functionally associated with hydrolytic processes.

Functional Group	Gene Name	Enzyme / Protein Name	EC Number	EC Class Name	Key Domains/Families	COG/Pfam	# Copies	Notes / Specificity
**Serine Proteases (S33)**	-	Serine aminopeptidase, S33	3.4.11.–	3.4: Peptide hydrolases	Abhydrolase_1	COG2267	3	Membrane-associated; cleaves N-terminal amino acids
**Metallopeptidase M15**	-	Peptidase M15	3.4.–.–	3.4: Peptide hydrolases	Peptidase_M15_3	COG3108	1	Zn^{2+}-dependent; peptidoglycan remodeling
**Metallopeptidase M16**	*insA*	Insulinase	3.4.24.56	3.4: Peptide hydrolases	Peptidase_M16, Peptidase_M16_C	COG0612	1	Mitochondrial processing enzyme
**Metallopeptidase M20**	-	Peptidase dimerisation domain (M20-like)	3.4.–.–	3.4: Peptide hydrolases	M20_dimer, Peptidase_M20	COG1473	1	Exopeptidase (N-terminal amino acid hydrolysis)
**Serine Protease (DegP/HtrA)**	*htrA*	Tail-specific protease	3.4.21.102	3.4: Peptide hydrolases	PDZ, Peptidase_S41	COG0793	1	Chaperone-protease; stress-induced
**Metallopeptidase M61**	-	M61 glycyl aminopeptidase	3.4.11.14	3.4: Peptide hydrolases	Peptidase_M61	COG3975	1	Specific for glycine at N-terminus
**Rhomboid Protease**	-	Rhomboid protease	3.4.21.–	3.4: Peptide hydrolases	Rhomboid	COG0705	1	Intramembrane serine protease
**Metallopeptidase M23**	*nlpD*	NlpD (peptidoglycan endopeptidase)	3.4.–.–	3.4: Peptide hydrolases	LysM, Peptidase_M23	COG1388/ COG4942	1	Cell separation; peptidoglycan cleavage
**Metallopeptidase M24**	*map/ pepA*	Methionine aminopeptidase/Leucyl aminopeptidase	3.4.11.9/ 3.4.11.1	3.4: Peptide hydrolases	Peptidase_M24	COG000/ COG01255	2	AMP processing (map), leucine-specific (pepA)
**Metallopeptidase M90**	-	Peptidase M90	3.4.–.–	3.4: Peptide hydrolases	Peptidase_M90	–	1	Predicted metallopeptidase
**Esterases/Lipases**	-	GDSL-like lipase/esterase	3.1.1.–	3.1: Acting on ester bonds	GDSL-like Lipase/ Acylhydrolase	COG2755	1	Broad substrate specificity
**Esterases/Lipases**	*fgh*	Esterase FGH	3.1.2.12	3.1: Acting on ester bonds	Esterase, CE1	COG0627	1	Formaldehyde detoxification
**Esterases/Lipases**	-	Steryl acetyl hydrolase	3.1.1.–	3.1: Acting on ester bonds	Abhydrolase_3	COG0657	1	Cholesterol ester hydrolysis
**α/β-Hydrolases**	-	Alpha/beta hydrolase	3.1.–.–	3.1: Ester bonds (general hydrolase fold)	Abhydrolase_1/6, Hydrolase_4, DUF676, PGAP1	COG2267/ COG1073/ COG1075	4	Diverse substrates; broad esterase/amidase activity

**Hydrolases (Amidases)**	*pvdQ*	Penicillin amidase	3.5.1.97	3.5: Hydrolases acting on carbon–nitrogen bonds	Penicil_amidase	COG2366	1	Catalyzes hydrolysis of the amide bond between 6-aminopenicillanic acid and its acyl side chain; linked to penicillin degradation
**HAD Superfamily**	*gph*	HAD phosphatase (sugar/phosphate)	3.1.3.18	3.1: Phosphoric monoester hydrolases	HAD_2	COG0546	2	Sugar/phosphate metabolism
**β-lactam resistance (β-lactamase)**	*bla*	Beta-lactamase	–	Hydrolase (β-lactam ring hydrolysis)	Beta-lactamase_B	COG1680	3	Hydrolyzes β-lactam antibiotics; confers resistance to penicillins and cephalosporins
**β-lactam resistance (β-lactamase)**	*blh*	Metallo-β-lactamase superfamily	–	Metallo-hydrolase	Lactamase_B	COG0491	2	Zinc-dependent β-lactamase; degrades carbapenems and cephalosporins
**β-lactam resistance**	*ampC*	Class C β-lactamase (AmpC-type)	3.5.2.6	3.5: Hydrolase acting on carbon–nitrogen bonds	Lactamase_B	COG1680	1	Confers resistance to cephalosporins; typically, chromosomal and inducible
**β-lactam resistance**	*DUF302*	Domain of unknown function (β-lactamase-associated)	–	–	DUF302	COG3439	1	Hypothetical β-lactamase-like protein; possibly structural or regulatory role
**β-lactam resistance**	*gloB*	Thiolesterase / Glyoxalase II	3.1.2.6	3.1: Hydrolase (thioester bond)	HAGH_C, Lactamase_B	COG0491	1	May contribute to detoxification and stress response; structurally related to β-lactamases
**β-lactam resistance**	*rnz*	Zinc phosphodiesterase (RNase Z-like)	3.1.26.11	3.1: Endonuclease/ RNase	Lactamase_B_2	COG1234	1	Involved in tRNA maturation; evolutionarily related to metallo-β-lactamases
**Antibiotic resistance (Hydrolases)**	*uppP (bacA)*	Undecaprenyl diphosphatase	3.6.1.27	3.6: Hydrolase (phosphoric monoester)	BacA family	COG1396	1	Hydrolyzes undecaprenyl diphosphate (C_{55}-PP); confers bacitracin resistance
**HAD Superfamily**	*phnX*	PhnX family (C–P lyase-associated)	3.11.1.1	3.11: Carbon-phosphorus bond hydrolases	HAD_2, Hydrolase	COG0637	1	C–P bond cleavage
**HAD Superfamily**	-	Haloacid dehalogenase	3.8.1.2	3.8: Acting on halide bonds	HAD_2	COG0546	1	C–halogen dehalogenation
**Amidohydrolases**	*guaD/ atzB*	Amidohydrolase (AtzB/GuaD)	3.5.4.3/ 3.5.4.32	3.5: Carbon-nitrogen bonds (non-peptide)	Amidohydro_1	COG0402	3	Purine/atriazine degradation
**Amidohydrolases**	*ramA*	Carbon-nitrogen hydrolase (RamA)	3.5.5.1	3.5: C–N bonds (linear amides)	CN_hydrolase	COG0388	1	Nitrilase activity

**Phosphodiesterases**	*rnz*	RNase_T (tRNA 3′ processing)	3.1.13.1	3.1: Phosphoric diester hydrolases	Ribonuc_L-PSP	COG0251	1	tRNA maturation
**Phosphodiesterases**	*gdpd*	Glycerophosphodiester phosphodiesterase (GDPD)	3.1.4.46	3.1: Phosphoric diester hydrolases	GDPD	–	1	Cleaves glycerophosphodiesters
**Phospholipases**	-	PLA1-like hydrolase	3.1.1.32/ 3.1.1.4	3.1: Carboxylic ester hydrolases	PLA1	–	1	Phospholipase A1 activity
**Serine Carboxypeptidases**	-	Peptidase_S11 (D-Ala-D-Ala carboxypeptidase)	3.4.16.4	3.4: Peptide hydrolases	Peptidase_S11	–	1	Peptidoglycan biosynthesis
**Non-hydrolase (included for context)**	*mip*	Peptidylprolyl isomerase (PPIase)	5.2.1.8	5.2: Isomerases (cis-trans isomerization)	Rotamase	COG0760	2	Not a hydrolase—protein folding chaperone
**Non-hydrolase (included for context)**	-	Peptidase M22 (Glycoprotease)	2.3.1.234	2.3: Acyltransferases	Peptidase_M22	–	1	Not a hydrolase—misannotated as a peptidase; actually, an acyltransferase
